# Gut microbiota, plasma metabolites, and osteoporosis: unraveling links via Mendelian randomization

**DOI:** 10.3389/fmicb.2024.1433892

**Published:** 2024-07-15

**Authors:** Yi Lu, Xiaobing Cai, Baohua Shi, Haitao Gong

**Affiliations:** Department of Orthopedics, Chongming Branch, Shanghai Tenth People’s Hospital, Shanghai, China

**Keywords:** gut microbiota, osteoporosis, plasma metabolites, Mendelian randomization, mediation analysis

## Abstract

**Objective:**

Osteoporosis, characterized by reduced bone density and heightened fracture risk, is influenced by genetic and environmental factors. This study investigates the interplay between gut microbiota, plasma metabolomics, and osteoporosis, identifying potential causal relationships mediated by plasma metabolites.

**Methods:**

Utilizing aggregated genome-wide association studies (GWAS) data, a comprehensive two-sample Mendelian Randomization (MR) analysis was performed involving 196 gut microbiota taxa, 1,400 plasma metabolites, and osteoporosis indicators. Causal relationships between gut microbiota, plasma metabolites, and osteoporosis were explored.

**Results:**

The MR analyses revealed ten gut microbiota taxa associated with osteoporosis, with five taxa positively linked to increased risk and five negatively associated. Additionally, 96 plasma metabolites exhibited potential causal relationships with osteoporosis, with 49 showing positive associations and 47 displaying negative associations. Mediation analyses identified six causal pathways connecting gut microbiota to osteoporosis through ten mediating relationships involving seven distinct plasma metabolites, two of which demonstrated suppression effects.

**Conclusion:**

This study provides suggestive evidence of genetic correlations and causal links between gut microbiota, plasma metabolites, and osteoporosis. The findings underscore the complex, multifactorial nature of osteoporosis and suggest the potential of gut microbiota and plasma metabolite profiles as biomarkers or therapeutic targets in the management of osteoporosis.

## 1 Introduction

Osteoporosis (OP) represents a systemic skeletal disorder that disproportionately affects postmenopausal women (Compston et al., 2019). This disease manifests through diminished bone mass and deterioration of bone microarchitecture, precipitating increased skeletal fragility and susceptibility to fractures (Ensrud and Crandall, 2017). Coinciding with the demographic shift towards an older global population, the incidence and prevalence of OP are escalating. These trends are having profound consequences for patient health, exerting extensive strain on healthcare infrastructures, and presenting formidable challenges to public health (Sambrook and Cooper, 2006; Rachner et al., 2011). In the United States alone, the economic burden of osteoporosis reached approximately $57 billion in 2018, with projections suggesting a potential doubling of this cost by 2040 (Pinheiro et al., 2020). Consequently, elucidating the etiology of OP, as well as advancing its prevention and therapeutic modalities, has garnered considerable focus as a critical aspect of health care for the middle-aged and elderly populations.

Recent studies have highlighted the significant role of the human gut microbiota (GM) in various physiological processes. The human GM comprises of approximately 100 trillion microbial cells, encompassing an estimated 3.3 million microbial genes (Qin et al., 2010). This complex community has undergone co-evolution with its host and is pivotal in sustaining the host’s health (Bäckhed et al., 2005; Round and Mazmanian, 2009; Brestoff and Artis, 2013). A wealth of both basic and clinical research has established an association between GM and the processes governing bone metabolism, as well as the maintenance of bone density (Sjögren et al., 2012; Ohlsson and Sjogren, 2015; Das et al., 2019; Li et al., 2019). Imbalances in GM have been linked to a variety of diseases, including OP (Shandilya et al., 2022). The notion of a gut-bone axis is gaining traction within the scientific community as an area of considerable interest, underscored by the burgeoning body of research being undertaken (He and Chen, 2022). However, the pathogenesis of OP is acknowledged to be complex and multifactorial. Contributing elements include autophagy dysfunction, imbalances in iron metabolism, the inherent processes of aging, stress factors, and perturbations in GM (Cizza et al., 2009; Tsay et al., 2010; Chen et al., 2017; Xu et al., 2017; Li et al., 2020). These factors interact within a highly intricate network, ultimately influencing the cascade of pathological events characteristic of OP (Song et al., 2022).

Metabolites, small molecules that represent intermediate or end products of metabolic processes, are subject to fluctuations influenced by a range of factors. These include genetic predispositions, dietary habits, lifestyle choices, the composition of GM, and the presence of disease states (Bar et al., 2020; Pietzner et al., 2021). Consequently, metabolites have the potential to modulate disease risk and serve as critical targets for therapeutic interventions (Wishart, 2016). Previous investigations have delineated the relationships between blood metabolites and bone mineral density (BMD). For instance, one study pinpointed 10 blood metabolites that may influence femoral neck BMD (Meng et al., 2018). Subsequently, another analysis revealed eight blood metabolites with a significant impact on hip BMD (Liu et al., 2018), while a more recent study identified 13 plasma metabolites that markedly affect heel BMD (Chen et al., 2023). Consequently, integrating analysis of blood metabolites with GM studies could be instrumental in identifying biomarkers and precise intervention targets for OP. With the rapid advancements in genomics, metabolomics, and macro-genomics of GM, there is a substantial opportunity to achieve the aforementioned objectives.

Although observational studies have identified associations between GM, blood metabolites, and OP, the potential for residual confounders and reverse causation cannot be discounted. Clinical randomized controlled trials (RCTs) represent the gold standard to validate these findings; however, such approaches are often hampered by the high costs involved and ethical considerations, making their implementation challenging (Zabor et al., 2020). Mendelian randomization (MR) is an analytical method that employs genetic variants as instruments to simulate the conditions of an RCT, thereby facilitating the inference of causal relationships between risk factors and diseases. This approach mitigates the impact of confounding variables and reverses causation (Skrivankova et al., 2021). Now that large-scale genome-wide association studies (GWAS) data on GM, blood metabolites, and OP are publicly available (Visscher et al., 2017), these resources afford a unique opportunity to explore the causal relationships between these variables using MR. Few studies have employed MR methods to explore the causal relationship between GM and OP (Tu et al., 2021). While numerous studies have utilized MR to investigate the connection between blood metabolites and BMD (Liu et al., 2018; Meng et al., 2018; Yu et al., 2022; Chen et al., 2023; Chen and He, 2023), research focusing specifically on the OP phenotype is scant. Moreover, comprehensive investigations into the associations among GM, blood metabolites, and OP remain limited.

In this study, we conducted a comprehensive MR analysis to investigate the causal relationships between GM, the plasma metabolites, and OP. We further examined whether the plasma metabolites serve as a mediator in the pathway from GM to OP. Additionally, through reverse MR analysis, we assessed whether genetic predispositions to OP could influence GM and plasma metabolites.

## 2 Materials and methods

### 2.1 Study design

**Figure 1:**
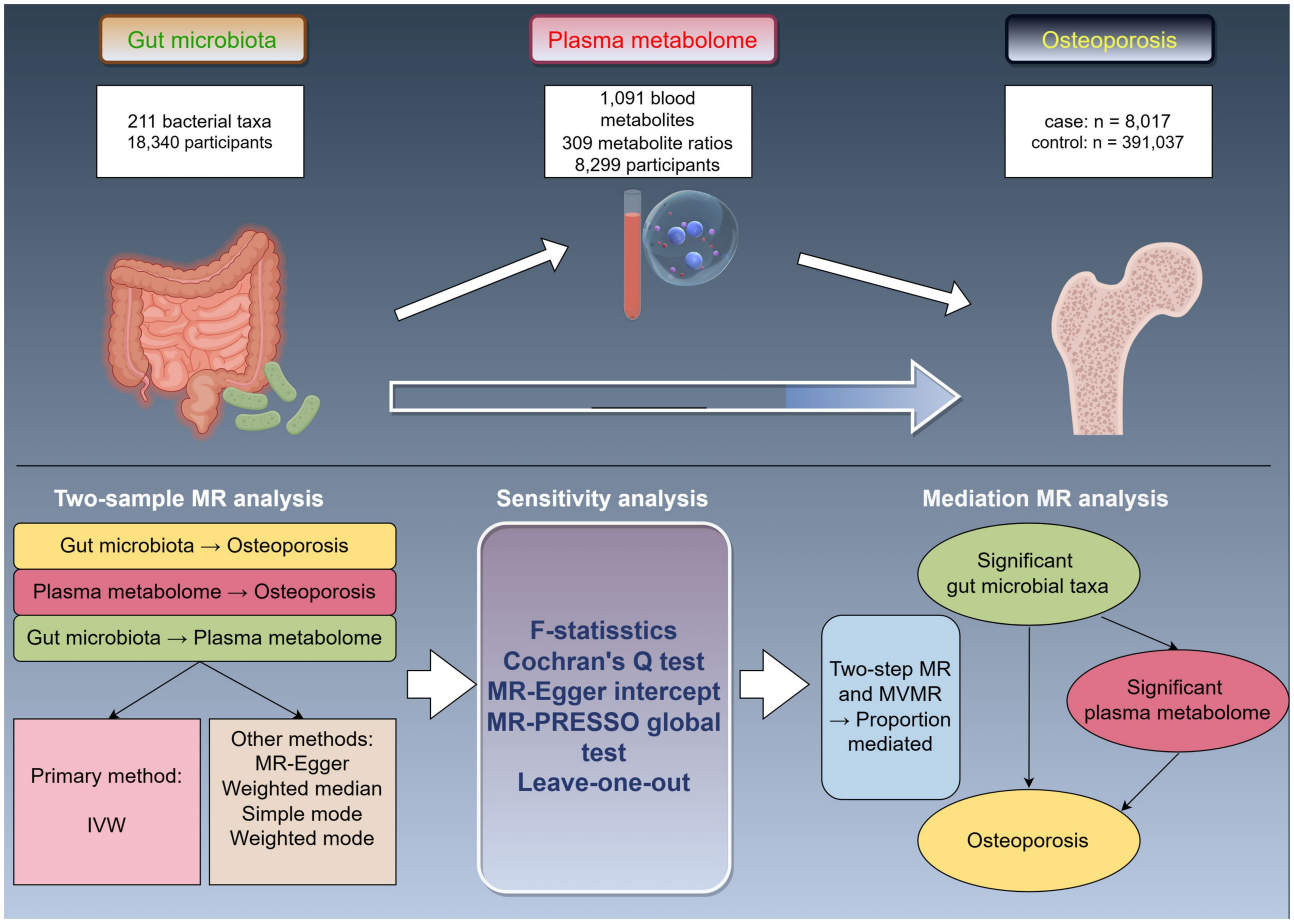
Study design overview. MR, Mendelian randomization; IVW, inverse-variance weighted; MVMR, multivariable Mendelian randomization.

The study design is illustrated in Figure 1 (by Figdraw). Initially, we accessed published GWAS summary data encompassing traits such as gut microbiota, plasma metabolites, and osteoporosis. Subsequently, two-sample MR analyses were employed to assess the causal relationships among these variables. Finally, two-step and multivariable MR (MVMR) analyses were utilized to explore the mediating effects of plasma metabolites on the association between gut microbiota and osteoporosis.

### 2.2 Data sources

Gut microbiota data analyzed in this study were sourced from the MiBioGen consortium, which curated and examined genome-wide genotypes alongside 16S rRNA fecal microbiome profiles from a cohort of 18,340 individuals distributed across 24 distinct cohorts. These data are accessible via.^1^ The comprehensive dataset encompasses 211 gut microbial taxa, broken down into 131 genera, 35 families, 20 orders, 16 classes, and 9 phyla (Kurilshikov et al., 2021). Among these, 15 taxa belonging to unidentified families or genera were excluded from the dataset. Consequently, the dataset for MR analysis was narrowed down to 196 taxa. GWAS data pertinent to plasma metabolites were sourced from the GWAS Catalog, with specific study IDs ranging from GCST90199621 to GCST90201020, and are accessible at: https://www.ebi.ac.uk/gwas/downloads/summary-statistics. The dataset includes data on 1,091 blood metabolites and 309 metabolite ratios. The chemical properties of metabolites denoted with ‘X-’ remain unknown. This dataset is derived from 8,299 samples, and it encompasses approximately 150,000 single nucleotide polymorphism (SNP) sites (Chen et al., 2023). The GWAS summary data for osteoporosis were sourced from the tenth release of the FinnGen consortium, accessible at: https://r10.risteys.finngen.fi/ (Kurki et al., 2023).

### 2.3 Instrumental variable selection

To accurately estimate causal effects using genetic instruments, three fundamental assumptions of instrumental variables (IVs) must be met: (1) IVs must be associated with the exposure factors; (2) IVs must not be linked to any confounding variables; and (3) IVs should influence the outcome variables solely through their impact on exposure factors (Bowden and Holmes, 2019). In this study, IVs were stringently screened under specific criteria to ensure validity: (1) We adopted a significance threshold of *p* < 1 × 10^−5^ for selecting SNPs associated with the GM. These SNPs were used as genetic instrumental variables, consistent with methodologies from prior gut microbiota MR studies (Sanna et al., 2019), where *p* < 1 × 10^−5^ is established as the optimal threshold for identifying genetic predictors of gut microbial traits. Similarly, for plasma metabolites, a more stringent threshold of *p* < 5 × 10^−8^ was applied to select genetic predictive factors. (2) A clumping process was undertaken to mitigate linkage disequilibrium, selecting SNPs with r^2^ < 0.001 and distance of ±10,000 kilobases (kb) (GM) (Myers et al., 2020), and plasma metabolites selecting SNPs with r^2^ < 0.1 and distance of ±500 kb (Yang et al., 2020; Yu et al., 2022). (3) The strength of the selected SNPs was assessed via the F-statistic, and SNPs with an F-statistic <10 were excluded to minimize weak instrument bias in the MR analysis (Burgess and Thompson, 2011). (4) In cases where IVs were absent in the outcome dataset, proxies with r^2^ > 0.8 were incorporated. Additionally, to ensure that the effects of the selected SNPs on exposure and outcome correspond to the same alleles, palindromic SNPs were removed during the harmonization process.

## 3 Statistical analysis

### 3.1 Two-sample MR

Two-sample MR methods were used to assess the causal relationship between the GM, plasma metabolites, and OP. We implemented a range of analytical techniques including inverse-variance weighted (IVW), MR-Egger (Burgess and Thompson, 2017), weighted median (WM) (Bowden et al., 2016), simple mode, and weighted mode (Shardell and Ferrucci, 2016) approaches. The IVW method served as the primary analytical tool, with the Wald ratios test applied in cases where only one instrumental variable (IV) was available (Burgess et al., 2013). MR results were presented as odds ratios (ORs) with accompanying 95% confidence intervals (CIs). Statistical significance was established when the *P*_IVW_ < 0.05, along with a consistent directional agreement among the IVW, MR-Egger, and WM results. Additionally, to address the potential increase in Type I error due to multiple comparisons, we applied the false discovery rate (FDR) correction employing the Benjamini-Hochberg procedure to the primary IVW results (Benjamini and Hochberg, 1995). A significance threshold was set at FDR < 0.1 for a significant association (Storey and Tibshirani, 2003). Conversely, a result with *P*_IVW_ < 0.05, but FDR > 0.1 was considered to indicate a suggestive association.

### 3.2 Sensitivity analysis

We employed Cochran’s Q test to assess the heterogeneity across each SNP (Cohen et al., 2015). To evaluate potential horizontal pleiotropy effects, we utilized both the MR-PRESSO global test and the MR-Egger intercept. MR-PRESSO was used to detect significant outliers and to correct the horizontal plural effect by removing outliers (Verbanck et al., 2018). Furthermore, a leave-one-out analysis was conducted to assess whether causality estimates were influenced by any single SNP (Hemani et al., 2018).

### 3.3 Reverse MR analysis

To investigate the causal effects of OP on GM and plasma metabolites (*P*_IVW_ < 0.05), reverse MR analyses were conducted separately. For these analyses, SNPs associated with OP were utilized as IVs, with OP considered as the exposure, and both GM and plasma metabolites assessed as outcomes. The methodological framework for the reverse MR analyses mirrored that of the standard MR analyses.

### 3.4 Mediation analysis

Mediation analysis is employed to investigate the pathways through which exposure influences an outcome, thereby illuminating potential underlying mechanisms (Carter et al., 2021). In this study, the mediation analysis focused on exploring how osteoporosis-associated changes in GM and plasma metabolites interrelate. Initially, a two-sample MR approach was applied to assess the causal relationships between GM and OP, resulting in a calculation of the total effect represented by coefficient *β*. Subsequently, to further investigate the role of plasma metabolites as potential mediators in the effects of GM on OP, a two-step MR approach was employed. This analysis involved examining the causal relationship between plasma metabolites and OP, as well as the causal relationship between GM and plasma metabolites, the latter providing the regression coefficient (*β*1). In the third phase, MVMR was utilized to discern which plasma metabolites maintained a causal relationship with OP, independent of the effects attributed to GM. This analysis yielded coefficient *β*2. To quantify the mediating effect, we applied a two-step MR methodology, where the mediating effect was defined as *β*1 × *β*2. Finally, the mediator ratio was calculated with the formula: mediator ratio = (*β*1 × *β*2 / *β*) × 100%, providing a percentage that represents the proportion of the total effect mediated by the identified pathways.

All statistical analyses were performed using the “TwoSampleMR” package (Hemani et al., 2018) and the “MR-PRESSO” package (Ong and MacGregor, 2019) in R version 4.3.2.

R code used for the data analysis is available in the Supplementary material.

## 4 Results

### 4.1 Instrumental variables

We selected valid instrumental variables from GWAS of the GM and plasma metabolites, applying the previously specified selection criteria. Detailed characteristics of these IVs are presented in Supplementary Tables S1, S2. All SNPs utilized in the analyses demonstrated an F-statistic greater than 10, ensuring sufficient statistical power and reducing the risk of weak instrument bias.

### 4.2 Causal effects of gut microbiota on osteoporosis

**Figure 2:**
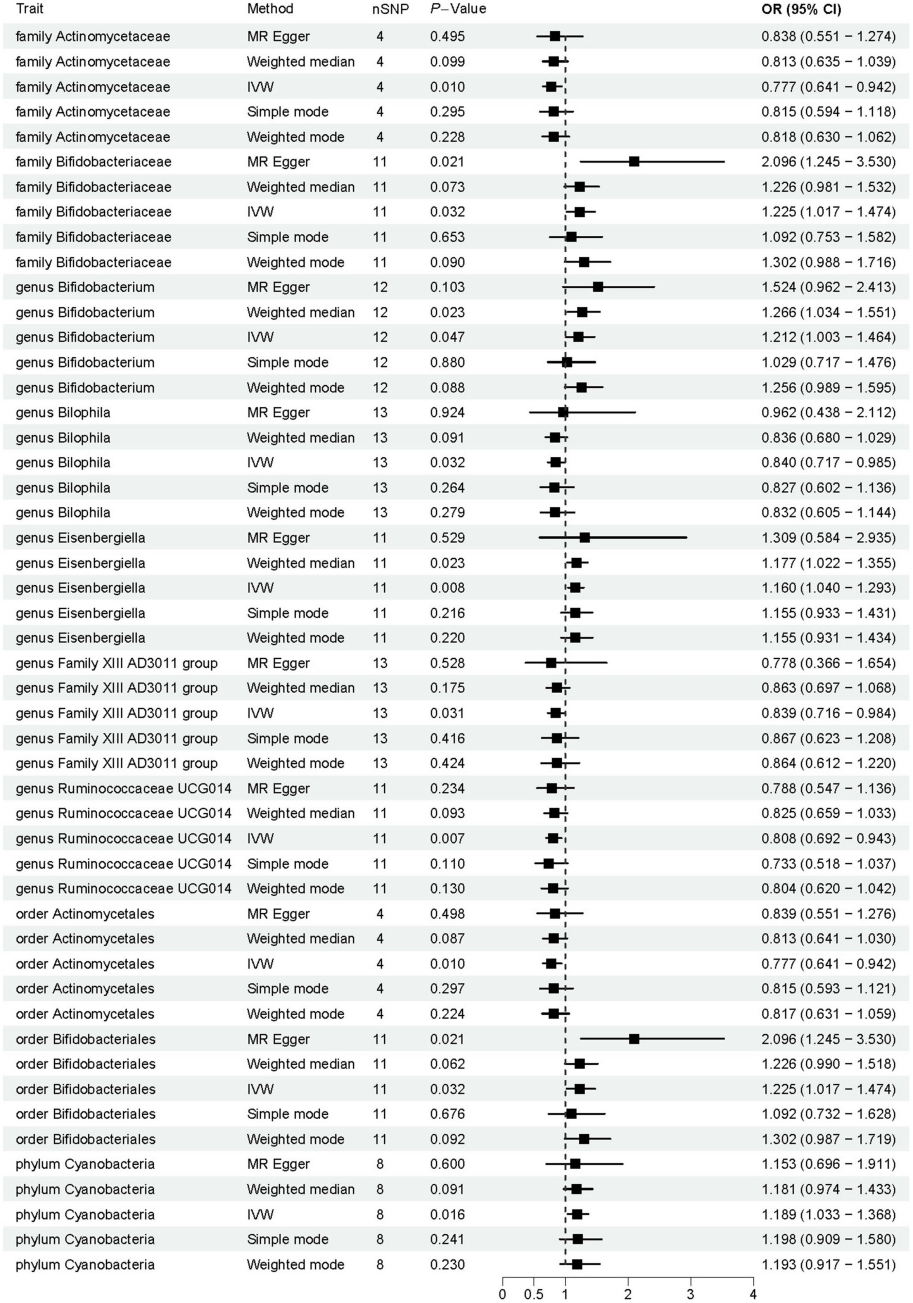
Results of Mendelian randomization analysis of the gut microbiota associated with osteoporosis. IVW, inverse-variance weighted.

**Figure 3:**
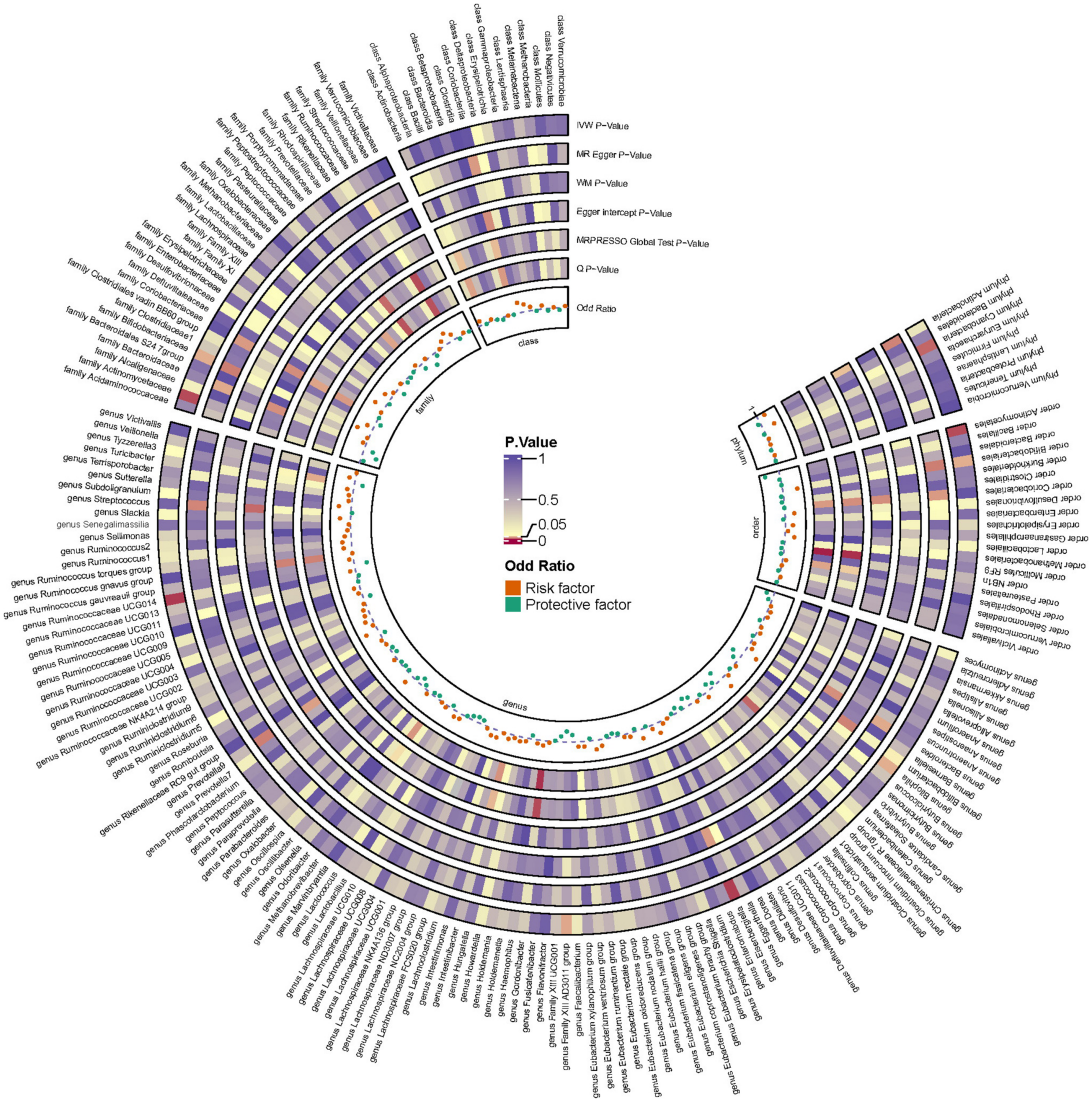
Circular heat map depicting suggestive genetic correlations between gut microbes and osteoporosis. Each segment of the circle represents a specific microbe, and the color intensity indicates the strength of the genetic correlation with osteoporosis. Detailed annotations and the scale of correlation values are provided alongside the heat map.

Employing a two-sample MR approach, we identified 10 suggestive associations between GM and OP (*P*_IVW_ < 0.05, FDR > 0.1). Notably, the *family Bifidobacteriaceae*, *genus Bifidobacterium*, *genus Eisenbergiella*, *order Bifidobacteriales*, and *phylum Cyanobacteria* were positively associated with the risk of OP. *Family Actinomycetaceae*, *genus Bilophila*, *genus Family XIII AD3011 group*, *genus Ruminococcaceae UCG014*, and *order Actinomycetales* were reducing the risk of OP. Further details of these associations can be found in Figure 2 and Supplementary Table S3. Sensitivity analysis was conducted to confirm the robustness of the MR results, as detailed in Figure 3 and Table 1. Detailed data for the circular heat map are given in Supplementary Table S12. Cochran’s Q test was performed and revealed no significant heterogeneity among the instrumental variables used, underpinning the consistency of our findings. Furthermore, both MR-Egger regression and MR-PRESSO analyses indicated no evidence of horizontal pleiotropy, thereby supporting the validity of the causal inferences derived from our MR study. Detailed information on forest plots, scatter plots, funnel plots, and leave-one-out plots, utilized in the two-sample MR analysis exploring the effect of GM on OP, is presented in Supplementary Figures S1–S4. For the above-identified potential causal relationship between GM and OP, reverse MR analysis was conducted. This analysis did not identify a causal relationship in the reverse direction. Further details of this analysis are provided in Supplementary Table S4.

**Table 1:** Sensitivity analysis of the gut microbiota taxa associated with osteoporosis.

Exposure(s)	GWAS ID	Heterogeneity test (IVW)		MR-Egger intercept test		MR-PRESSO global test
		Cochran ‘s Q	*p*-value	Intercept	*p*-value	*p*-value
Family Actinomycetaceae	ebi-a-GCST90016925	1.806	0.614	−0.010	0.729	0.687
Family Bifidobacteriaceae	ebi-a-GCST90016929	14.398	0.156	−0.041	0.063	0.221
Genus Bifidobacterium	ebi-a-GCST90016970	19.737	0.049	−0.020	0.309	0.078
Genus Bilophila	ebi-a-GCST90016971	3.324	0.993	−0.010	0.738	0.991
Genus Eisenbergiella	ebi-a-GCST90016991	4.613	0.916	−0.013	0.773	0.931
Genus family XIII AD3011 group	ebi-a-GCST90017008	9.441	0.665	0.006	0.844	0.693
Genus Ruminococcaceae UCG014	ebi-a-GCST90017061	8.899	0.542	0.002	0.888	0.620
Order Actinomycetales	ebi-a-GCST90017090	1.816	0.611	−0.010	0.727	0.705
Order Bifidobacteriales	ebi-a-GCST90017093	14.398	0.156	−0.041	0.063	0.190
Phylum Cyanobacteria	ebi-a-GCST90017112	6.075	0.531	0.004	0.906	0.570

GWAS, Genome-Wide Association Studies; IVW, Inverse variance weighted.

### 4.3 Causal effects of plasma metabolites on osteoporosis

**Figure 4:**
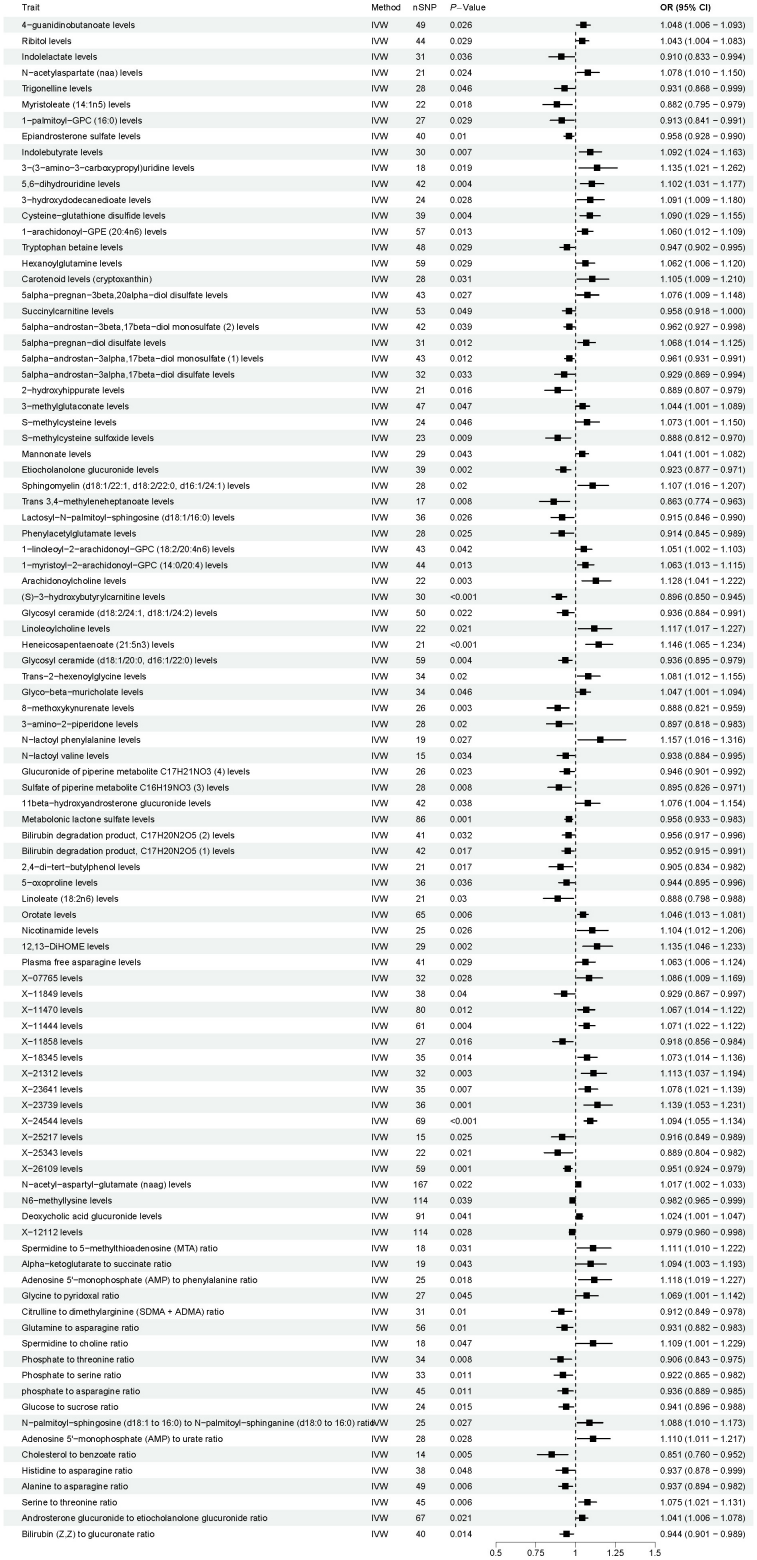
Results of Mendelian randomization analysis of the plasma metabolites associated with osteoporosis. IVW, inverse-variance weighted.

As shown in Figure 4, according to the IVW method, the results showed 96 causal relationships between plasma metabolomics and OP (*P*_IVW_ < 0.05). Notably, a significant positive causal relationship with OP was observed for *X-24544* levels (OR = 1.094, 95% CI [1.055–1.134], *p* < 0.001, FDR < 0.1). Conversely, *(S) − 3 − hydroxybutyrylcarnitine* levels were significantly negatively associated with OP (OR = 0.896, 95% CI [0.850–0.945], *p* < 0.001, FDR < 0.1). The other 94 plasma metabolites were all identified as suggestive associations with OP (*P*_IVW_ < 0.05, FDR > 0.1). Detailed findings from the two-sample MR analysis of plasma metabolites and OP are presented in Supplementary Table S5. Furthermore, sensitivity analyses confirmed the absence of heterogeneity and horizontal pleiotropy in these findings, as detailed in Supplementary Tables S6, S7. Detailed information on the various visual analyses used in the two-sample MR study exploring the causal effects of plasma metabolites on OP is provided in the Supplementary material. This includes forest plots, scatter plots, funnel plots, and leave-one-out plots, respectively depicted in Supplementary Figures S5–S8. To assess the potential causal relationship between plasma metabolites and OP identified by forward MR, a reverse MR analysis was conducted. This analysis found no evidence of a causal relationship in the reverse direction. Detailed results from this reverse MR analysis are available in Supplementary Table S8.

### 4.4 Causal effects of gut microbiota on plasma metabolites

Following the two-step process described earlier, we identified sets of positive exposure IDs for both GM to OP and plasma metabolites to OP. Subsequently, these sets were analyzed using two-sample MR to identify IDs that may act as mediators of the effect, with significant results (*P*_IVW_ < 0.05). The corresponding results are detailed in Supplementary Tables S9, S10.

### 4.5 Mediation analysis results

To investigate the potential mechanisms behind the onset and progression of OP, we conducted mediation analyses to delineate plasma metabolite-mediated causal pathways between GM and OP. Using the findings from our earlier analyses, we identified specific plasma metabolites that might serve as mediators. We then examined the direct effects of GM on OP, adjusting for these mediating plasma metabolites using MVMR, detailed in Supplementary Table S11. The analysis revealed that six GM taxa retained a significant direct effect on OP, even after considering the mediating effects of plasma metabolites. Additionally, our results uncovered 10 mediating relationships involving seven distinct plasma metabolites in the causal pathway from GM to OP. To evaluate the mediation effect, we applied the coefficient product method for our calculations. These calculations revealed that each of two unknown plasma metabolites mediated the effects of each of two distinct gut microbiota taxa. Additionally, one mediator was identified for each of the two gut microbiota taxa. Furthermore, two mediators were found to exert suppression effects on the relationships studied. Detailed results are presented in Table 2.

**Table 2:** Results of the mediation effect of the plasma metabolites between the gut microbiota and osteoporosis.

Gut microbiota taxa(GWAS ID)	Plasma metabolites (GWAS ID)	Beta	95%CI	SE	*p*-value	Mediation proportion
Family Actinomycetaceae (ebi-a-GCST90016925)	X-18345 levels (GCST90200556)	−0.03	−0.072, −0.002	0.018	0.010	11.9%
family Actinomycetaceae (ebi-a-GCST90016925)	X-25343 levels (GCST90200666)	−0.033	−0.08, −0.002	0.02	0.007	13.1%
genus Bilophila (ebi-a-GCST90016971)	S-methylcysteine sulfoxide levels (GCST90199907)	−0.022	−0.051, −0.001	0.013	0.001	12.64%
genus Eisenbergiella (ebi-a-GCST90016991)	S-methylcysteine sulfoxide levels (GCST90199907)	0.023	0.005, 0.047	0.011	0.006	15.54%
genus Family XIII AD3011 group (ebi-a-GCST90017008)	3-(3-amino-3-carboxypropyl)uridine levels (GCST90199766)	−0.039	−0.09, −0.002	0.023	0.036	22.29%
Genus family XIII AD3011 group (ebi-a-GCST90017008)	5,6-dihydrouridine levels (GCST90199767)	−0.022	−0.05, −0.003	0.012	0.016	12.57%
Genus family XIII AD3011 group (ebi-a-GCST90017008)	(S)-3-hydroxybutyrylcarnitine levels (GCST90200088)	0.02	0.001, 0.047	0.012	0.015	Suppression effect
Order Actinomycetales (ebi-a-GCST90017090)	X-18345 levels (GCST90200556)	−0.03	−0.072, −0.002	0.018	0.010	11.9%
Order Actinomycetales (ebi-a-GCST90017090)	X-25343 levels (GCST90200666)	−0.033	−0.08, −0.002	0.02	0.007	13.1%
Phylum Cyanobacteria (ebi-a-GCST90017112)	Linoleate (18:2n6) levels (GCST90200354)	−0.027	−0.065, 0	0.017	0.005	Suppression effect

GWAS, Genome-Wide Association Studies; CI, confidence interval.

## 5 Discussion

In this extensive MR analysis, we identified ten gut microbiota taxa weakly associated with osteoporosis: five positively and five negatively. Additionally, 96 plasma metabolites were linked to osteoporosis: 48 had weak positive associations, 46 had weak negative associations, one had a strong positive association, and one had a strong negative association. Further mediation analyses revealed six causal pathways from GM to OP involving ten mediating relationships across seven distinct plasma metabolites, including two with inhibitory effects. These findings confirm the significant linkage between GM and OP and highlight the crucial mediating role of plasma metabolites, which may inform future therapeutic strategies. MR causality analysis involves several assumptions, including no pleiotropy and robust instruments, that minimize confounding factors. While our analyses for GM to OP, metabolites to OP, and GM to metabolites each adhere to these assumptions individually, their combined interpretation needs careful consideration. When combining the individual MRs to infer an overarching pathway from GM through metabolites to OP, we assume that the instruments for GM influence OP only through metabolites. While this approach enhances our understanding, it remains subject to potential unmeasured pleiotropy. Future studies should further validate these combined pathways.

Bone, a dynamic organ integral to the human systemic architecture, maintains its metabolic homeostasis in close association with GM (Quach and Britton, 2017; Duffuler et al., 2024). The underlying molecular mechanisms implicated in OP encompass (Ding et al., 2020): (1) intestinal barrier and nutrient absorption (involving short-chain fatty acids). (2) Immune regulation (Th-17 and T-reg cells balance). (3) Regulation of the gut-brain axis (involving 5-Hydroxytryptamine). Emerging evidence increasingly positions gut microbes as pivotal regulators of bone physiology (Ibáñez et al., 2019), suggesting substantial interplay between microbiological activity and bone health (Lyu et al., 2023). GM and its metabolites significantly influence bone metabolism, making them potential targets for osteoporosis prevention and treatment (Han et al., 2024; Zhang et al., 2024). The application of metabolomics has gained substantial traction in the study of OP in humans over recent years. This analytical approach has enabled researchers to identify specific metabolites that are predictive of various aspects related to OP (Lau et al., 2023). These include forecasting the onset of the disease (Miyamoto et al., 2021), identifying low BMD in postmenopausal women (Miyamoto et al., 2018), distinguishing between osteopenia and OP (Aleidi et al., 2021), enhancing the accuracy of fracture risk predictions (Zhang et al., 2021), and revealing the specific changes in the GM characteristic of each type of OP (Qiao et al., 2024). While numerous MR studies have been conducted to investigate the relationship between blood metabolites and BMD (Moayyeri et al., 2017; Liu et al., 2018; Zhang et al., 2021; Chen and He, 2023), there remains a limited number of studies specifically targeting the phenotype of OP. This burgeoning field promises to unravel complex biochemical interactions and pathways influencing bone health, providing a deeper understanding and potentially new avenues for diagnosis and treatment.

The microbial fermentation of dietary fibers results in the production of short-chain fatty acids (SCFAs), which serve as critical modulators of osteoblast metabolism and overall bone mass (Lucas et al., 2018). Notably, SCFAs such as acetic, propionic, and butyric acids contribute to the preservation of bone mass (El-Saadony et al., 2022). The protective mechanism of SCFAs entails the regulation of osteoclast differentiation and the inhibition of bone resorption, both *in vitro* and *in vivo*, while concurrently preserving bone formation. A previous study has demonstrated that increased levels of *lactobacillus* and *bifidobacteria* enhance the absorption of essential minerals—including calcium, magnesium, and phosphorus—thereby potentially raising BMD (Rodrigues et al., 2012). Conversely, another previous study has identified a negative correlation between the presence of *bifidobacteria* and BMD (Xu et al., 2020). Our own study indicates that the *genus Bifidobacterium*, along with the associated *family Bifidobacteriaceae* and the *order Bifidobacteriales*, are positively correlated with an increased risk of osteoporosis, suggesting a promotive role in the disease’s pathogenesis. The variability in research outcomes can be attributed to factors such as age, environmental conditions, and additional biologically relevant variables. Prior research showed that there was an age-related reduction of the *genus Bifidobacterium*, which is a bacterium that down-regulates pro-inflammatory responses in the gut (Odamaki et al., 2016). This observation sheds light on how disruptions in GM and inflammatory responses can influence disease processes in older populations. Furthermore, the expanding body of research on interspecies symbiosis indicates that issues might ensue when diverse genetically distinct organisms interact with a host, particularly under varying environmental conditions (Schluter and Foster, 2012). This phenomenon was observed in the case of *Bifidobacterium* populations, which were reported to be effectively stimulated by a decrease in *Enterobacteriaceae* levels (Odamaki et al., 2016). This observation underscores the possibility of establishing both positive and negative interactions among different gut bacterial species, enhancing our understanding of microbial dynamics within the gut ecosystem. One study showed a higher abundance of *genus Eisenbergiella* in patients with OP (Wei et al., 2021). This is consistent with our findings. However, there are studies with opposite results, which may be related to the production of the metabolite butyrate (SCFA) (Amir et al., 2014). Butyrate is known to influence the functionality of intestinal macrophages by inhibiting histone deacetylase (HDAC), which leads to the downregulation of pro-inflammatory factors including nitric oxide (NO), interleukin-6 (IL-6), and interleukin-12 (IL-12) (Chang et al., 2014). A recent study demonstrated that butyrate ameliorates OP by directly inhibiting osteoclast formation and bone resorption (Dong et al., 2024). The findings from our MR analysis indicate a positive correlation between the *phylum Cyanobacteria* and an increased risk of OP, potentially due to pro-inflammatory effects. Notably, the abundance of *phylum Cyanobacteria* is significantly elevated in patients with ankylosing spondylitis (AS), a condition also characterized by heightened concentrations of pro-inflammatory cytokines (Liu et al., 2022). This suggests that *Cyanobacteria* may play a contributory role in inflammatory processes that are pivotal in both AS and OP. We found that the plasma metabolite *Linoleate (18:2n6)* mediates the inhibitory effects of the *phylum Cyanobacteria* on the causal relationship between this phylum and OP. Although *linoleate (18:2n6)* is found to be negatively associated with OP, the underlying mechanisms driving this association remain unidentified.

Our research indicates that the *family Actinomycetaceae* and the *order Actinomycetales* exhibit a negative association with the risk of OP. This relationship was mediated through the influence of two unidentified plasma metabolites, labeled *X-18345* and *X-25343*. These metabolites contribute to the pathway with mediation proportions of 11.9 and 13.1%, respectively. Further research is necessary to identify these unknown metabolites and understand their specific roles and mechanisms of action. Previous observational studies have suggested an association between *Actinobacteria* and bone health (Odamaki et al., 2016), *Actinobacteria* are negatively correlated with BMD (Xu et al., 2020), and *Actinobacteria* are thought to play a role in regulating intestinal permeability, modulating the immune system, and influencing the gut-brain axis (Binda et al., 2018). Recent research has uncovered the important influence of GM on the nervous system, particularly through the modulation of hormones and neurotransmitters such as 5-HT (Spohn and Mawe, 2017; Park et al., 2018). Yadav et al. found that reducing 5-HT levels with synthetic molecular inhibitors could effectively prevent bone loss typically induced by ovariectomy (OVX) (Yadav et al., 2010). Several studies, reinforcing our findings, indicate that the *genus Ruminococcaceae UCG014* plays a beneficial role in the management and improvement of OP (Liu et al., 2020; Ling et al., 2021; Wei et al., 2024). This beneficial effect aligns with recent research on strontium (Sr), a bioactive element recognized for its potential to enhance bone quality (Schrooten et al., 2003; Wornham et al., 2014). One particular study has suggested that *Ruminococcaceae UCG014* may enhance the bioavailability or efficacy of Sr. in mitigating bone loss (Xi et al., 2023). The potential mechanism could involve *Ruminococcaceae UCG014* affecting the gut environment or directly interacting with Sr. metabolism, thereby enhancing its absorption or modulating its action at the site of bone. Our study uncovered a negative association between the *genus Bilophila* and OP, with *S-methylcysteine sulfoxide* (SMCSO) playing a mediating role in this relationship. Although research specifically regarding the impact of *Bilophila* on OP is scarce, insights can be drawn from studies focusing on its broader health effects. One such study has found a negative correlation between *Bilophila* and pro-inflammatory cytokine IL-6 levels (Du et al., 2021). The ability of *Bilophila* to potentially lower IL-6 levels suggests that it may exert anti-inflammatory effects. Given the crucial role of inflammation in bone resorption and loss, *Bilophila*’s impact may indeed extend to the immunomodulation of physiological responses associated with OP. It has also been shown that bacteria isolated from human feces can reduce the dietary compound SMCSO, which is biotransformed and may provide additional health benefits to the host (Kellingray et al., 2021). However, the role of osteoporosis needs further study. We found a negative association between the *genus Family XIII AD3011* group and osteoporosis, mediated by the plasma metabolites *3-(3-amino-3-carboxypropyl) uridine* and *5,6-dihydrouridine*. These nucleoside modifications are uncommon and their exact roles are not well understood in the context of human health. However, their association with RNA processing and potentially signaling pathways may suggest that they play a part in cellular processes that impact bone metabolism directly or indirectly (Krog et al., 2011). In contrast, plasma metabolites *(S)-3-hydroxybutyrylcarnitine* were found to exert an inhibitory effect. This compound is commonly involved in lipid metabolism and energy production (Han et al., 2023), but its specific mechanism of action in increasing the risk of OP is unknown.

To our knowledge, this is the first study to utilize GWAS summary statistics to elucidate potential causal relationships between GM, plasma metabolites, and OP. Our study rigorously employed multiple common sensitivity analyses and effectively addressed potential confounders and reverse causation issues to enhance the reliability and validity of our findings. Our preliminary results indicate a possible causal relationship between GM and OP, mediated by specific factors. Our preliminary results suggest that there may be a causal relationship between GM and OP, mediated by specific factors. These insights support a theoretical framework for OP management and prevention and provide ideas for innovative treatment strategies. Furthermore, co-regulation of plasma metabolite levels may lead to breakthroughs in OP prevention and treatment. While our study focused on bacterial taxa, it is essential to consider viruses and fungi’s potential roles in host metabolism and bone health (Wang et al., 2023). Viruses and fungi can influence metabolic pathways and immune responses, which may intersect with the mechanisms by which gut bacteria affect OP. Future research should explore these dimensions to provide a more comprehensive understanding of the gut microbiota’s role.

Our study has several limitations worth noting. Firstly, the absence of detailed demographic data, such as age and gender, limits our ability to perform subgroup analyses. Although our findings provide a generalized view of the associations, future studies should incorporate comprehensive demographic information to uncover potentially significant subgroup-specific interactions. Secondly, the majority of our study participants were of European descent, with a minor inclusion of individuals from other ethnic backgrounds. This lack of ethnic diversity may impact the generalizability of our results. Future research should strive to include more diverse populations to validate and extend our findings across different ethnic groups. Moreover, while MR is a robust tool for inferring causal relationships, it is not without limitations. The validity of MR findings hinges on several assumptions that, if violated, could bias the results. Therefore, our analytical results should be viewed as indicative rather than conclusive and warrant further experimental and clinical investigation to corroborate the proposed causal links. Lastly, the reliance on existing datasets and statistical methods, in the absence of biological experiments, is a limitation that needs to be addressed in future work. Validation through laboratory experiments or clinical trials is essential to confirm the causal relationships suggested by our MR analyses.

## 6 Conclusion

In conclusion, through MR of pooled GWAS data, we identified associations between ten GM taxa and OP risk, and 96 plasma metabolites potentially causally related to the disease. Mediation analysis revealed six causal pathways linking GM to OP through ten mediating relationships involving seven plasma metabolites. These findings highlight the multifactorial nature of OP and underscore the potential of GM and plasma metabolites as novel biomarkers or therapeutic targets. Further validation through experimental and clinical research is needed to confirm these associations and explore their therapeutic implications.

## Data availability statement

The original contributions presented in the study are included in the article/Supplementary material, further inquiries can be directed to the corresponding author.

## Ethics statement

The datasets utilized in this study were publicly available, and ethical approval and informed consent had been secured prior to implementation. Consequently, no further ethical approval or informed consent was required for our study.

## Author contributions

YL: Validation, Supervision, Resources, Project administration, Investigation, Formal analysis, Writing – review & editing, Writing – original draft, Visualization, Software, Methodology, Funding acquisition, Data curation, Conceptualization. XC: Writing – review & editing, Supervision, Project administration, Conceptualization. BS: Writing – review & editing, Validation, Supervision, Formal analysis. HG: Writing – review & editing, Validation, Supervision.

## Footnotes

^1^ http://www.mibiogen.org/
