# Supplementary material for: Gut microbiota, plasma metabolites, and osteoporosis: unraveling links via Mendelian randomization
Source: Front Microbiol. 2024 Jul 15;15:1433892. doi: 10.3389/fmicb.2024.1433892 (PMC11284117; doi:10.3389/fmicb.2024.1433892)
Supplement: Supplementary file 2 [file Data_Sheet_1.PDF]

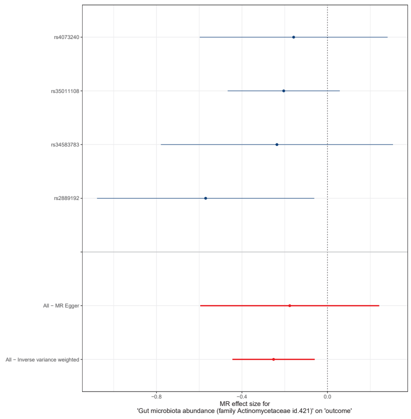
rs4073240
rs35011108
rs34583783
rs2889192
All − MR Egger
All − Inverse variance weighted
−0.8
−0.4
0.0
MR effect size for
'Gut microbiota abundance (family Actinomycetaceae id.421)' on 'outcome'

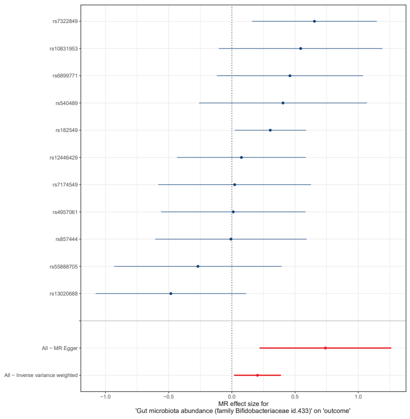
rs7322849
rs10831953
rs6899771
rs540489
rs182549
rs12446429
rs7174549
rs4957061
rs857444
rs55888705
rs13020688
All − MR Egger
All − Inverse variance weighted
−1.0
−0.5
0.0
0.5
1.0
MR effect size for
'Gut microbiota abundance (family Bifidobacteriaceae id.433)' on 'outcome'

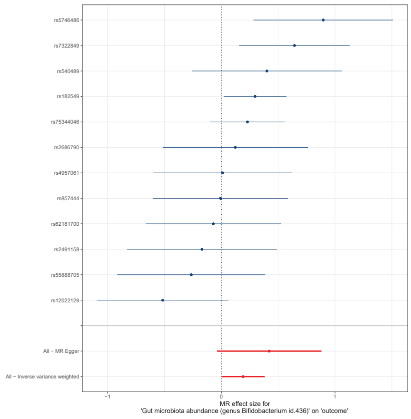
rs5746486
rs7322849
rs540489
rs182549
rs75344046
rs2686790
rs4957061
rs857444
rs62181700
rs2491158
rs55888705
rs12022129
All − MR Egger
All − Inverse variance weighted
−1
0
1
MR effect size for
'Gut microbiota abundance (genus Bifidobacterium id.436)' on 'outcome'

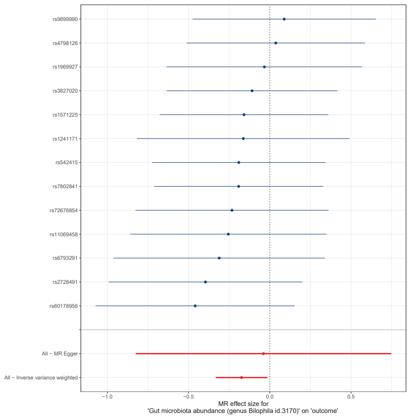
rs9899990
rs4798126
rs1969927
rs3827020
rs1571225
rs1241171
rs542415
rs7802841
rs72676854
rs11069458
rs6793291
rs2728491
rs60178956
All − MR Egger
All − Inverse variance weighted
−1.0
−0.5
0.0
0.5
MR effect size for
'Gut microbiota abundance (genus Bilophila id.3170)' on 'outcome'

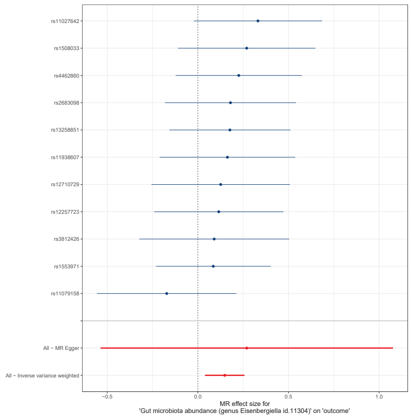
rs11027642
rs1508033
rs4462860
rs2683098
rs13258851
rs11938607
rs12710729
rs12257723
rs3812426
rs1553971
rs11079158
All − MR Egger
All − Inverse variance weighted
−0.5
0.0
0.5
1.0
MR effect size for
'Gut microbiota abundance (genus Eisenbergiella id.11304)' on 'outcome'

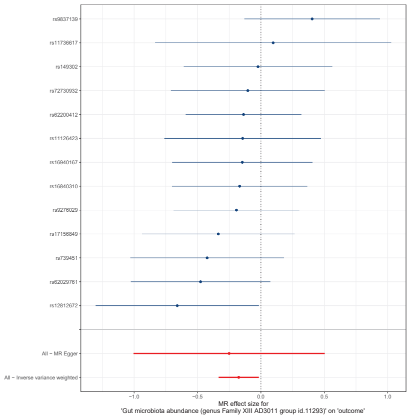
rs9837139
rs11736817
rs149302
rs72730932
rs62200412
rs11126423
rs16940167
rs16840310
rs9276029
rs17156849
rs739451
rs62029781
rs12812672
All − MR Egger
All − Inverse variance weighted
−1.0
−0.5
0.0
0.5
1.0
MR effect size for
'Gut microbiota abundance (genus Family XIII AD3011 group id.11293)' on 'outcome'

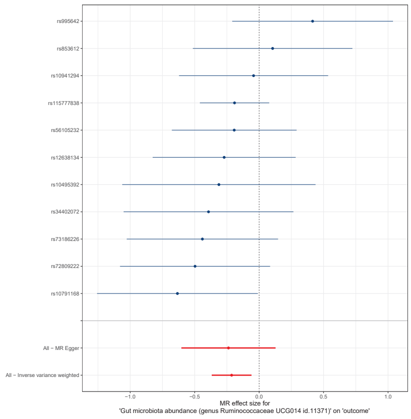
rs995642
rs853612
rs10941294
rs115777838
rs56105232
rs12638134
rs10495392
rs34402072
rs73186226
rs72809222
rs10791168
All − MR Egger
All − Inverse variance weighted
−1.0
−0.5
0.0
0.5
1.0
MR effect size for
'Gut microbiota abundance (genus Ruminococcaceae UCG014 id.11371)' on 'outcome'

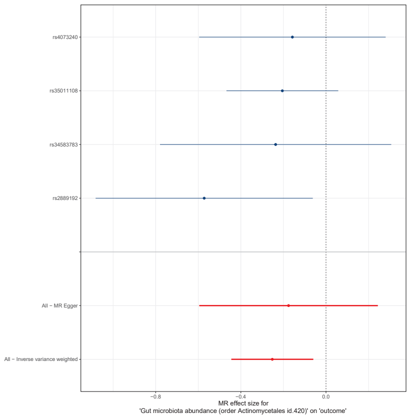
rs4073240
rs35011108
rs34583783
rs2889192
All − MR Egger
All − Inverse variance weighted
−0.8
−0.6
−0.4
−0.2
0.0
MR effect size for
'Gut microbiota abundance (order Actinomycetales id.420)' on 'outcome'

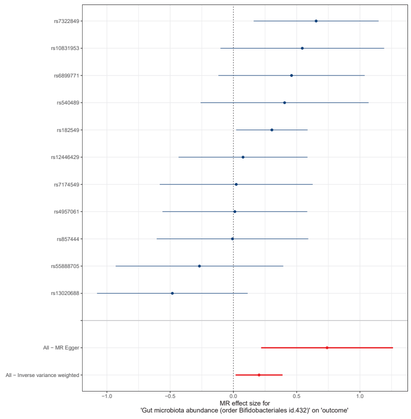
rs7322849
rs10831953
rs6899771
rs540489
rs182549
rs12446429
rs7174549
rs4957061
rs857444
rs55888705
rs13020688
All − MR Egger
All − Inverse variance weighted
−1.0
−0.5
0.0
0.5
1.0
MR effect size for
'Gut microbiota abundance (order Bifidobacteriales id.432)' on 'outcome'

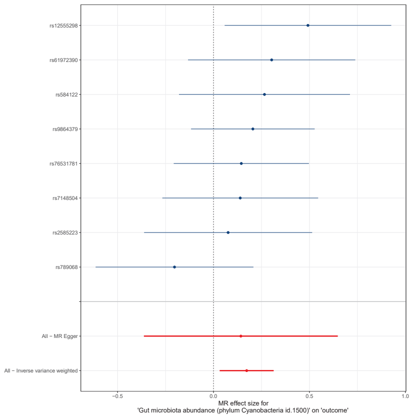
rs12555298
rs61972390
rs584122
rs9864379
rs78631781
rs7148504
rs2585223
rs789068
All − MR Egger
All − Inverse variance weighted
−0.5
0.0
0.5
1.0
MR effect size for
'Gut microbiota abundance (phylum Cyanobacteria id.1500)' on 'outcome'

Supplementary Figure 1. Forest plots of two-sample MR analysis of gut microbiota on osteoporosis.

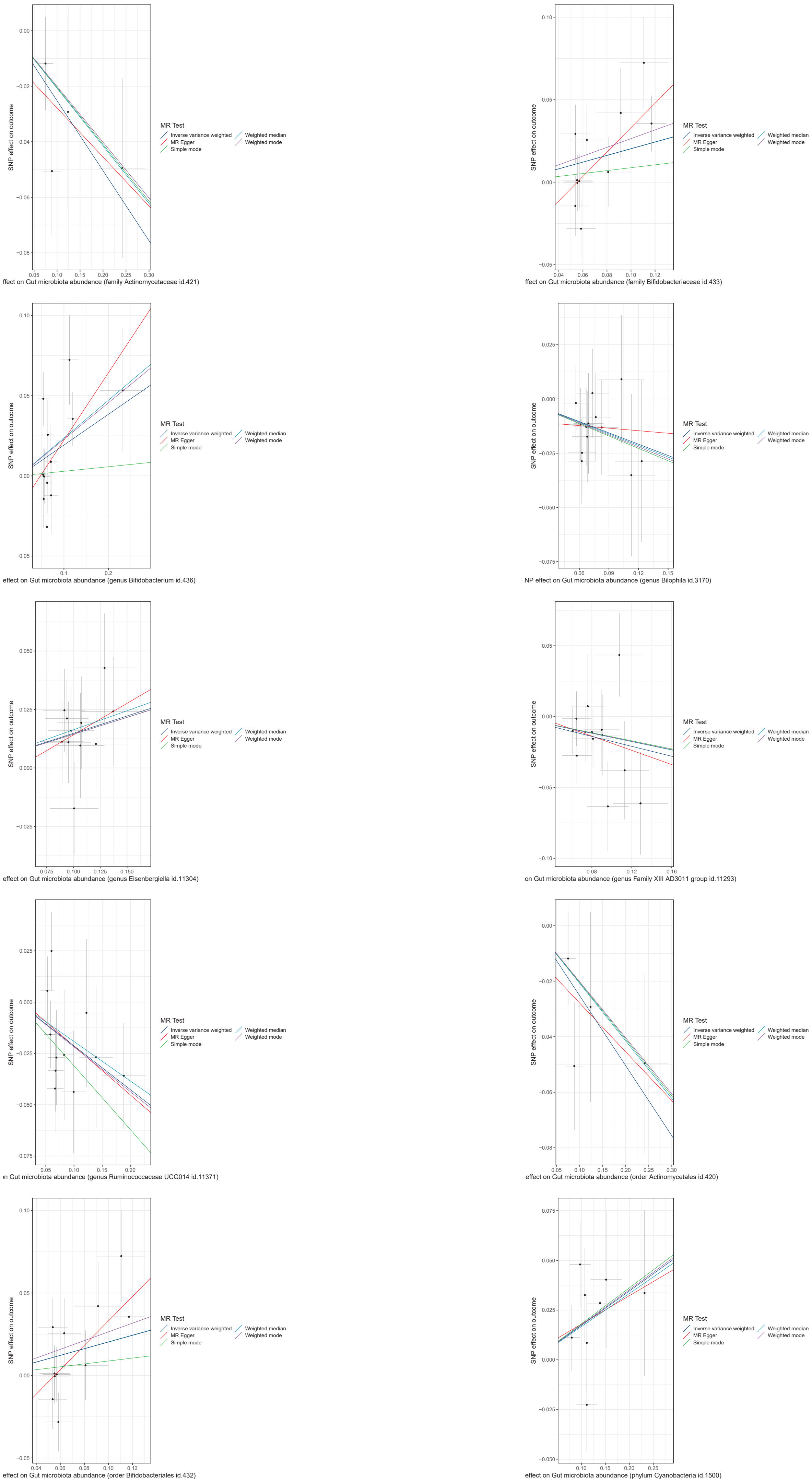
SNP effect on outcome
MR Test
Inverse variance weighted
MR Egger
Simple mode
Weighted median
Weighted mode
ffect on Gut microbiota abundance (family Actinomycetaceae id.421)
ffect on Gut microbiota abundance (family Bifidobacteriaceae id.433)
effect on Gut microbiota abundance (genus Bifidobacterium id.436)
NP effect on Gut microbiota abundance (genus Bilophila id.3170)
effect on Gut microbiota abundance (genus Eisenbergiella id.11304)
on Gut microbiota abundance (genus Family XIII AD3011 group id.11293)
on Gut microbiota abundance (genus Ruminococcaceae UCG014 id.11371)
effect on Gut microbiota abundance (order Actinomycetales id.420)
effect on Gut microbiota abundance (order Bifidobacteriales id.432)
effect on Gut microbiota abundance (phylum Cyanobacteria id.1500)

Supplementary Figure 2. Scatter plots of two-sample MR analysis of gut microbiota on osteoporosis.

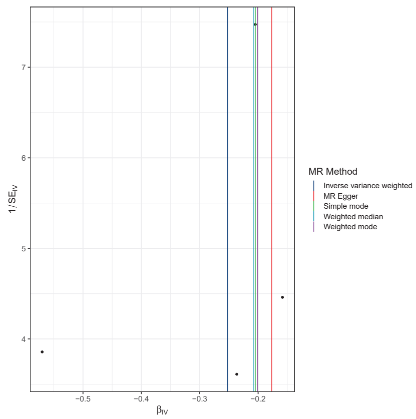
$1/SE_{IV}$
β_{IV}
MR Method
Inverse variance weighted
MR Egger
Simple mode
Weighted median
Weighted mode

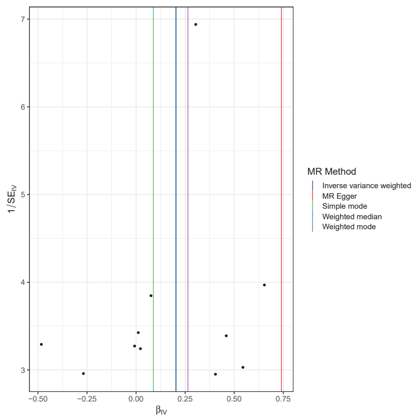
$1/SE_{IV}$
β_{IV}
MR Method
Inverse variance weighted
MR Egger
Simple mode
Weighted median
Weighted mode

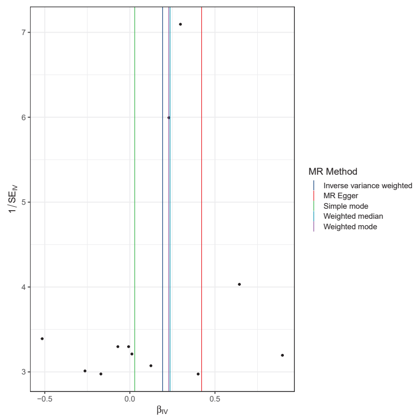
$1/SE_{IV}$
β_{IV}
MR Method
Inverse variance weighted
MR Egger
Simple mode
Weighted median
Weighted mode

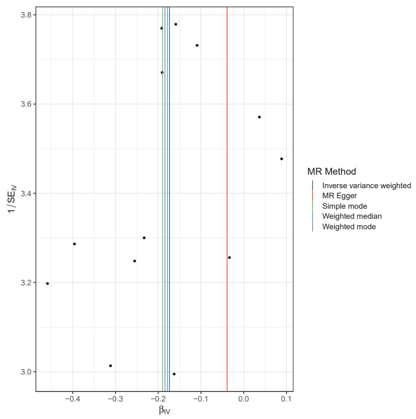
$1/SE_{IV}$
β_{IV}
MR Method
Inverse variance weighted
MR Egger
Simple mode
Weighted median
Weighted mode

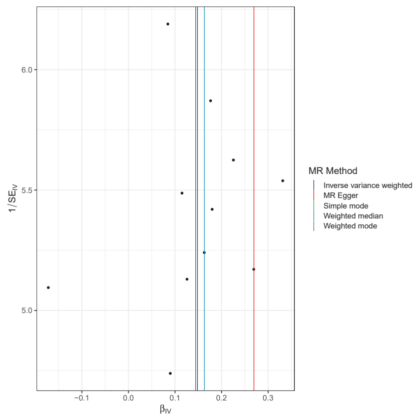
$1/SE_{IV}$
β_{IV}
MR Method
Inverse variance weighted
MR Egger
Simple mode
Weighted median
Weighted mode

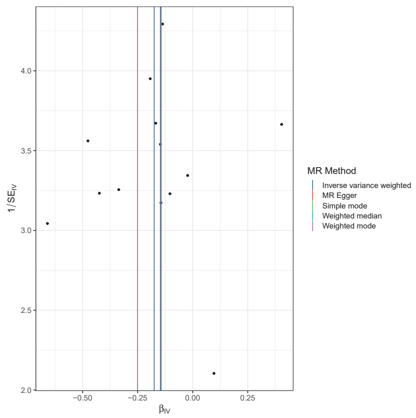
$1/SE_{IV}$
β_{IV}
MR Method
Inverse variance weighted
MR Egger
Simple mode
Weighted median
Weighted mode

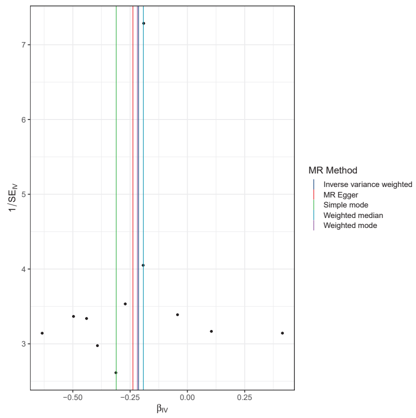
$1/SE_{IV}$
β_{IV}
MR Method
Inverse variance weighted
MR Egger
Simple mode
Weighted median
Weighted mode

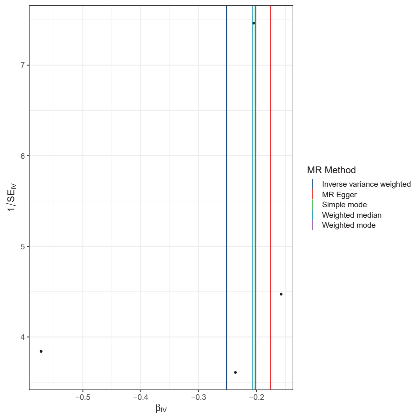
$1/SE_{IV}$
β_{IV}
MR Method
Inverse variance weighted
MR Egger
Simple mode
Weighted median
Weighted mode

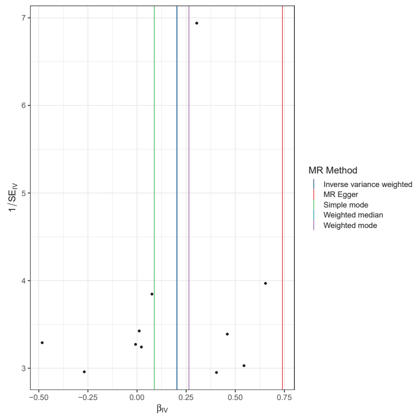
$1/SE_{IV}$
β_{IV}
MR Method
Inverse variance weighted
MR Egger
Simple mode
Weighted median
Weighted mode

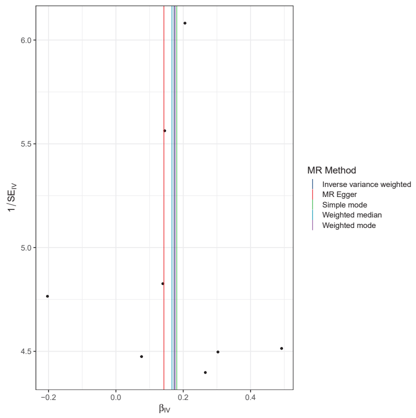
$1/SE_{IV}$
β_{IV}
MR Method
Inverse variance weighted
MR Egger
Simple mode
Weighted median
Weighted mode

Supplementary Figure 3. Funnel plots of two-sample MR analysis of gut microbiota on osteoporosis.

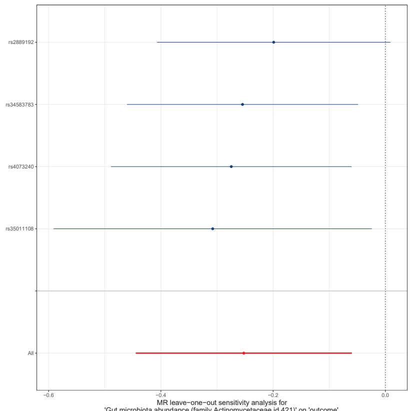

MR leave−one−out sensitivity analysis for
'Gut microbiota abundance (family Actinomycetaceae id.421)' on 'outcome'

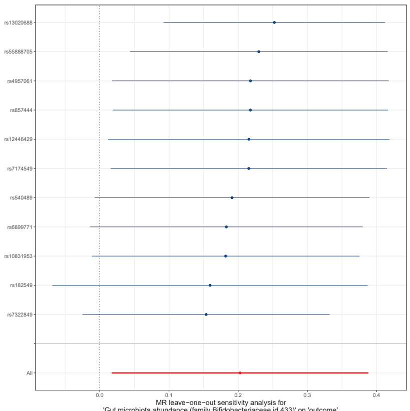

MR leave−one−out sensitivity analysis for
'Gut microbiota abundance (family Bifidobacteriaceae id.433)' on 'outcome'

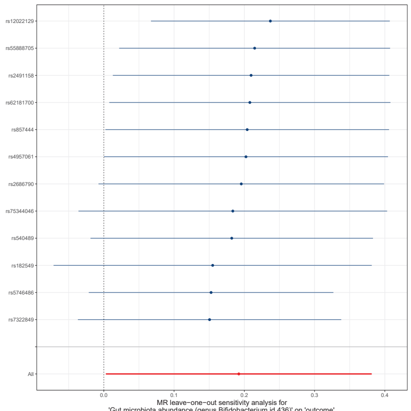

MR leave−one−out sensitivity analysis for
'Gut microbiota abundance (genus Bifidobacterium id.436)' on 'outcome'

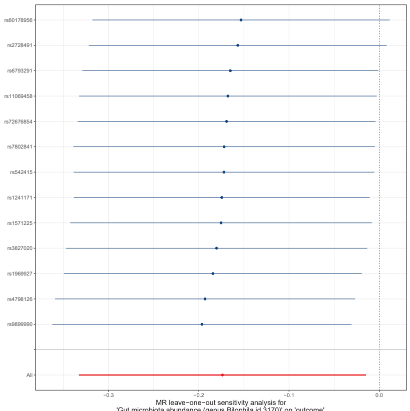

MR leave−one−out sensitivity analysis for
'Gut microbiota abundance (genus Bilophila id.3170)' on 'outcome'

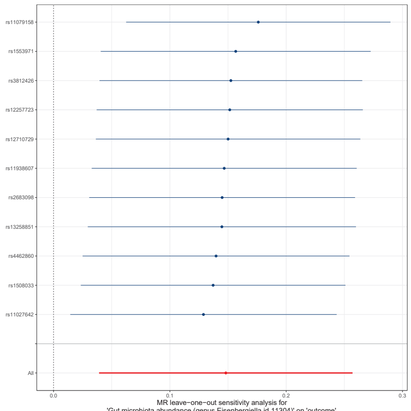

MR leave−one−out sensitivity analysis for
'Gut microbiota abundance (genus Eisenbergiella id.11304)' on 'outcome'

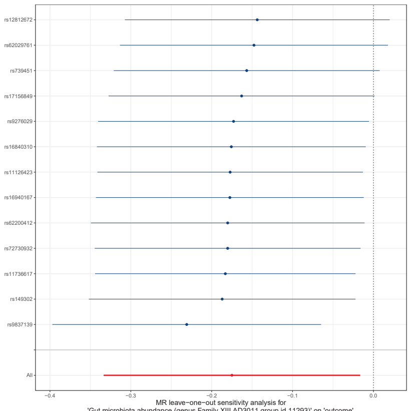

MR leave−one−out sensitivity analysis for
'Gut microbiota abundance (genus Family XIII AD3011 group id.11293)' on 'outcome'

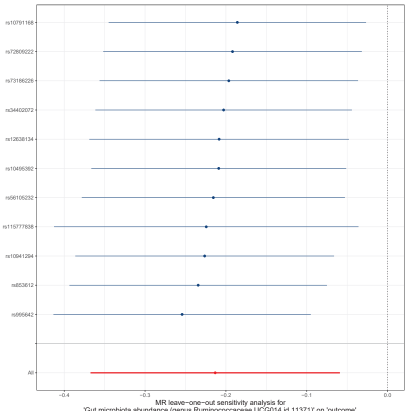

MR leave−one−out sensitivity analysis for
'Gut microbiota abundance (genus Ruminococcaceae UCG014 id.11371)' on 'outcome'

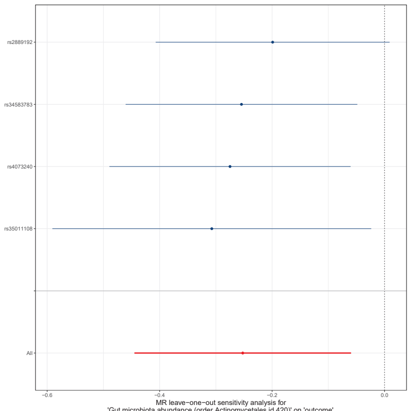

MR leave−one−out sensitivity analysis for
'Gut microbiota abundance (order Actinomycetales id.420)' on 'outcome'

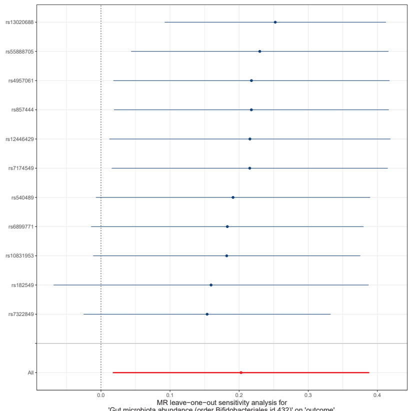

MR leave−one−out sensitivity analysis for
'Gut microbiota abundance (order Bifidobacteriales id.432)' on 'outcome'

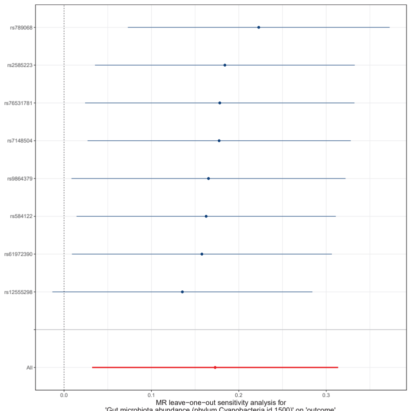

MR leave−one−out sensitivity analysis for
'Gut microbiota abundance (phylum Cyanobacteria id.1500)' on 'outcome'

Supplementary Figure 4. Leave-one-out plots of two-sample MR analysis of gut microbiota on osteoporosis.

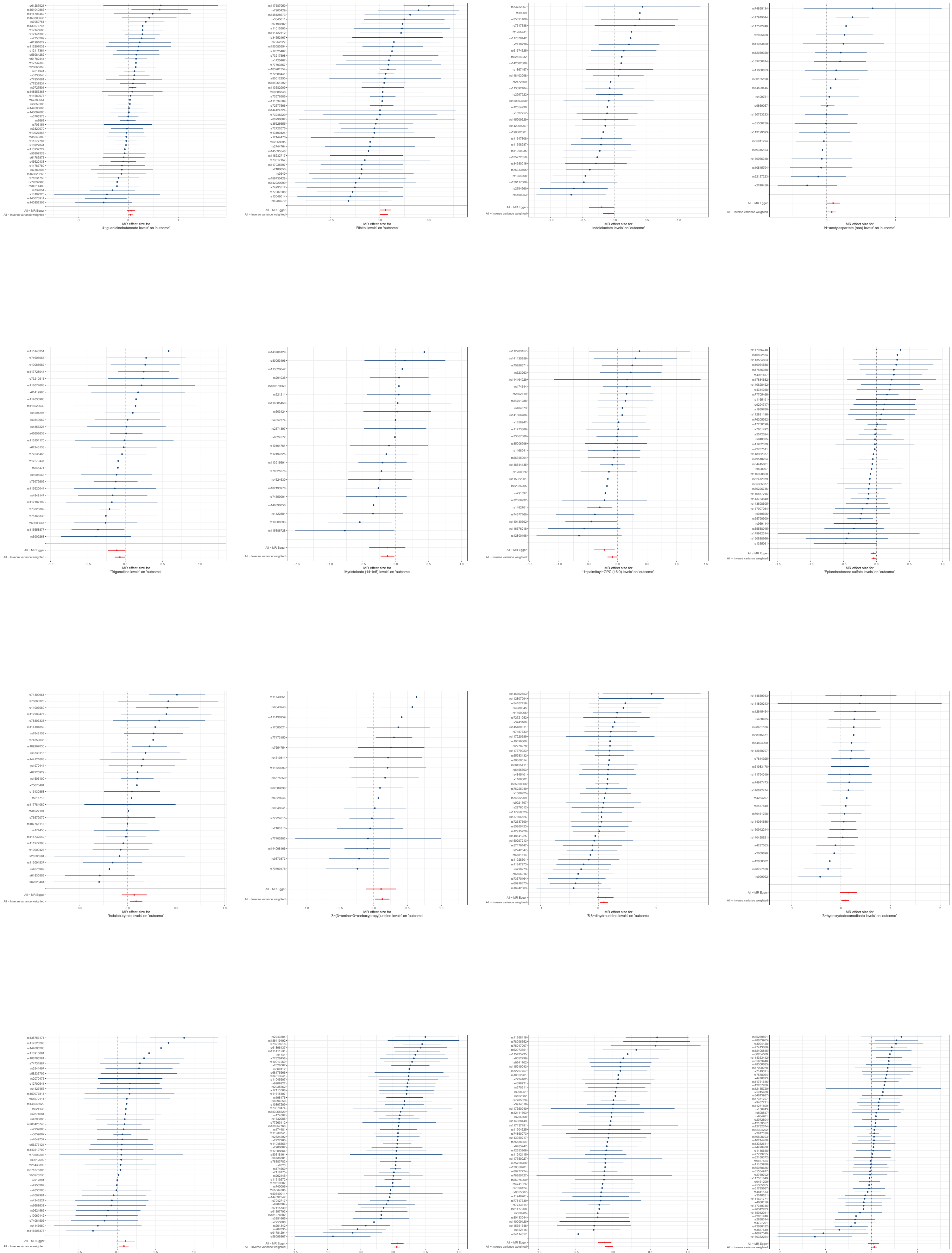

MR effect size for '4-guanidinobutanoate levels' on 'outcome'
MR effect size for 'Ribitol levels' on 'outcome'
MR effect size for 'Indolelactate levels' on 'outcome'
MR effect size for 'N-acetylaspartate (naa) levels' on 'outcome'
MR effect size for 'Trigonelline levels' on 'outcome'
MR effect size for 'Myristoleate (14:1n5) levels' on 'outcome'
MR effect size for '1-palmitoyl-GPC (16:0) levels' on 'outcome'
MR effect size for 'Epiandrosterone sulfate levels' on 'outcome'
MR effect size for 'Indolebutyrate levels' on 'outcome'
MR effect size for '3-(3-amino-3-carboxypropyl)uridine levels' on 'outcome'
MR effect size for '5,6-dihydrouridine levels' on 'outcome'
MR effect size for '3-hydroxydodecanedioate levels' on 'outcome'
MR effect size for 'Cysteine-glutathione disulfide levels' on 'outcome'
MR effect size for '1-arachidonoyl-GPE (20:4n6) levels' on 'outcome'
MR effect size for 'Tryptophan betaine levels' on 'outcome'
MR effect size for 'Hexanoylglutamine levels' on 'outcome'
All – MR Egger
All – Inverse variance weighted

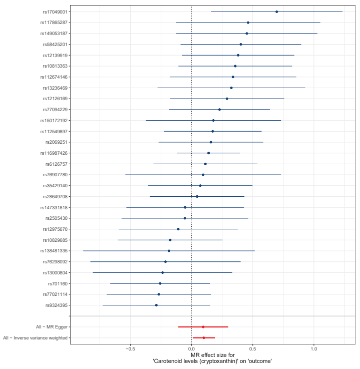
All − MR Egger
All − Inverse variance weighted
MR effect size for
'Carotenoid levels (cryptoxanthin)' on 'outcome'

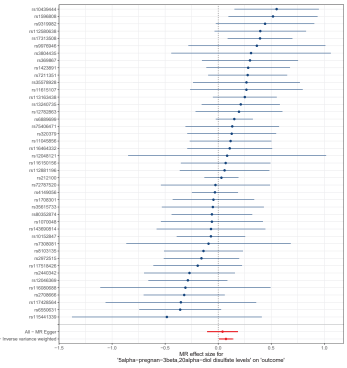
All − MR Egger
All − Inverse variance weighted
MR effect size for
'5alpha−pregnan−3beta,20alpha−diol disulfate levels' on 'outcome'

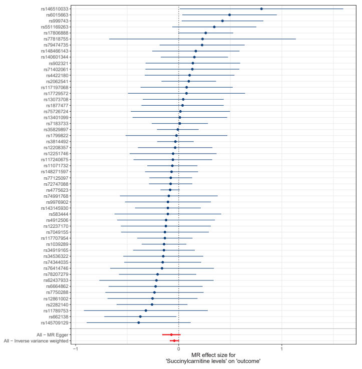
All − MR Egger
All − Inverse variance weighted
MR effect size for
'Succinylcarnitine levels' on 'outcome'

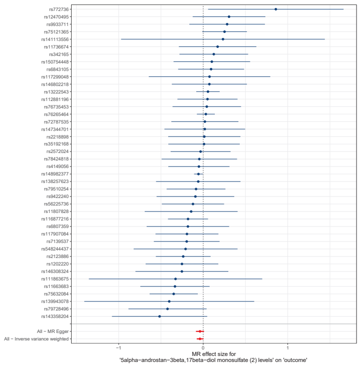
All − MR Egger
All − Inverse variance weighted
MR effect size for
'5alpha−androstan−3beta,17beta−diol monosulfate (2) levels' on 'outcome'

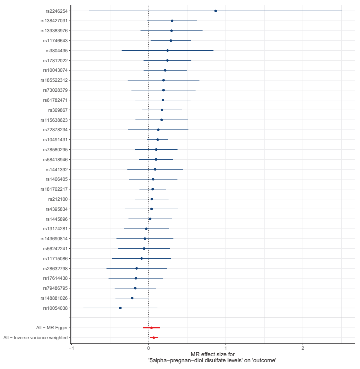
All − MR Egger
All − Inverse variance weighted
MR effect size for
'5alpha−pregnan−diol disulfate levels' on 'outcome'

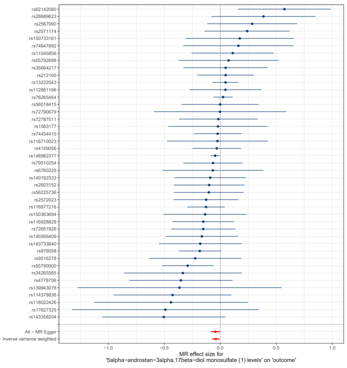
All − MR Egger
All − Inverse variance weighted
MR effect size for
'5alpha−androstan−3alpha,17beta−diol monosulfate (1) levels' on 'outcome'

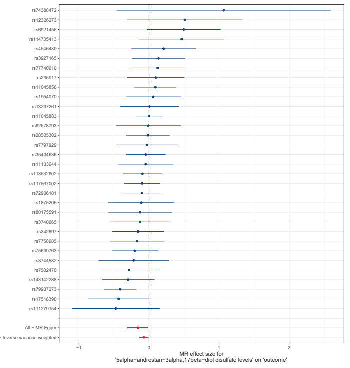
All − MR Egger
All − Inverse variance weighted
MR effect size for
'5alpha−androstan−3alpha,17beta−diol disulfate levels' on 'outcome'

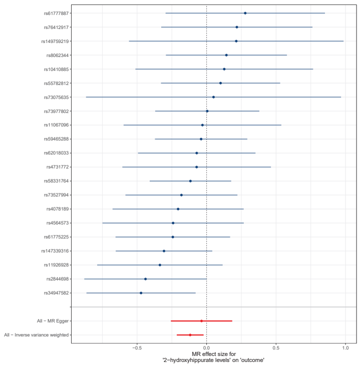
All − MR Egger
All − Inverse variance weighted
MR effect size for
'2−hydroxyhippurate levels' on 'outcome'

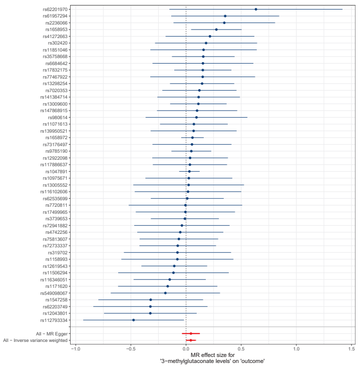
All − MR Egger
All − Inverse variance weighted
MR effect size for
'3−methylglutaconate levels' on 'outcome'

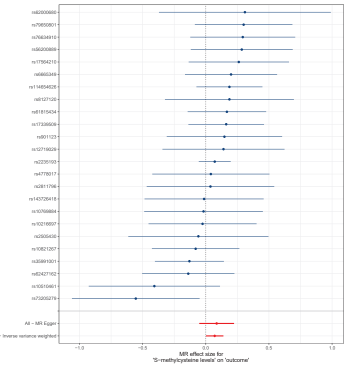
All − MR Egger
All − Inverse variance weighted
MR effect size for
'S−methylcysteine levels' on 'outcome'

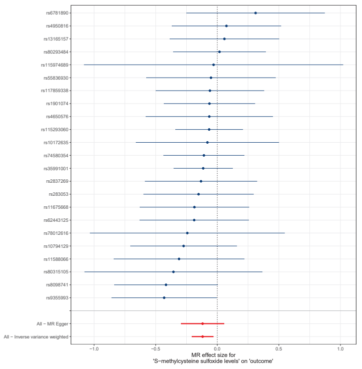
All − MR Egger
All − Inverse variance weighted
MR effect size for
'S−methylcysteine sulfoxide levels' on 'outcome'

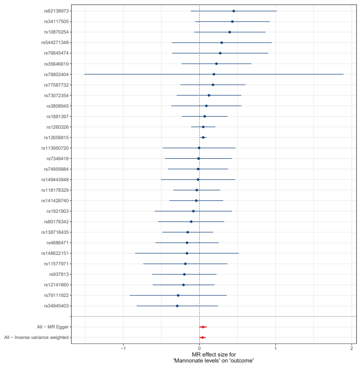
All − MR Egger
All − Inverse variance weighted
MR effect size for
'Mannonate levels' on 'outcome'

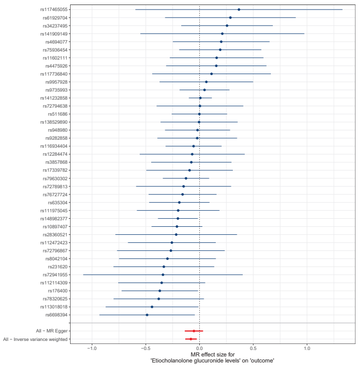
All − MR Egger
All − Inverse variance weighted
MR effect size for
'Etiocholanolone glucuronide levels' on 'outcome'

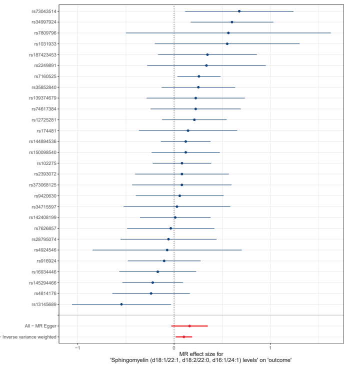
All − MR Egger
All − Inverse variance weighted
MR effect size for
'Sphingomyelin (d18:1/22:1, d18:2/22:0, d16:1/24:1) levels' on 'outcome'

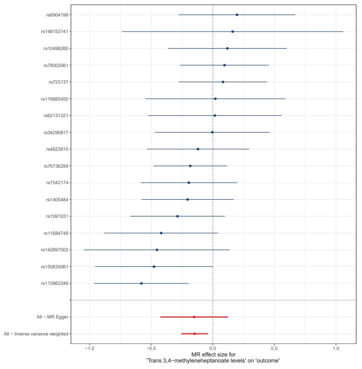
All − MR Egger
All − Inverse variance weighted
MR effect size for
'Trans 3,4−methyleneheptanoate levels' on 'outcome'

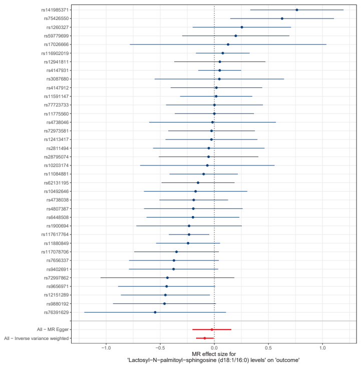
All − MR Egger
All − Inverse variance weighted
MR effect size for
'Lactosyl−N−palmitoyl−sphingosine (d18:1/16:0) levels' on 'outcome'

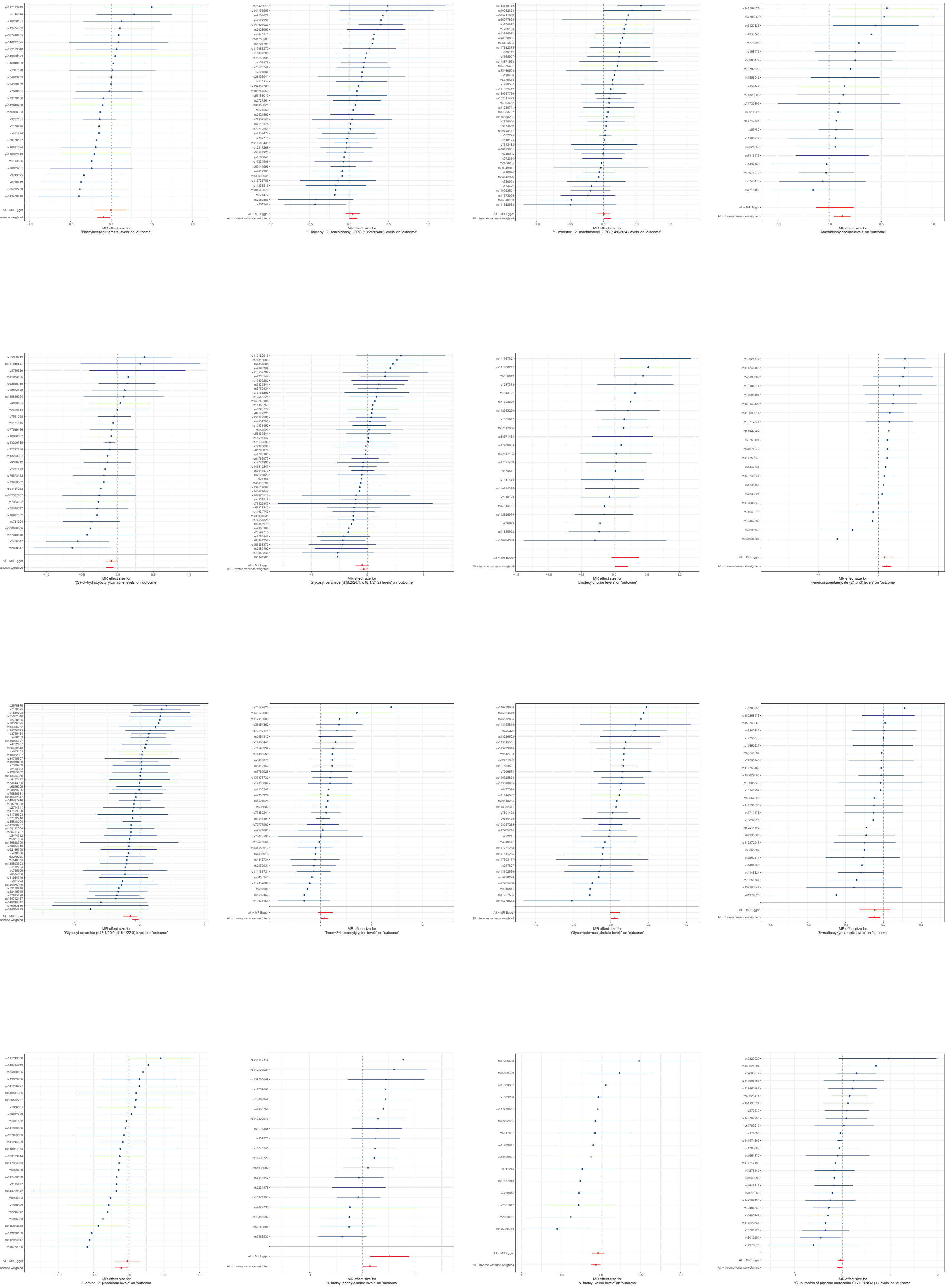
All − MR Egger
All − Inverse variance weighted
MR effect size for 'Phenylacetylglutamate levels' on 'outcome'
MR effect size for '1−linoleoyl−2−arachidonoyl−GPC (18:2/20:4n6) levels' on 'outcome'
MR effect size for '1−myristoyl−2−arachidonoyl−GPC (14:0/20:4) levels' on 'outcome'
MR effect size for 'Arachidonoylcholine levels' on 'outcome'
MR effect size for '(S)−3−hydroxybutyrylcarnitine levels' on 'outcome'
MR effect size for 'Glycosyl ceramide (d18:2/24:1, d18:1/24:2) levels' on 'outcome'
MR effect size for 'Linoleoylcholine levels' on 'outcome'
MR effect size for 'Heneicosapentaenoate (21:5n3) levels' on 'outcome'
MR effect size for 'Glycosyl ceramide (d18:1/20:0, d16:1/22:0) levels' on 'outcome'
MR effect size for 'Trans−2−hexenoylglycine levels' on 'outcome'
MR effect size for 'Glyco−beta−muricholate levels' on 'outcome'
MR effect size for '8−methoxykynurenate levels' on 'outcome'
MR effect size for '3−amino−2−piperidone levels' on 'outcome'
MR effect size for 'N−lactoyl phenylalanine levels' on 'outcome'
MR effect size for 'N−lactoyl valine levels' on 'outcome'
MR effect size for 'Glucuronide of piperine metabolite C17H21NO3 (4) levels' on 'outcome'

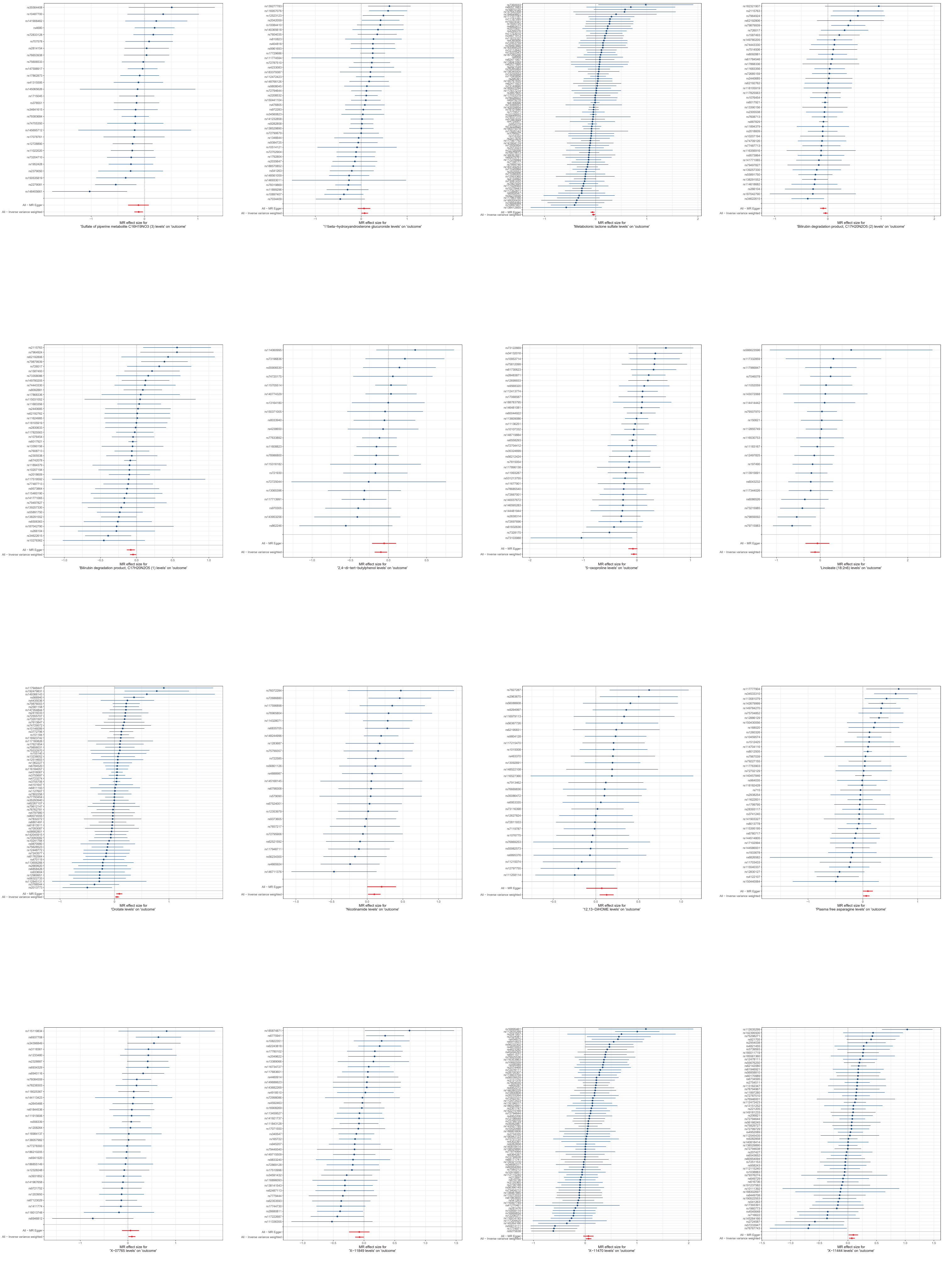
All − MR Egger
All − Inverse variance weighted
MR effect size for
'Sulfate of piperine metabolite C16H19NO3 (3) levels' on 'outcome'
MR effect size for
'11beta-hydroxyandrosterone glucuronide levels' on 'outcome'
MR effect size for
'Metabolonic lactone sulfate levels' on 'outcome'
MR effect size for
'Bilirubin degradation product, C17H20N2O5 (2) levels' on 'outcome'
MR effect size for
'Bilirubin degradation product, C17H20N2O5 (1) levels' on 'outcome'
MR effect size for
'2,4−di−tert−butylphenol levels' on 'outcome'
MR effect size for
'5−oxoproline levels' on 'outcome'
MR effect size for
'Linoleate (18:2n6) levels' on 'outcome'
MR effect size for
'Orotate levels' on 'outcome'
MR effect size for
'Nicotinamide levels' on 'outcome'
MR effect size for
'12,13−DiHOME levels' on 'outcome'
MR effect size for
'Plasma free asparagine levels' on 'outcome'
MR effect size for
'X−07765 levels' on 'outcome'
MR effect size for
'X−11849 levels' on 'outcome'
MR effect size for
'X−11470 levels' on 'outcome'
MR effect size for
'X−11444 levels' on 'outcome'

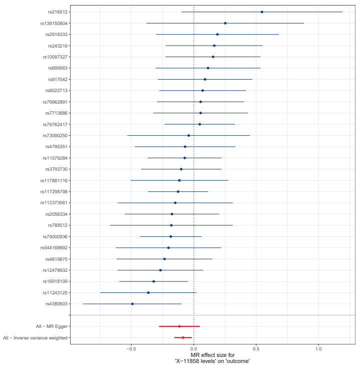
All − MR Egger
All − Inverse variance weighted
MR effect size for
'X−11858 levels' on 'outcome'

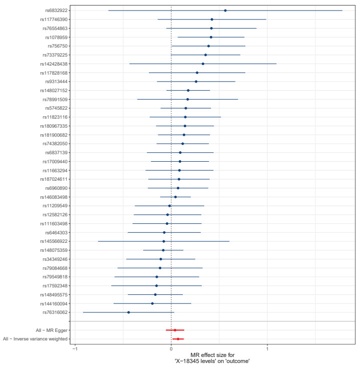
All − MR Egger
All − Inverse variance weighted
MR effect size for
'X−18345 levels' on 'outcome'

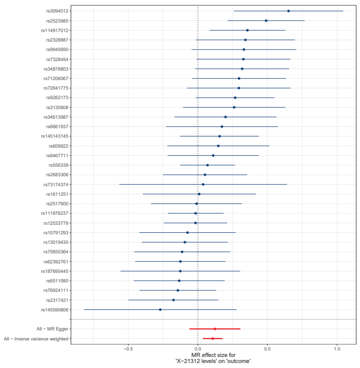
All − MR Egger
All − Inverse variance weighted
MR effect size for
'X−21312 levels' on 'outcome'

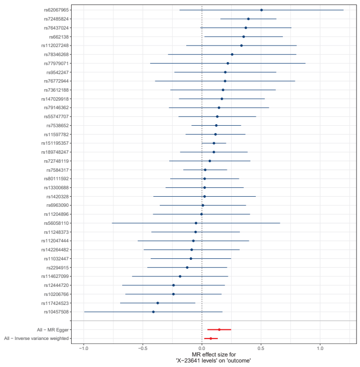
All − MR Egger
All − Inverse variance weighted
MR effect size for
'X−23641 levels' on 'outcome'

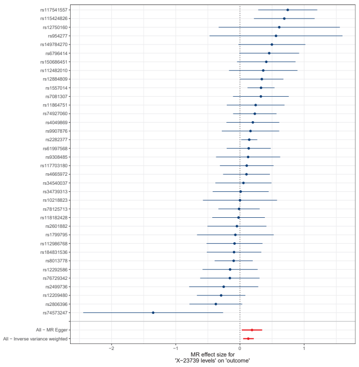
All − MR Egger
All − Inverse variance weighted
MR effect size for
'X−23739 levels' on 'outcome'

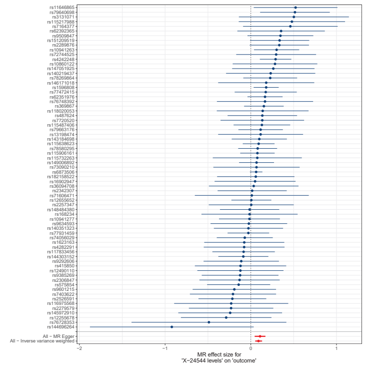
All − MR Egger
All − Inverse variance weighted
MR effect size for
'X−24544 levels' on 'outcome'

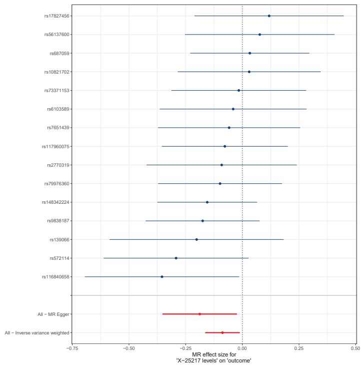
All − MR Egger
All − Inverse variance weighted
MR effect size for
'X−25217 levels' on 'outcome'

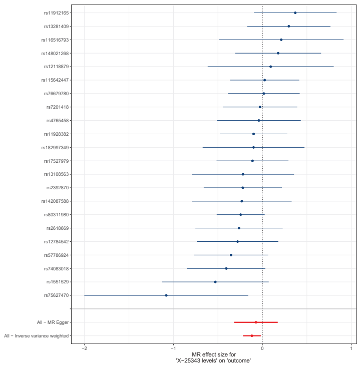
All − MR Egger
All − Inverse variance weighted
MR effect size for
'X−25343 levels' on 'outcome'

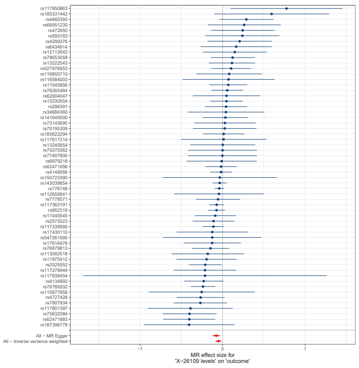
All − MR Egger
All − Inverse variance weighted
MR effect size for
'X−26109 levels' on 'outcome'

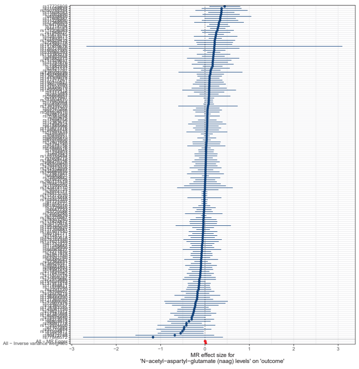
MR effect size for
'N−acetyl−aspartyl−glutamate (naag) levels' on 'outcome'

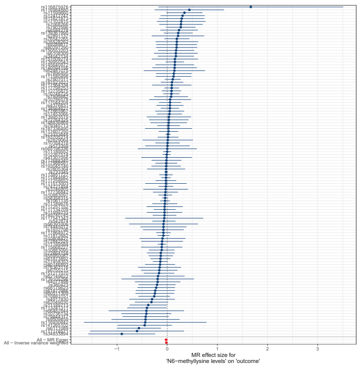
MR effect size for
'N6−methyllysine levels' on 'outcome'

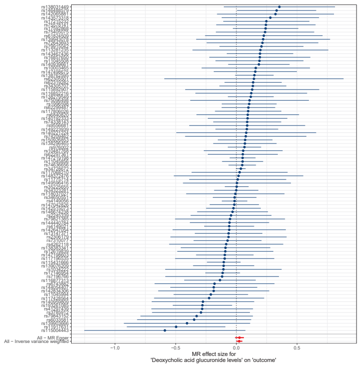
MR effect size for
'Deoxycholic acid glucuronide levels' on 'outcome'

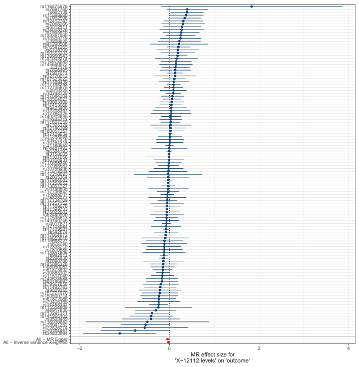
MR effect size for
'X−12112 levels' on 'outcome'

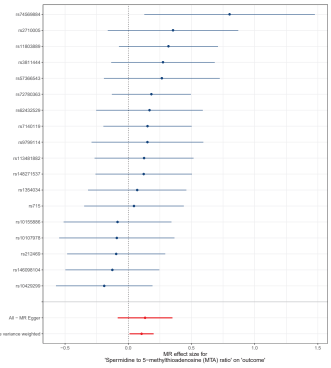
All − MR Egger
All − Inverse variance weighted
MR effect size for
'Spermidine to 5−methylthioadenosine (MTA) ratio' on 'outcome'

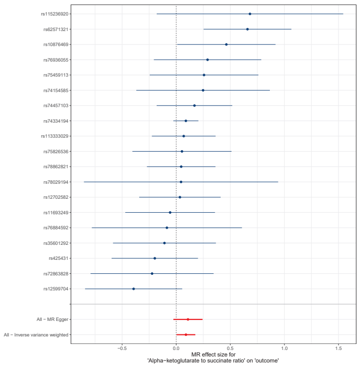
All − MR Egger
All − Inverse variance weighted
MR effect size for
'Alpha−ketoglutarate to succinate ratio' on 'outcome'

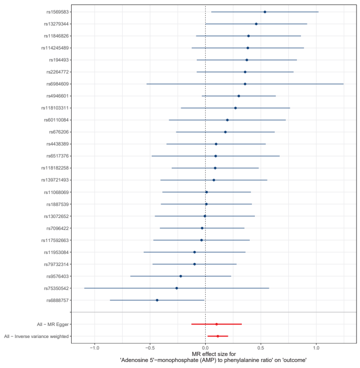
All − MR Egger
All − Inverse variance weighted
MR effect size for
'Adenosine 5'−monophosphate (AMP) to phenylalanine ratio' on 'outcome'

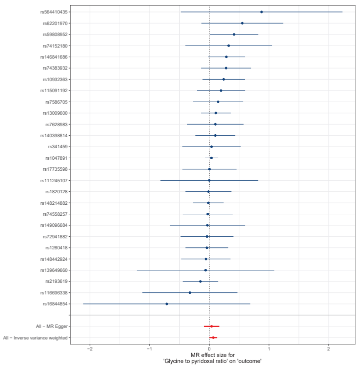
MR effect size for
'Glycine to pyridoxal ratio' on 'outcome'

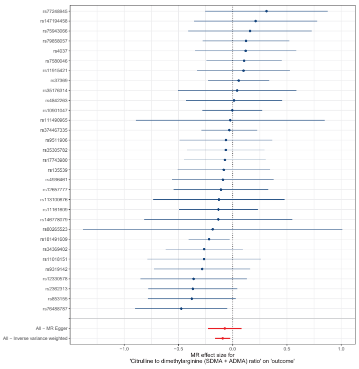
MR effect size for
'Citrulline to dimethylarginine (SDMA + ADMA) ratio' on 'outcome'

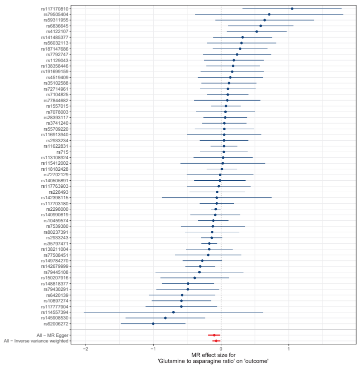
MR effect size for
'Glutamine to asparagine ratio' on 'outcome'

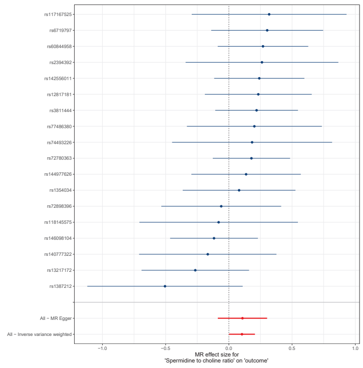
MR effect size for
'Spermidine to choline ratio' on 'outcome'

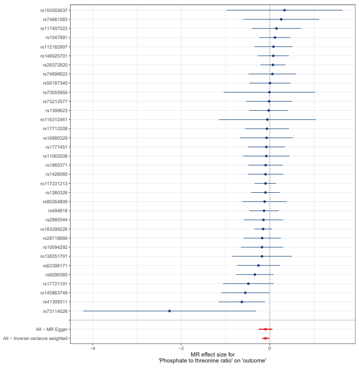
MR effect size for
'Phosphate to threonine ratio' on 'outcome'

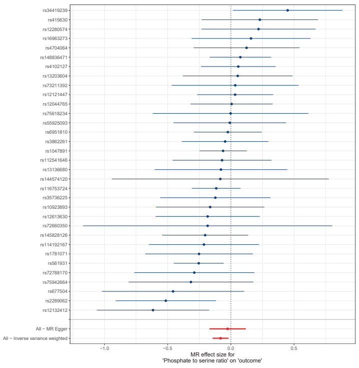
MR effect size for
'Phosphate to serine ratio' on 'outcome'

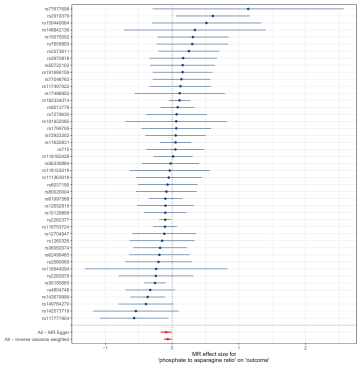
MR effect size for
'phosphate to asparagine ratio' on 'outcome'

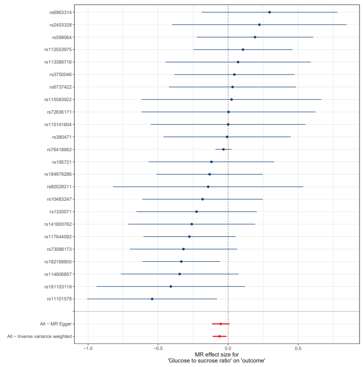
MR effect size for
'Glucose to sucrose ratio' on 'outcome'

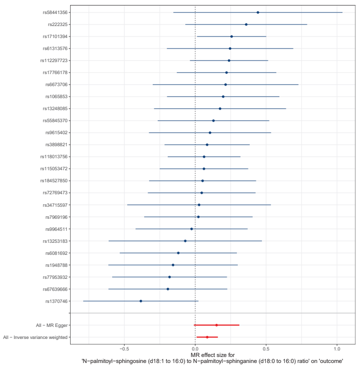
MR effect size for
'N−palmitoyl−sphingosine (d18:1 to 16:0) to N−palmitoyl−sphinganine (d18:0 to 16:0) ratio' on 'outcome'

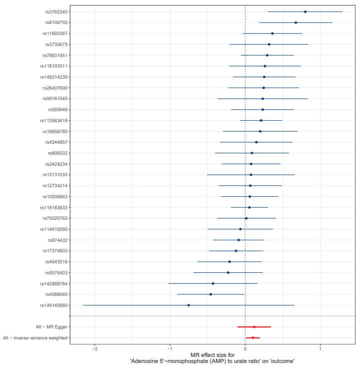
MR effect size for
'Adenosine 5'−monophosphate (AMP) to urate ratio' on 'outcome'

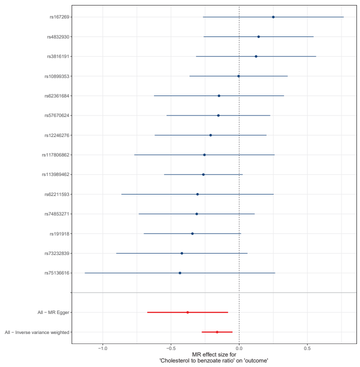
MR effect size for
'Cholesterol to benzoate ratio' on 'outcome'

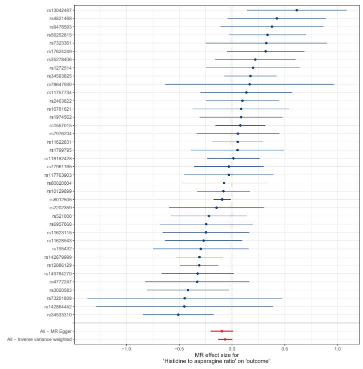
MR effect size for
'Histidine to asparagine ratio' on 'outcome'

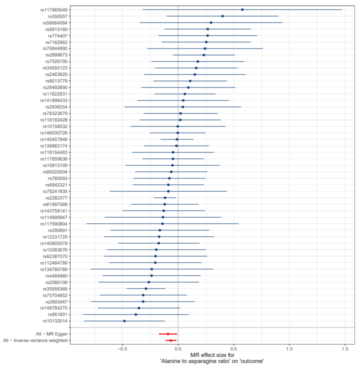
MR effect size for
'Alanine to asparagine ratio' on 'outcome'

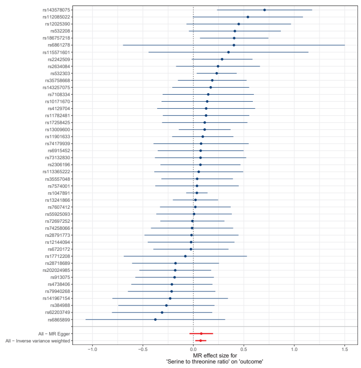
MR effect size for
'Serine to threonine ratio' on 'outcome'

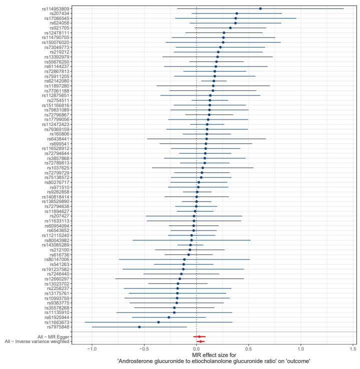
MR effect size for
'Androsterone glucuronide to etiocholanolone glucuronide ratio' on 'outcome'

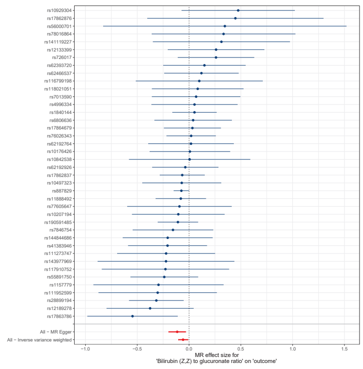
MR effect size for
'Bilirubin (Z,Z) to glucuronate ratio' on 'outcome'

Supplementary Figure 5. Forest plots of two-sample MR analysis of plasma metabolites on osteoporosis.

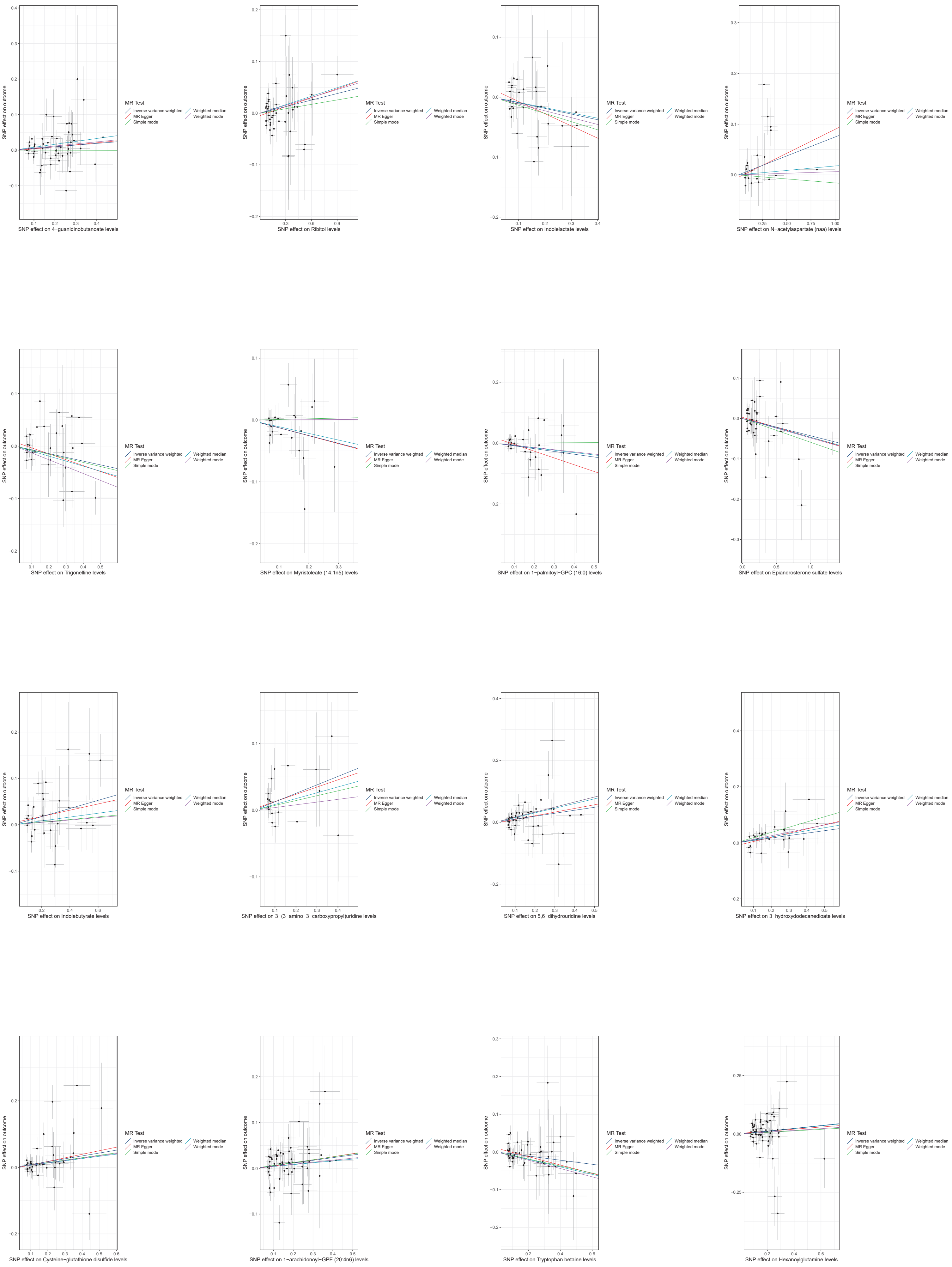
SNP effect on outcome
SNP effect on 4−guanidinobutanoate levels
SNP effect on Ribitol levels
SNP effect on Indolelactate levels
SNP effect on N−acetylaspartate (naa) levels
SNP effect on Trigonelline levels
SNP effect on Myristoleate (14:1n5) levels
SNP effect on 1−palmitoyl−GPC (16:0) levels
SNP effect on Epiandrosterone sulfate levels
SNP effect on Indolebutyrate levels
SNP effect on 3−(3−amino−3−carboxypropyl)uridine levels
SNP effect on 5,6−dihydrouridine levels
SNP effect on 3−hydroxydodecanedioate levels
SNP effect on Cysteine−glutathione disulfide levels
SNP effect on 1−arachidonoyl−GPE (20:4n6) levels
SNP effect on Tryptophan betaine levels
SNP effect on Hexanoylglutamine levels
MR Test
Inverse variance weighted
MR Egger
Simple mode
Weighted median
Weighted mode

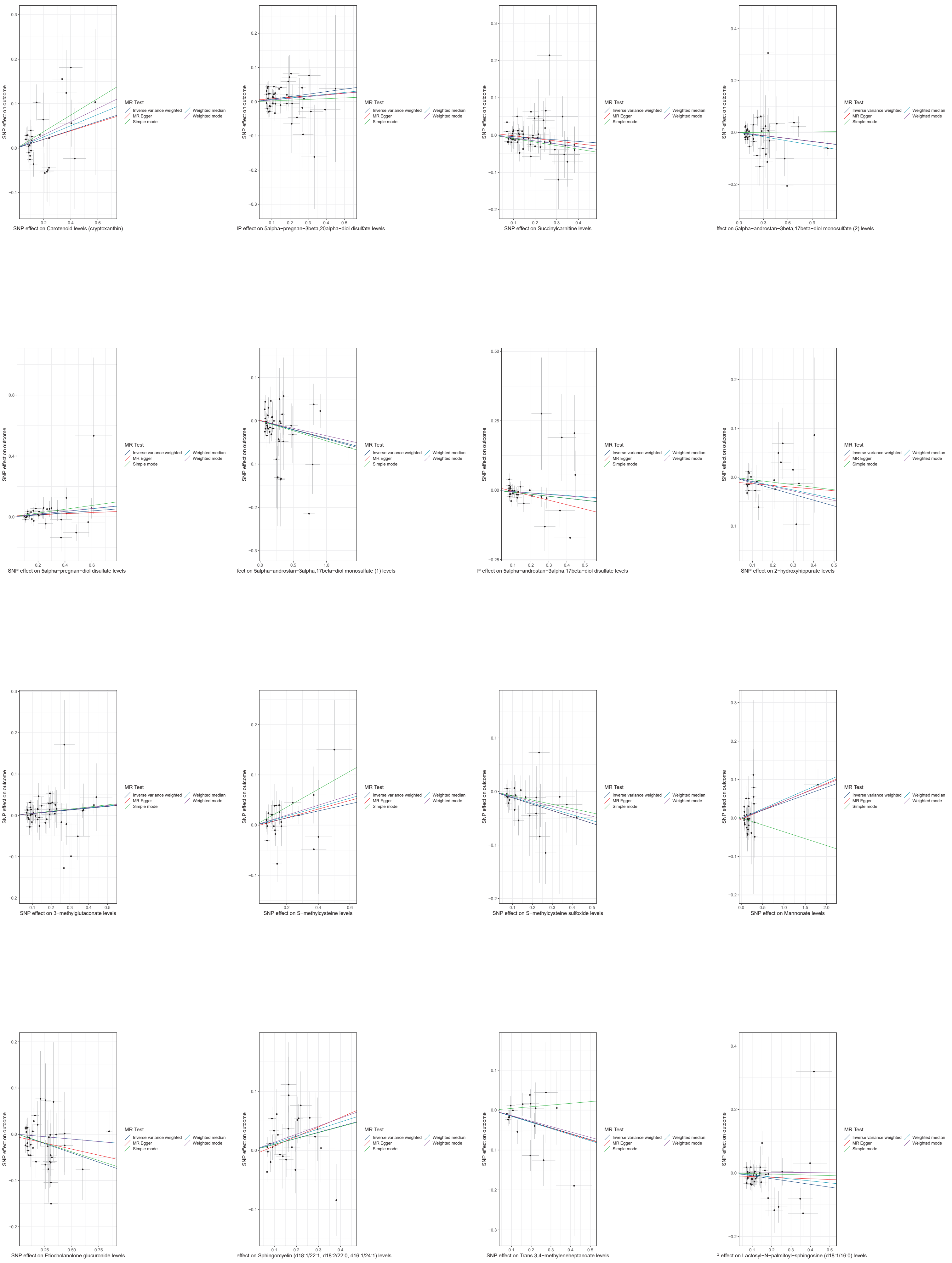
SNP effect on outcome
MR Test
Inverse variance weighted
MR Egger
Simple mode
Weighted median
Weighted mode
SNP effect on Carotenoid levels (cryptoxanthin)
IP effect on 5alpha−pregnan−3beta,20alpha−diol disulfate levels
SNP effect on Succinylcarnitine levels
ffect on 5alpha−androstan−3beta,17beta−diol monosulfate (2) levels
SNP effect on 5alpha−pregnan−diol disulfate levels
fect on 5alpha−androstan−3alpha,17beta−diol monosulfate (1) levels
P effect on 5alpha−androstan−3alpha,17beta−diol disulfate levels
SNP effect on 2−hydroxyhippurate levels
SNP effect on 3−methylglutaconate levels
SNP effect on S−methylcysteine levels
SNP effect on S−methylcysteine sulfoxide levels
SNP effect on Mannonate levels
SNP effect on Etiocholanolone glucuronide levels
effect on Sphingomyelin (d18:1/22:1, d18:2/22:0, d16:1/24:1) levels
SNP effect on Trans 3,4−methyleneheptanoate levels
effect on Lactosyl−N−palmitoyl−sphingosine (d18:1/16:0) levels

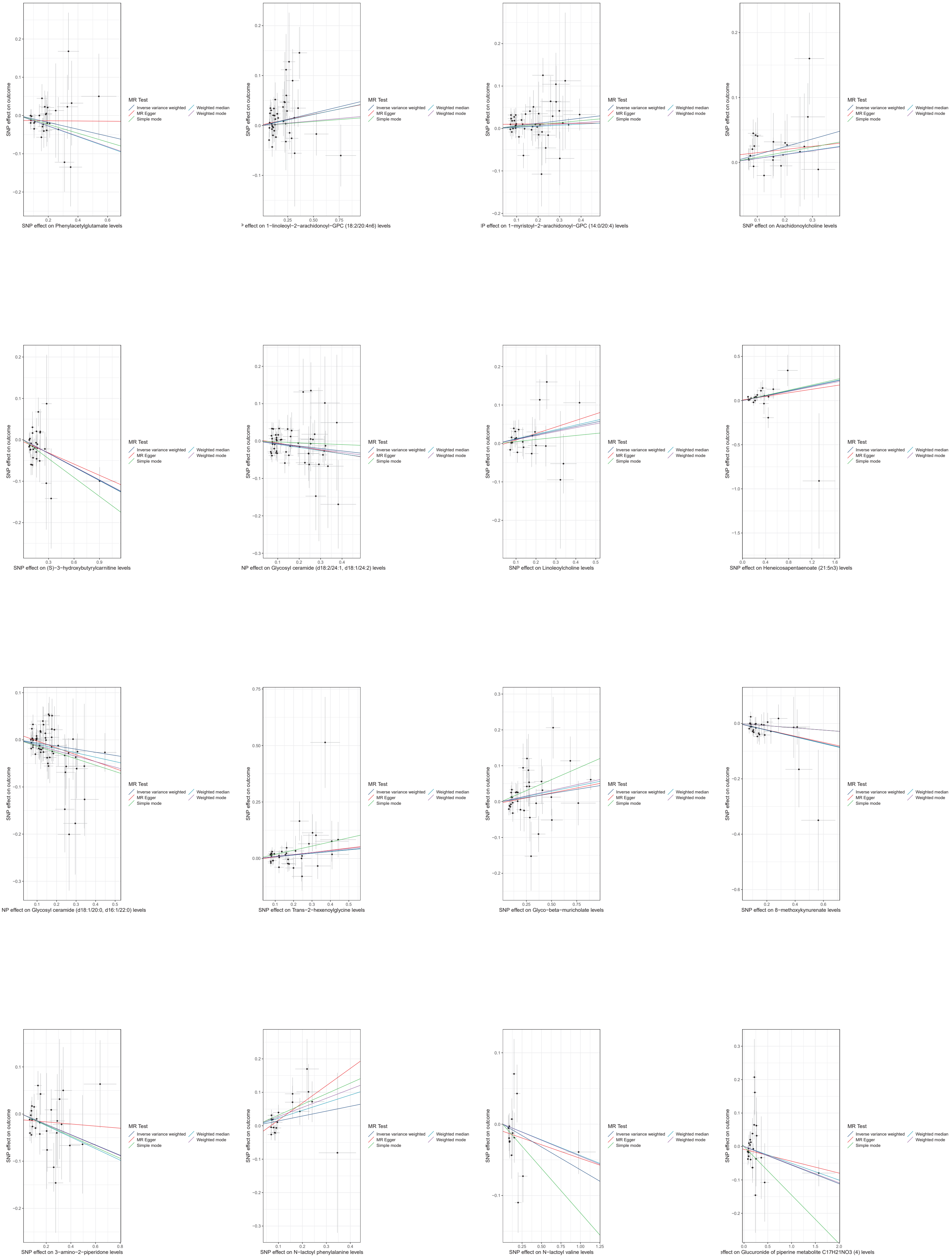
SNP effect on outcome
MR Test
Inverse variance weighted
MR Egger
Simple mode
Weighted median
Weighted mode
SNP effect on Phenylacetylglutamate levels
P effect on 1−linoleoyl−2−arachidonoyl−GPC (18:2/20:4n6) levels
IP effect on 1−myristoyl−2−arachidonoyl−GPC (14:0/20:4) levels
SNP effect on Arachidonoylcholine levels
SNP effect on (S)−3−hydroxybutyrylcarnitine levels
NP effect on Glycosyl ceramide (d18:2/24:1, d18:1/24:2) levels
SNP effect on Linoleoylcholine levels
SNP effect on Heneicosapentaenoate (21:5n3) levels
NP effect on Glycosyl ceramide (d18:1/20:0, d16:1/22:0) levels
SNP effect on Trans−2−hexenoylglycine levels
SNP effect on Glyco−beta−muricholate levels
SNP effect on 8−methoxykynurenate levels
SNP effect on 3−amino−2−piperidone levels
SNP effect on N−lactoyl phenylalanine levels
SNP effect on N−lactoyl valine levels
effect on Glucuronide of piperine metabolite C17H21NO3 (4) levels

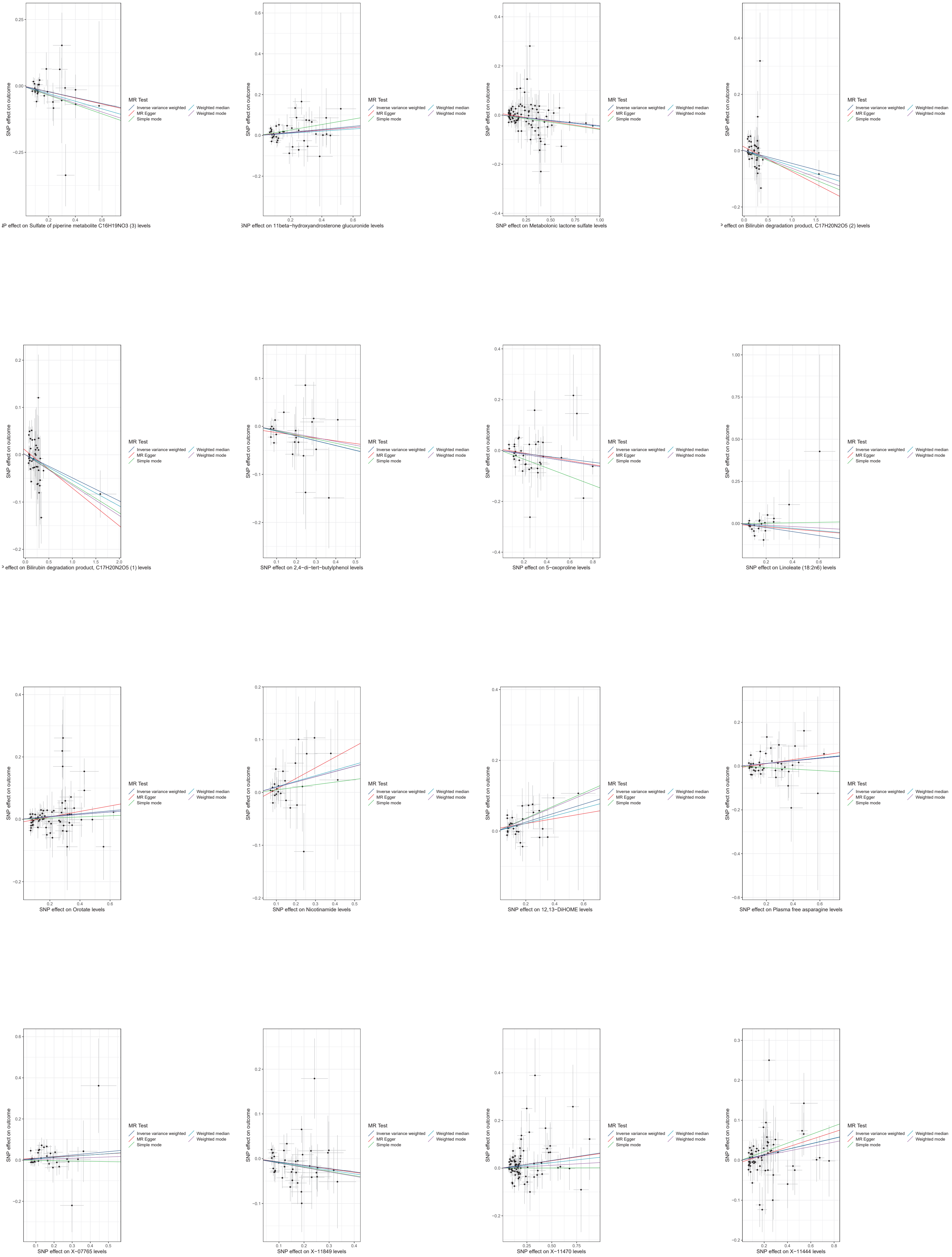
IP effect on Sulfate of piperine metabolite C16H19NO3 (3) levels
SNP effect on 11beta−hydroxyandrosterone glucuronide levels
SNP effect on Metabolonic lactone sulfate levels
effect on Bilirubin degradation product, C17H20N2O5 (2) levels
effect on Bilirubin degradation product, C17H20N2O5 (1) levels
SNP effect on 2,4−di−tert−butylphenol levels
SNP effect on 5−oxoproline levels
SNP effect on Linoleate (18:2n6) levels
SNP effect on Orotate levels
SNP effect on Nicotinamide levels
SNP effect on 12,13−DiHOME levels
SNP effect on Plasma free asparagine levels
SNP effect on X−07765 levels
SNP effect on X−11849 levels
SNP effect on X−11470 levels
SNP effect on X−11444 levels
SNP effect on outcome
MR Test
Inverse variance weighted
MR Egger
Simple mode
Weighted median
Weighted mode

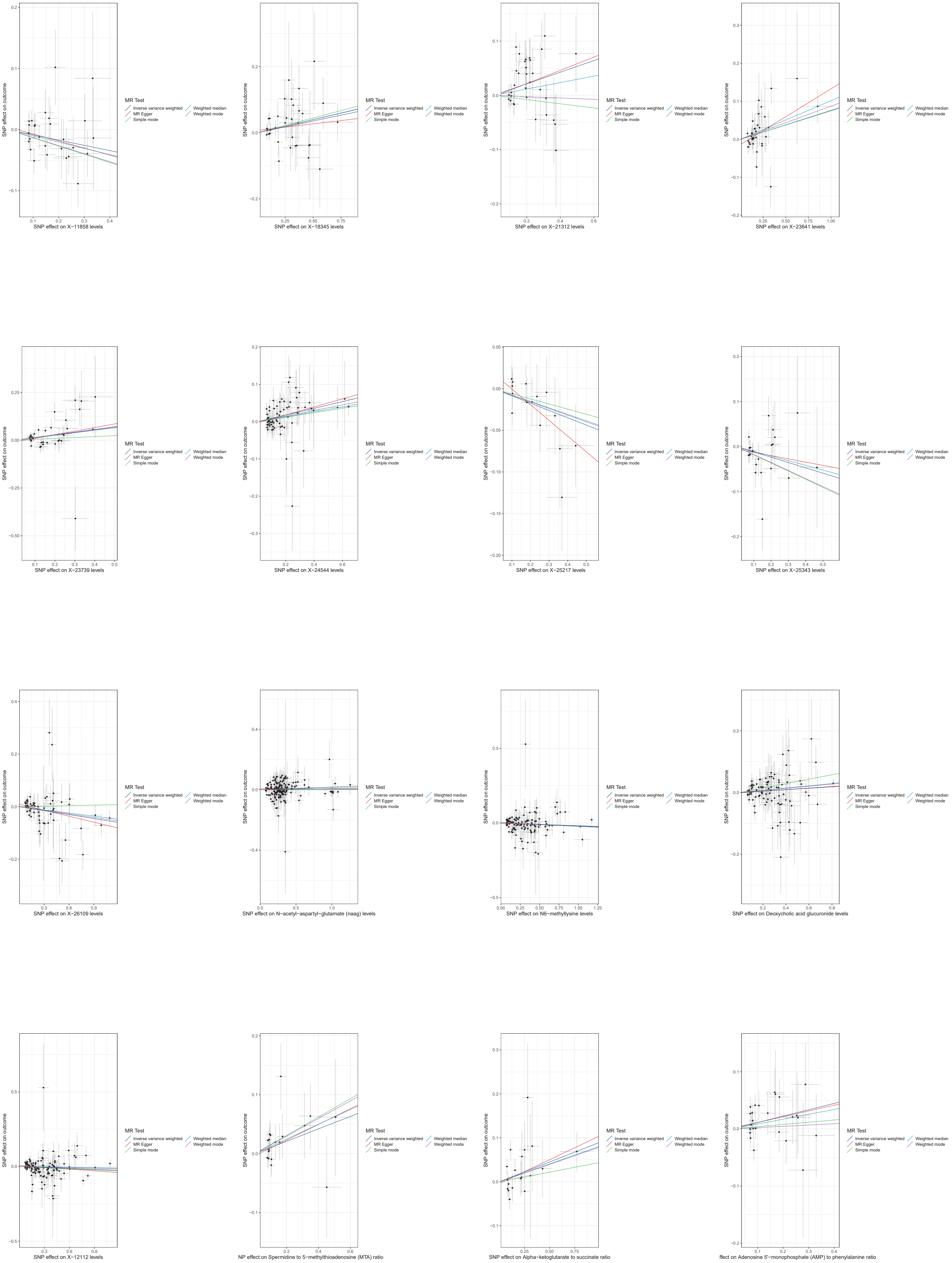
SNP effect on outcome
MR Test
Inverse variance weighted
MR Egger
Simple mode
Weighted median
Weighted mode
SNP effect on X−11858 levels
SNP effect on X−18345 levels
SNP effect on X−21312 levels
SNP effect on X−23641 levels
SNP effect on X−23739 levels
SNP effect on X−24544 levels
SNP effect on X−25217 levels
SNP effect on X−25343 levels
SNP effect on X−26109 levels
SNP effect on N−acetyl−aspartyl−glutamate (naag) levels
SNP effect on N6−methyllysine levels
SNP effect on Deoxycholic acid glucuronide levels
SNP effect on X−12112 levels
NP effect on Spermidine to 5−methylthioadenosine (MTA) ratio
SNP effect on Alpha−ketoglutarate to succinate ratio
ffect on Adenosine 5'−monophosphate (AMP) to phenylalanine ratio

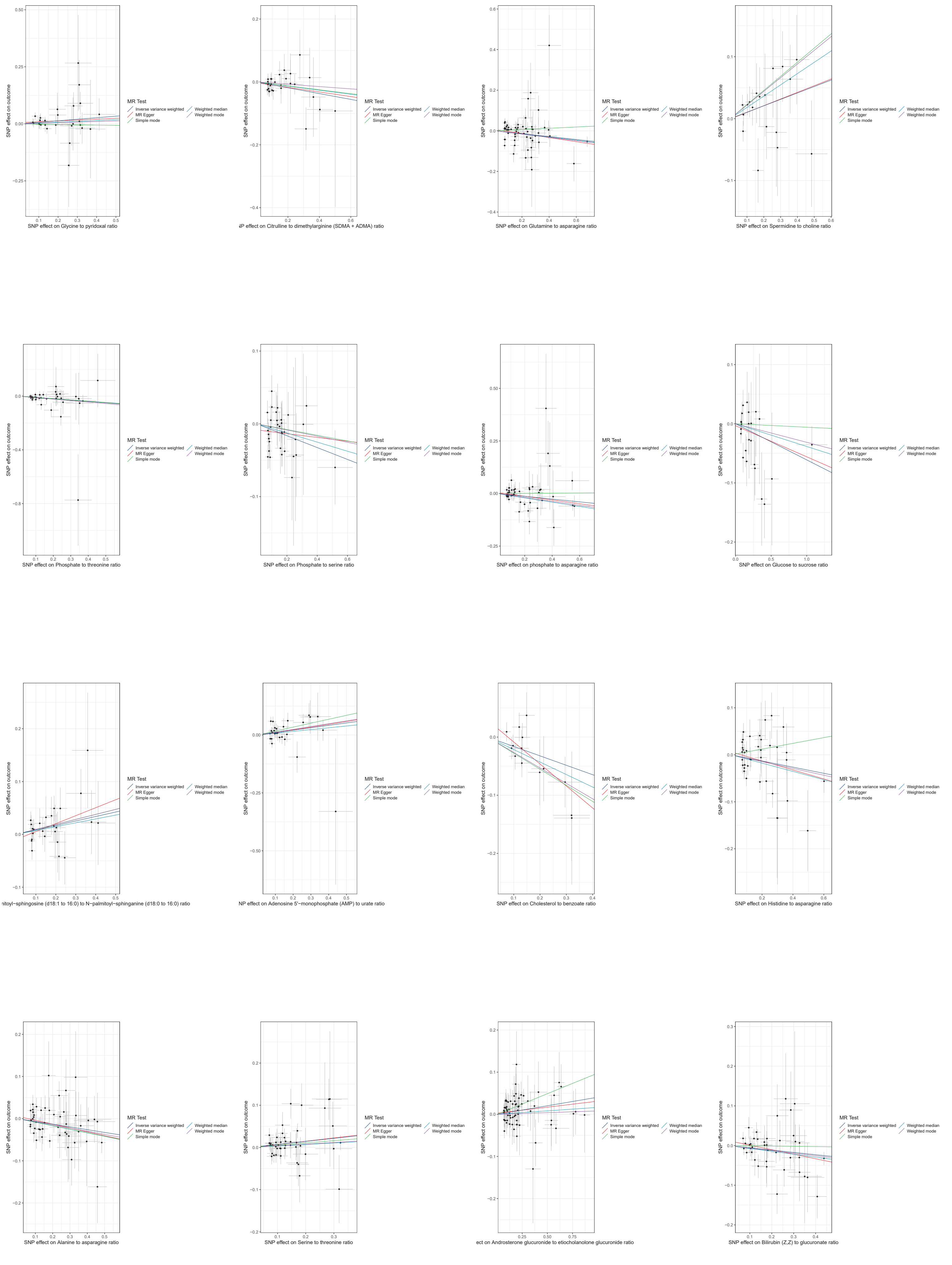
SNP effect on outcome
MR Test
Inverse variance weighted
MR Egger
Simple mode
Weighted median
Weighted mode
SNP effect on Glycine to pyridoxal ratio
NP effect on Citrulline to dimethylarginine (SDMA + ADMA) ratio
SNP effect on Glutamine to asparagine ratio
SNP effect on Spermidine to choline ratio
SNP effect on Phosphate to threonine ratio
SNP effect on Phosphate to serine ratio
SNP effect on phosphate to asparagine ratio
SNP effect on Glucose to sucrose ratio
nitoyl−sphingosine (d18:1 to 16:0) to N−palmitoyl−sphinganine (d18:0 to 16:0) ratio
NP effect on Adenosine 5'−monophosphate (AMP) to urate ratio
SNP effect on Cholesterol to benzoate ratio
SNP effect on Histidine to asparagine ratio
SNP effect on Alanine to asparagine ratio
SNP effect on Serine to threonine ratio
ect on Androsterone glucuronide to etiocholanolone glucuronide ratio
SNP effect on Bilirubin (Z,Z) to glucuronate ratio

Supplementary Figure 6. Scatter plots of two-sample MR analysis of plasma metabolites on osteoporosis.

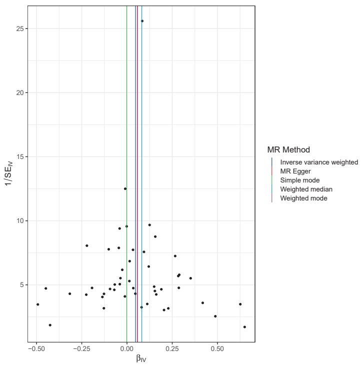
1/SE_IV
β_IV
MR Method
Inverse variance weighted
MR Egger
Simple mode
Weighted median
Weighted mode

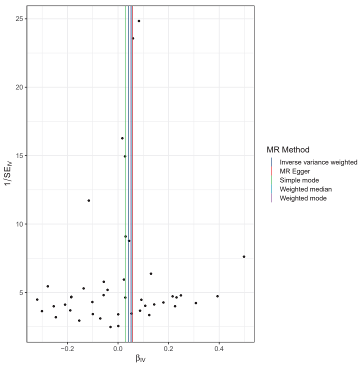
1/SE_IV
β_IV
MR Method
Inverse variance weighted
MR Egger
Simple mode
Weighted median
Weighted mode

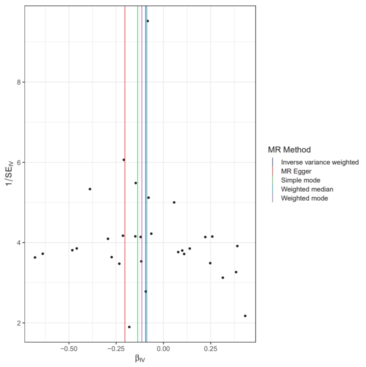
1/SE_IV
β_IV
MR Method
Inverse variance weighted
MR Egger
Simple mode
Weighted median
Weighted mode

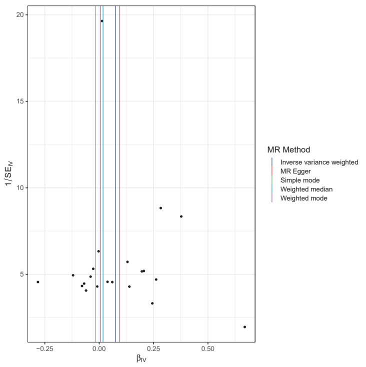
1/SE_IV
β_IV
MR Method
Inverse variance weighted
MR Egger
Simple mode
Weighted median
Weighted mode

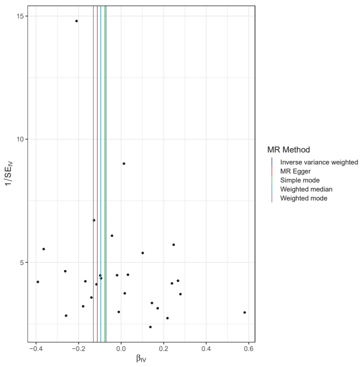
1/SE_IV
β_IV
MR Method
Inverse variance weighted
MR Egger
Simple mode
Weighted median
Weighted mode

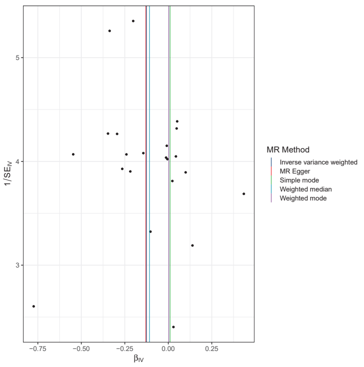
1/SE_IV
β_IV
MR Method
Inverse variance weighted
MR Egger
Simple mode
Weighted median
Weighted mode

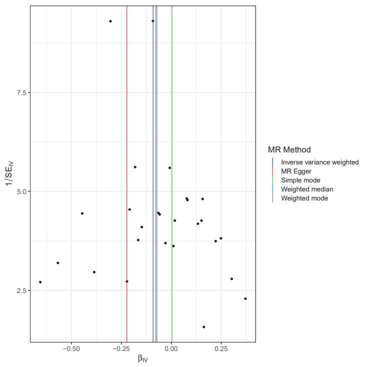
1/SE_IV
β_IV
MR Method
Inverse variance weighted
MR Egger
Simple mode
Weighted median
Weighted mode

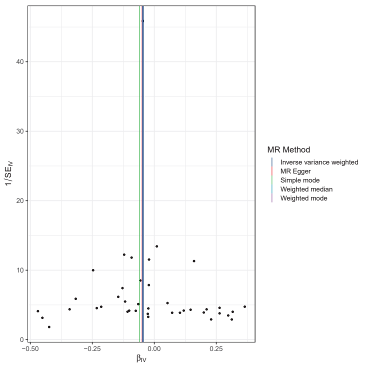
1/SE_IV
β_IV
MR Method
Inverse variance weighted
MR Egger
Simple mode
Weighted median
Weighted mode

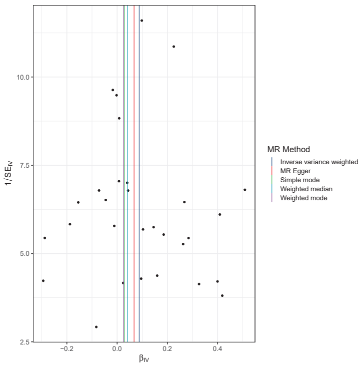
1/SE_IV
β_IV
MR Method
Inverse variance weighted
MR Egger
Simple mode
Weighted median
Weighted mode

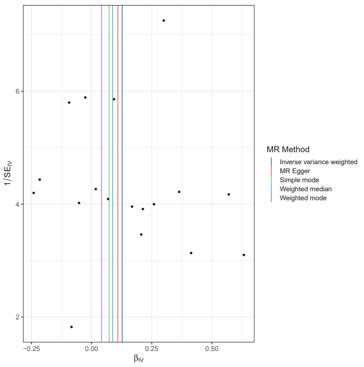
1/SE_IV
β_IV
MR Method
Inverse variance weighted
MR Egger
Simple mode
Weighted median
Weighted mode

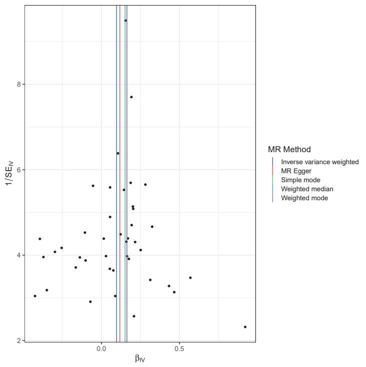
1/SE_IV
β_IV
MR Method
Inverse variance weighted
MR Egger
Simple mode
Weighted median
Weighted mode

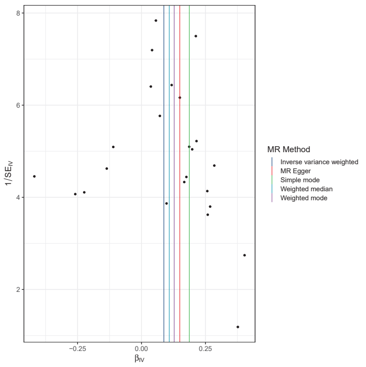
1/SE_IV
β_IV
MR Method
Inverse variance weighted
MR Egger
Simple mode
Weighted median
Weighted mode

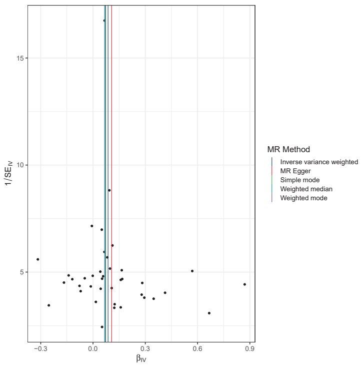
1/SE_IV
β_IV
MR Method
Inverse variance weighted
MR Egger
Simple mode
Weighted median
Weighted mode

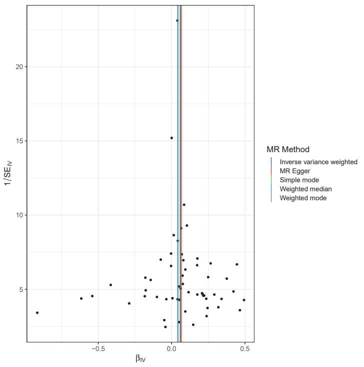
1/SE_IV
β_IV
MR Method
Inverse variance weighted
MR Egger
Simple mode
Weighted median
Weighted mode

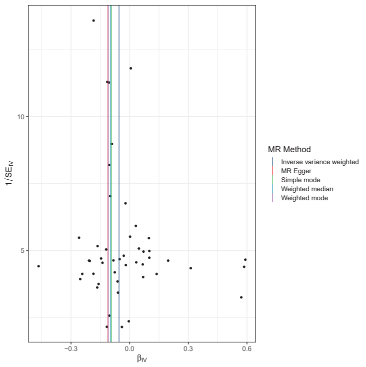
1/SE_IV
β_IV
MR Method
Inverse variance weighted
MR Egger
Simple mode
Weighted median
Weighted mode

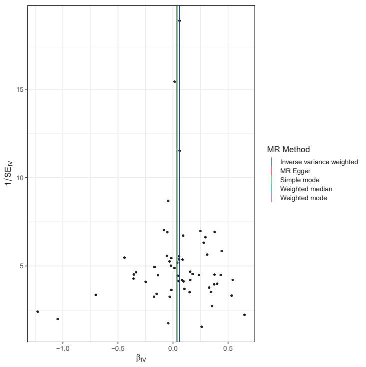
1/SE_IV
β_IV
MR Method
Inverse variance weighted
MR Egger
Simple mode
Weighted median
Weighted mode

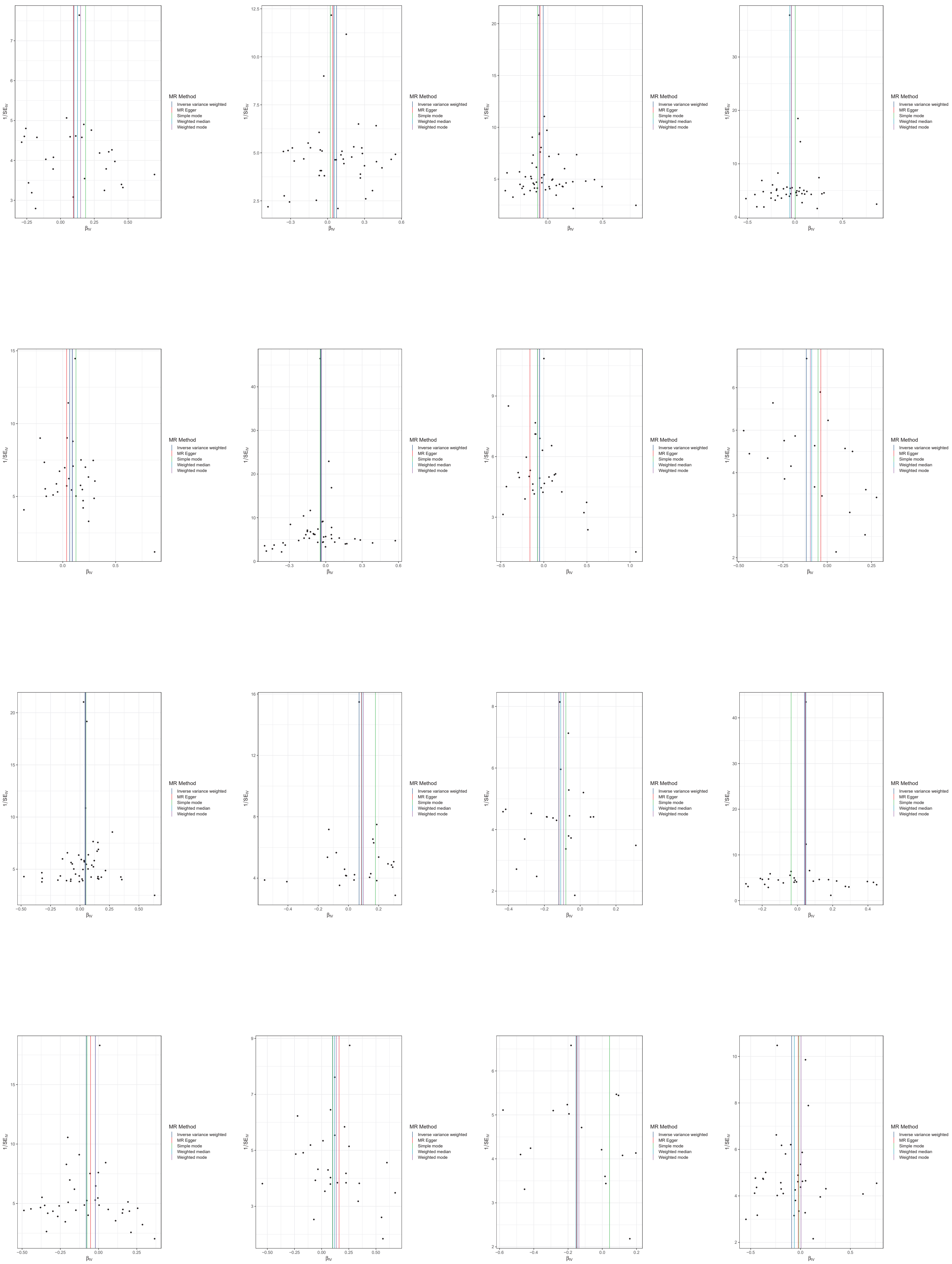
MR Method
Inverse variance weighted
MR Egger
Simple mode
Weighted median
Weighted mode
1/SE$_{IV}$
β_{IV}

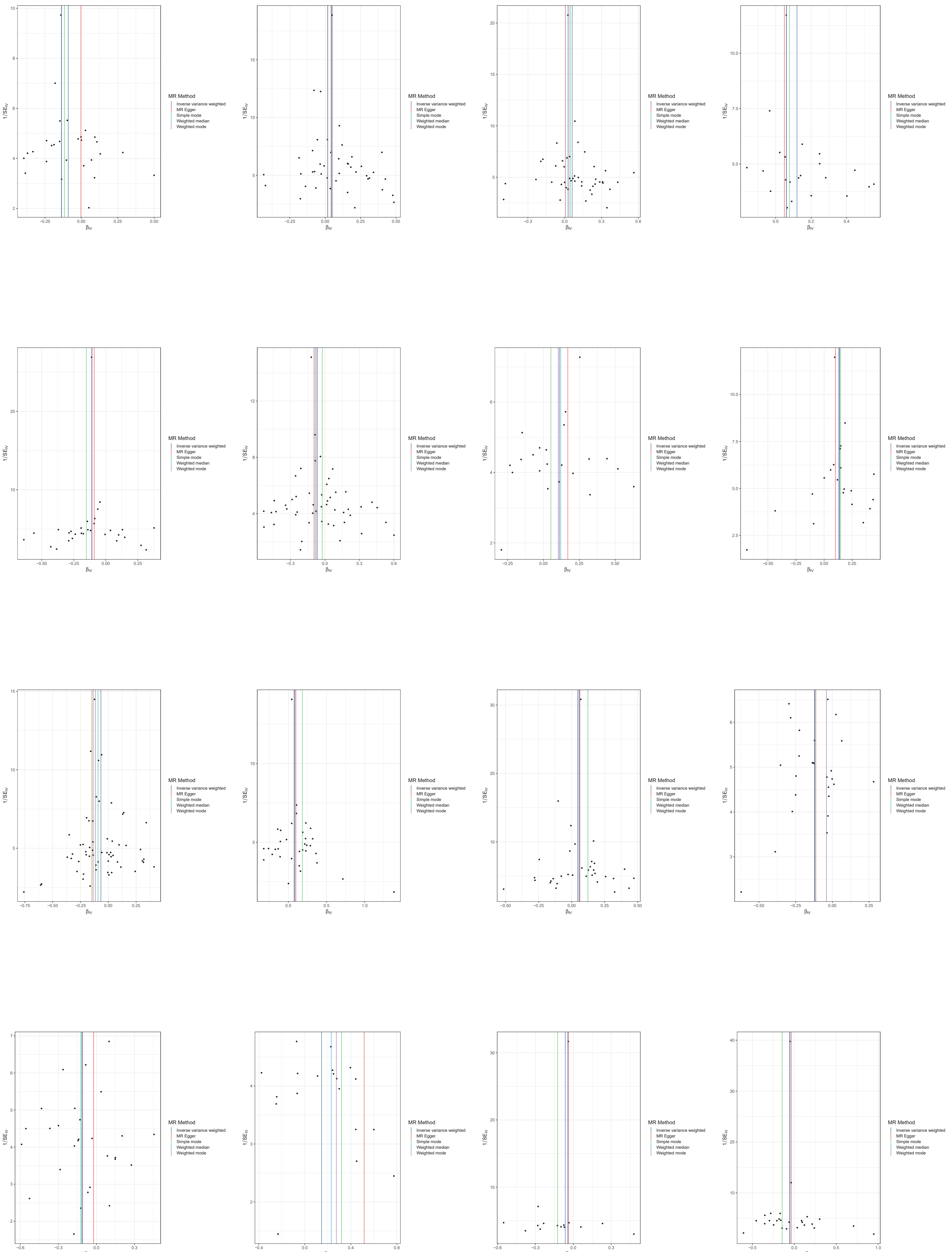
MR Method
Inverse variance weighted
MR Egger
Simple mode
Weighted median
Weighted mode
1/SE_IV
β_IV

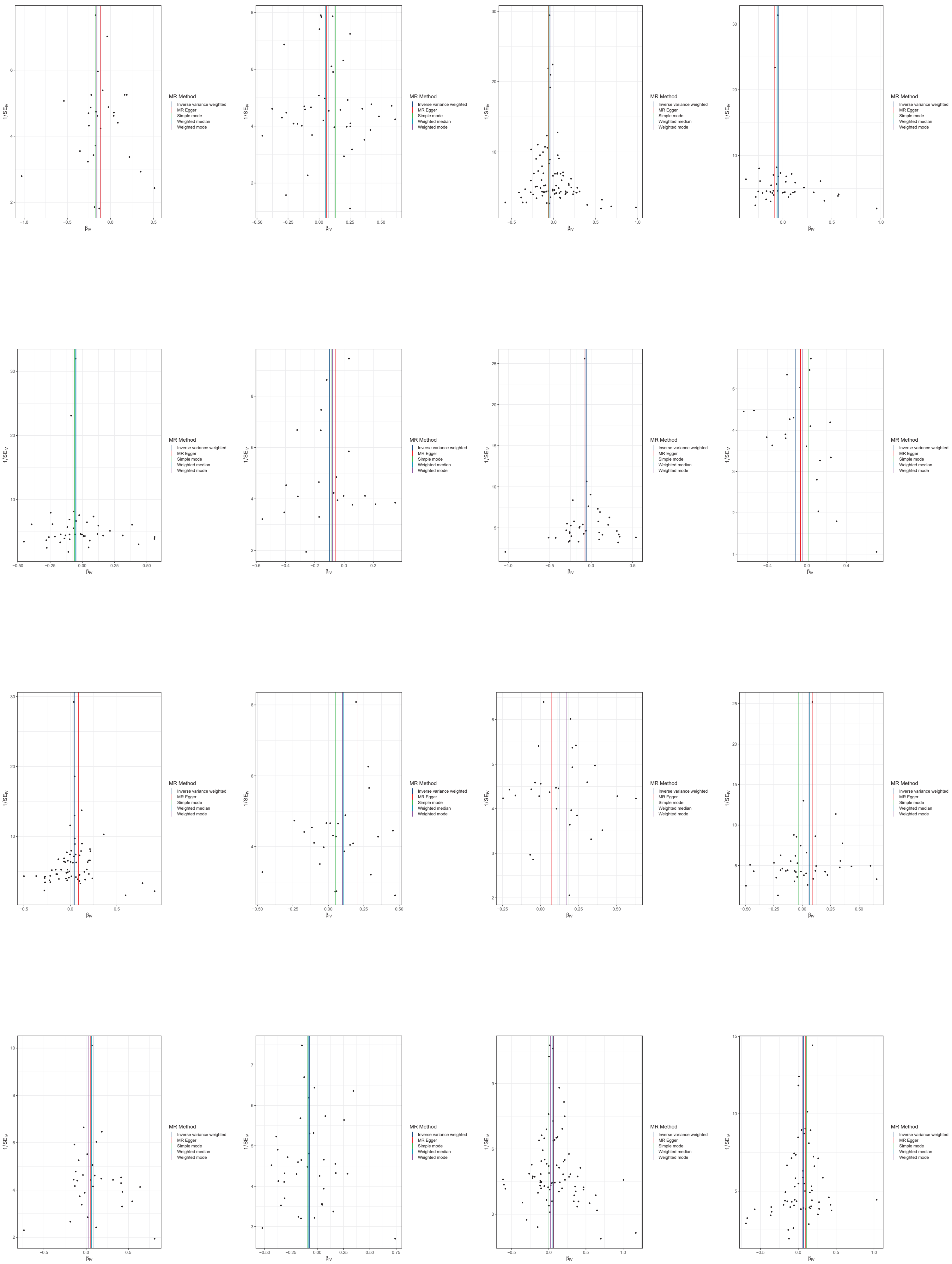
MR Method
Inverse variance weighted
MR Egger
Simple mode
Weighted median
Weighted mode
$1/SE_{IV}$
β_{IV}

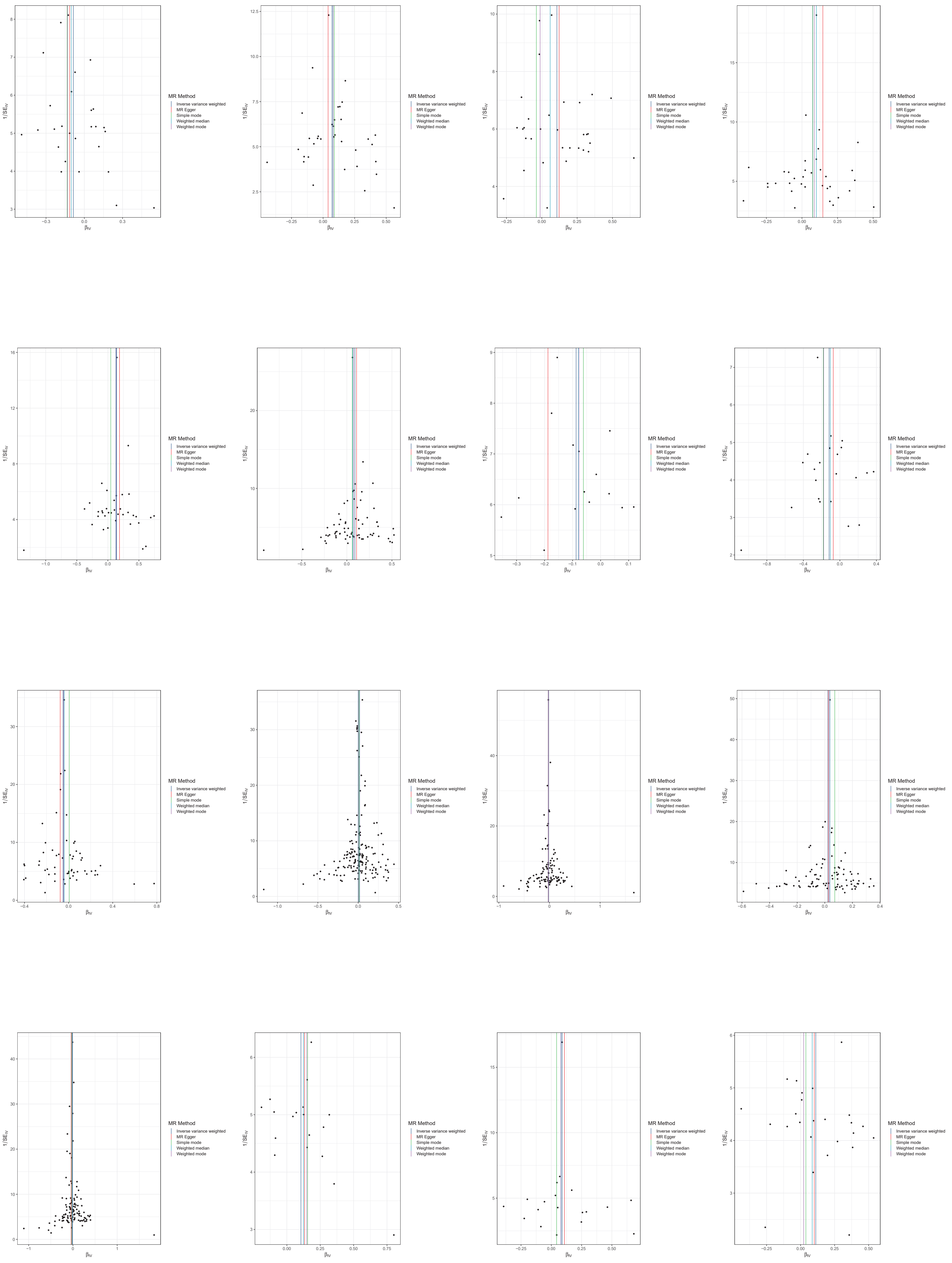
MR Method
Inverse variance weighted
MR Egger
Simple mode
Weighted median
Weighted mode
1/SE_IV
β_IV

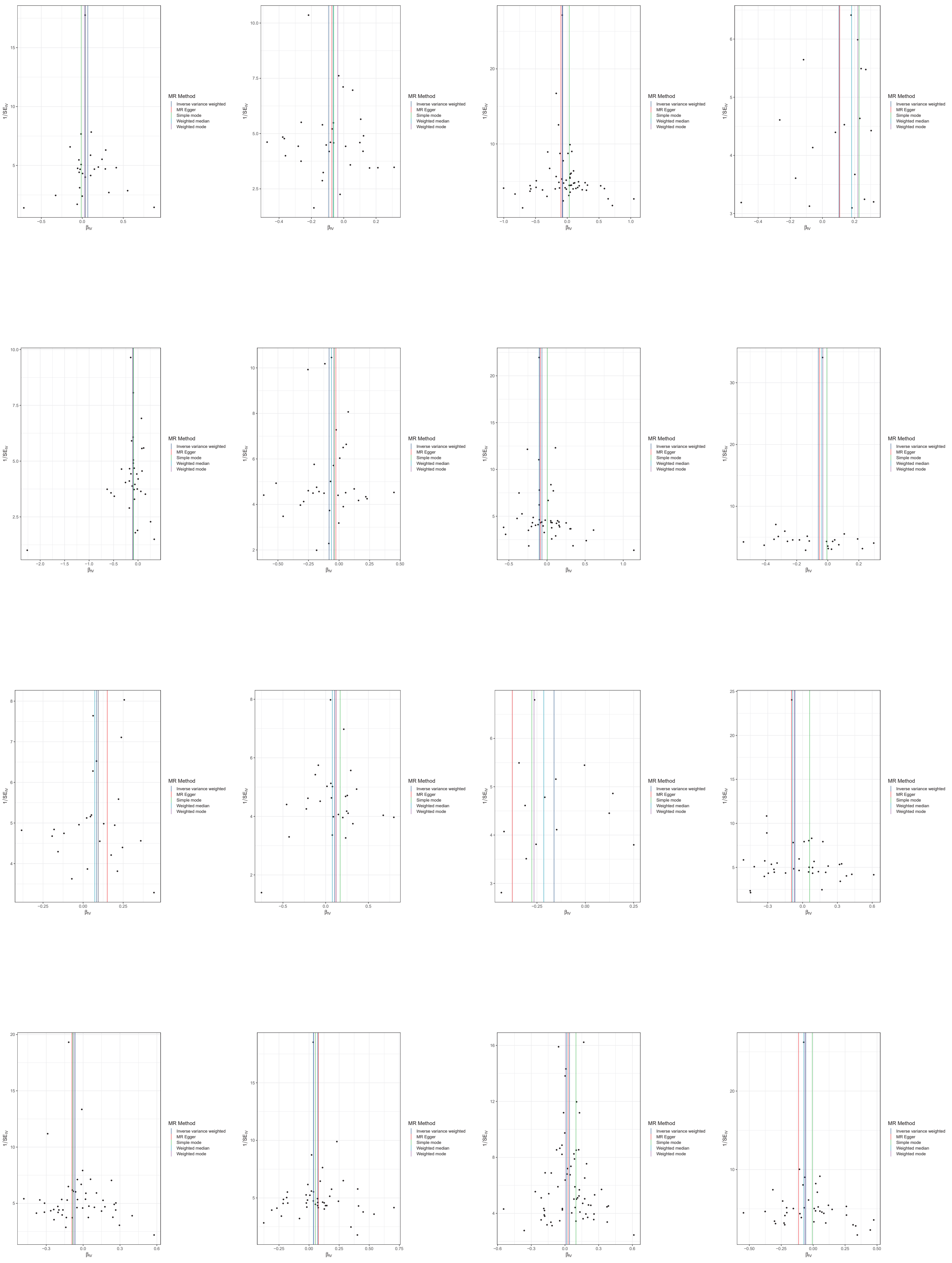
MR Method
Inverse variance weighted
MR Egger
Simple mode
Weighted median
Weighted mode
$1/SE_{IV}$
β_{IV}

Supplementary Figure 7. Funnel plots of two-sample MR analysis of plasma metabolites on osteoporosis.

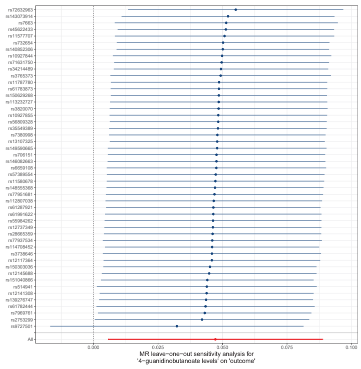
MR leave−one−out sensitivity analysis for
'4−guanidinobutanoate levels' on 'outcome'

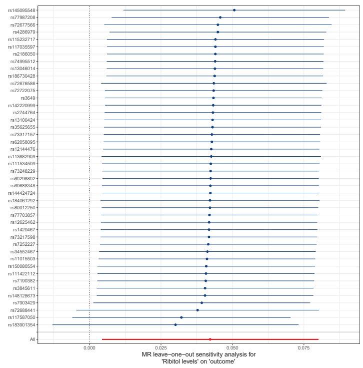
MR leave−one−out sensitivity analysis for
'Ribitol levels' on 'outcome'

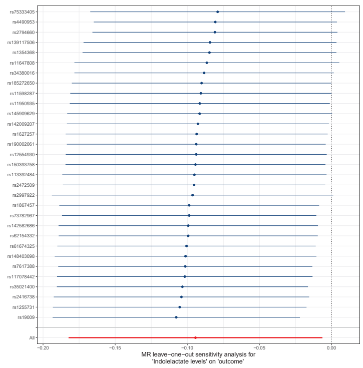
MR leave−one−out sensitivity analysis for
'Indolelactate levels' on 'outcome'

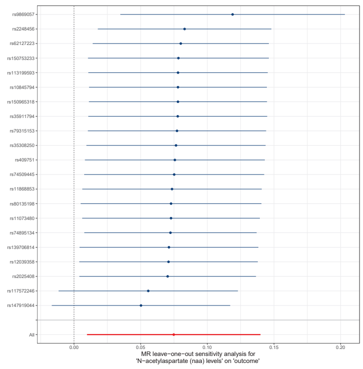
MR leave−one−out sensitivity analysis for
'N−acetylaspartate (naa) levels' on 'outcome'

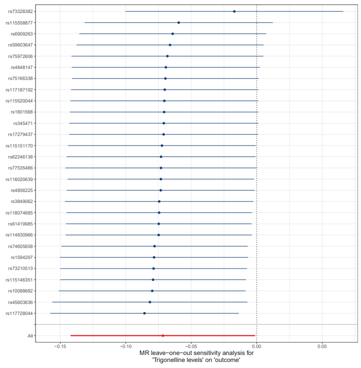
MR leave−one−out sensitivity analysis for
'Trigonelline levels' on 'outcome'

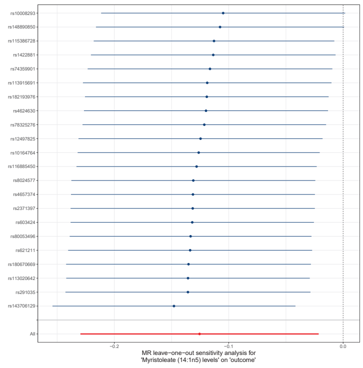
MR leave−one−out sensitivity analysis for
'Myristoleate (14:1n5) levels' on 'outcome'

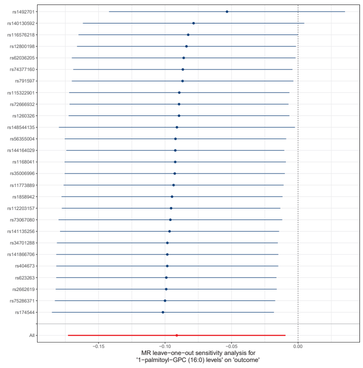
MR leave−one−out sensitivity analysis for
'1−palmitoyl−GPC (16:0) levels' on 'outcome'

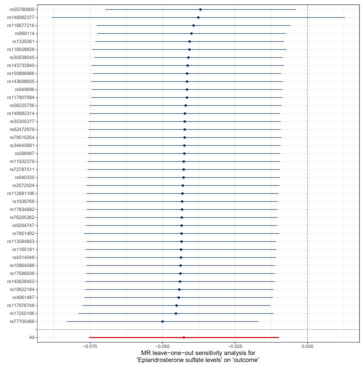
MR leave−one−out sensitivity analysis for
'Epiandrosterone sulfate levels' on 'outcome'

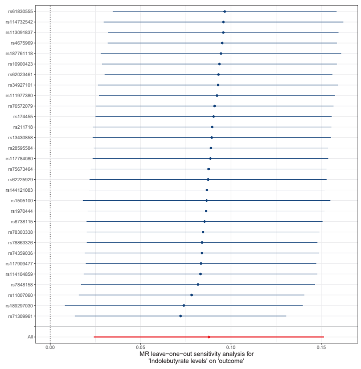
MR leave−one−out sensitivity analysis for
'Indolebutyrate levels' on 'outcome'

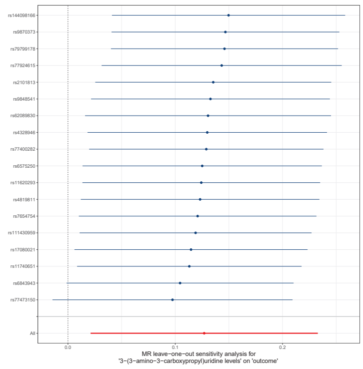
MR leave−one−out sensitivity analysis for
'3−(3−amino−3−carboxypropyl)uridine levels' on 'outcome'

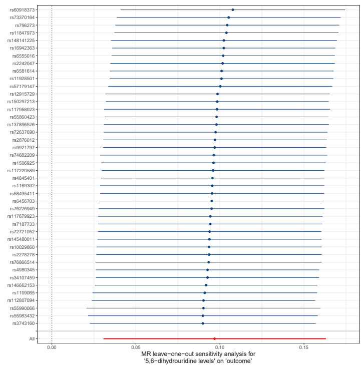
MR leave−one−out sensitivity analysis for
'5,6−dihydrouridine levels' on 'outcome'

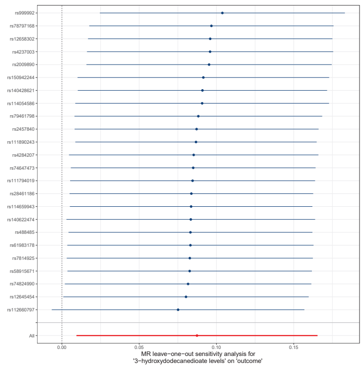
MR leave−one−out sensitivity analysis for
'3−hydroxydodecanedioate levels' on 'outcome'

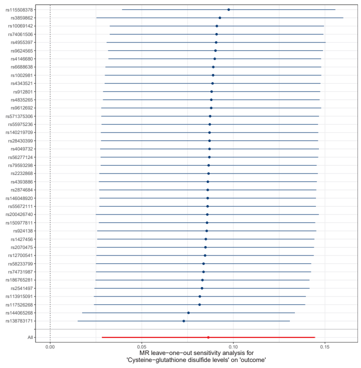
MR leave−one−out sensitivity analysis for
'Cysteine−glutathione disulfide levels' on 'outcome'

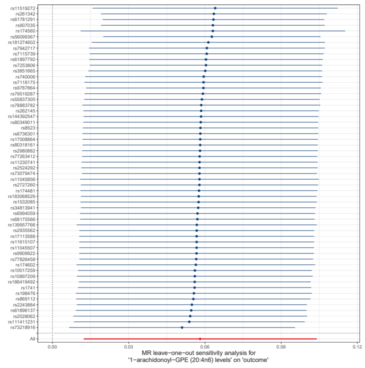
MR leave−one−out sensitivity analysis for
'1−arachidonoyl−GPE (20:4n6) levels' on 'outcome'

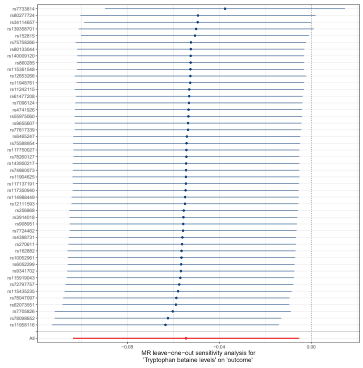
MR leave−one−out sensitivity analysis for
'Tryptophan betaine levels' on 'outcome'

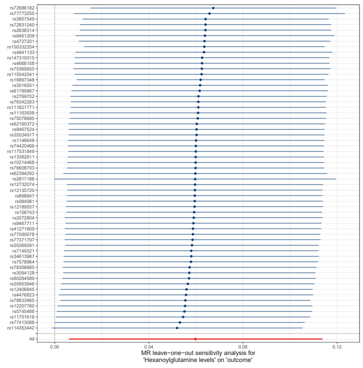
MR leave−one−out sensitivity analysis for
'Hexanoylglutamine levels' on 'outcome'

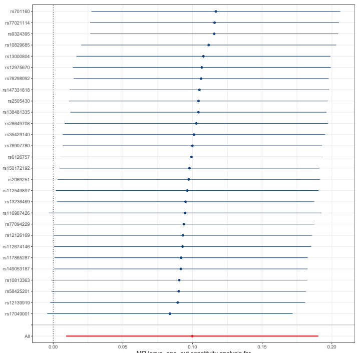
MR leave−one−out sensitivity analysis for
'Carotenoid levels (cryptoxanthin)' on 'outcome'

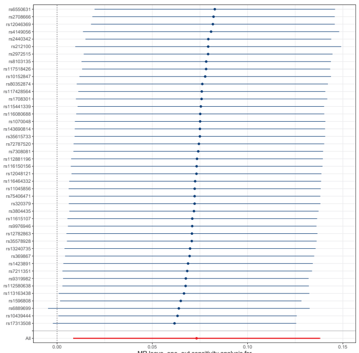
MR leave−one−out sensitivity analysis for
'5alpha−pregnan−3beta,20alpha−diol disulfate levels' on 'outcome'

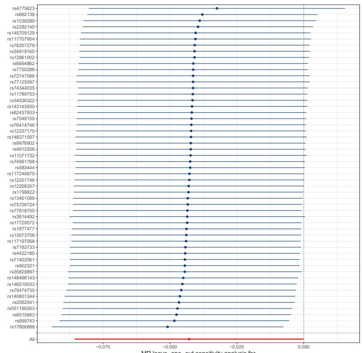
MR leave−one−out sensitivity analysis for
'Succinylcarnitine levels' on 'outcome'

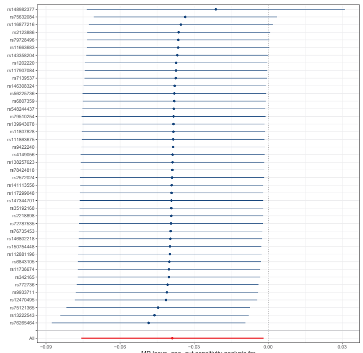
MR leave−one−out sensitivity analysis for
'5alpha−androstan−3beta,17beta−diol monosulfate (2) levels' on 'outcome'

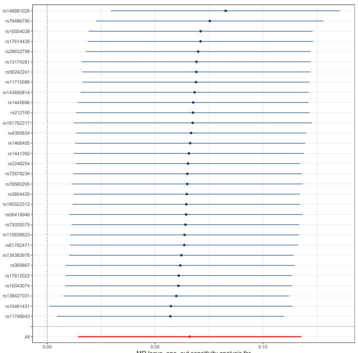
MR leave−one−out sensitivity analysis for
'5alpha−pregnan−diol disulfate levels' on 'outcome'

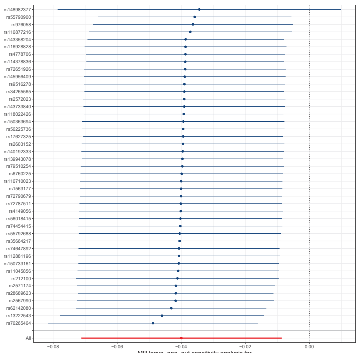
MR leave−one−out sensitivity analysis for
'5alpha−androstan−3alpha,17beta−diol monosulfate (1) levels' on 'outcome'

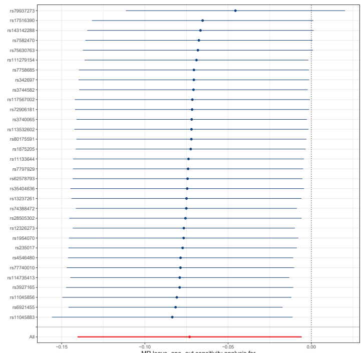
MR leave−one−out sensitivity analysis for
'5alpha−androstan−3alpha,17beta−diol disulfate levels' on 'outcome'

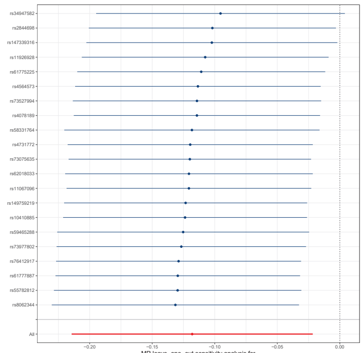
MR leave−one−out sensitivity analysis for
'2−hydroxyhippurate levels' on 'outcome'

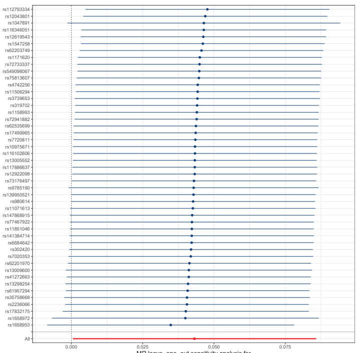
MR leave−one−out sensitivity analysis for
'3−methylglutaconate levels' on 'outcome'

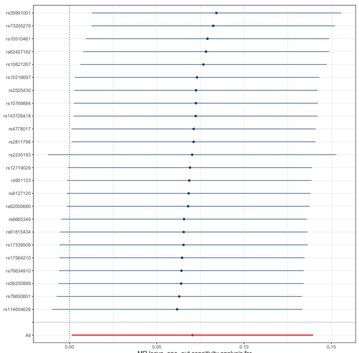
MR leave−one−out sensitivity analysis for
'S−methylcysteine levels' on 'outcome'

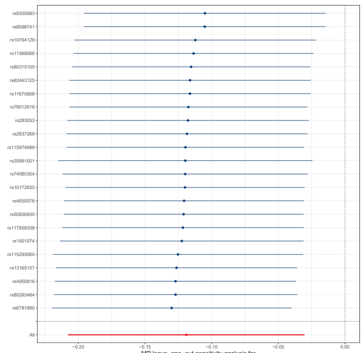
MR leave−one−out sensitivity analysis for
'S−methylcysteine sulfoxide levels' on 'outcome'

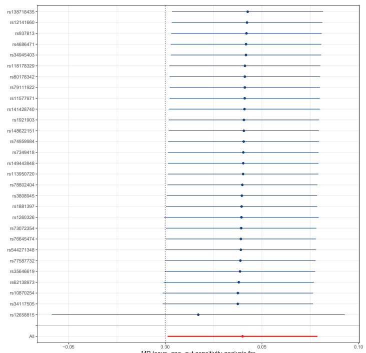
MR leave−one−out sensitivity analysis for
'Mannonate levels' on 'outcome'

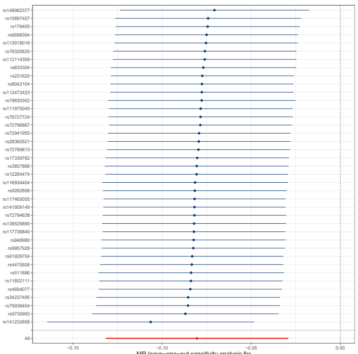
MR leave−one−out sensitivity analysis for
'Etiocholanolone glucuronide levels' on 'outcome'

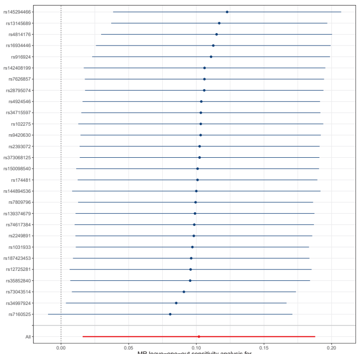
MR leave−one−out sensitivity analysis for
'Sphingomyelin (d18:1/22:1, d18:2/22:0, d16:1/24:1) levels' on 'outcome'

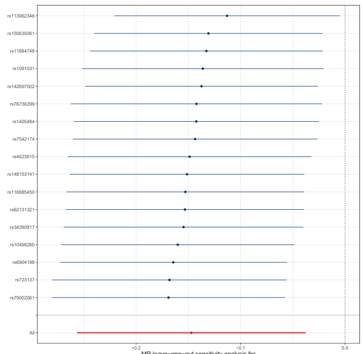
MR leave−one−out sensitivity analysis for
'Trans 3,4−methyleneheptanoate levels' on 'outcome'

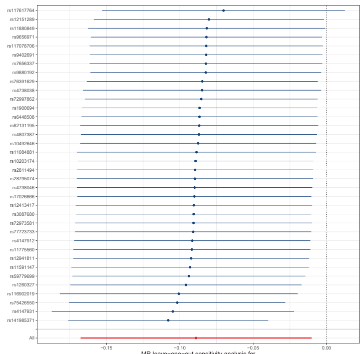
MR leave−one−out sensitivity analysis for
'Lactosyl−N−palmitoyl−sphingosine (d18:1/16:0) levels' on 'outcome'

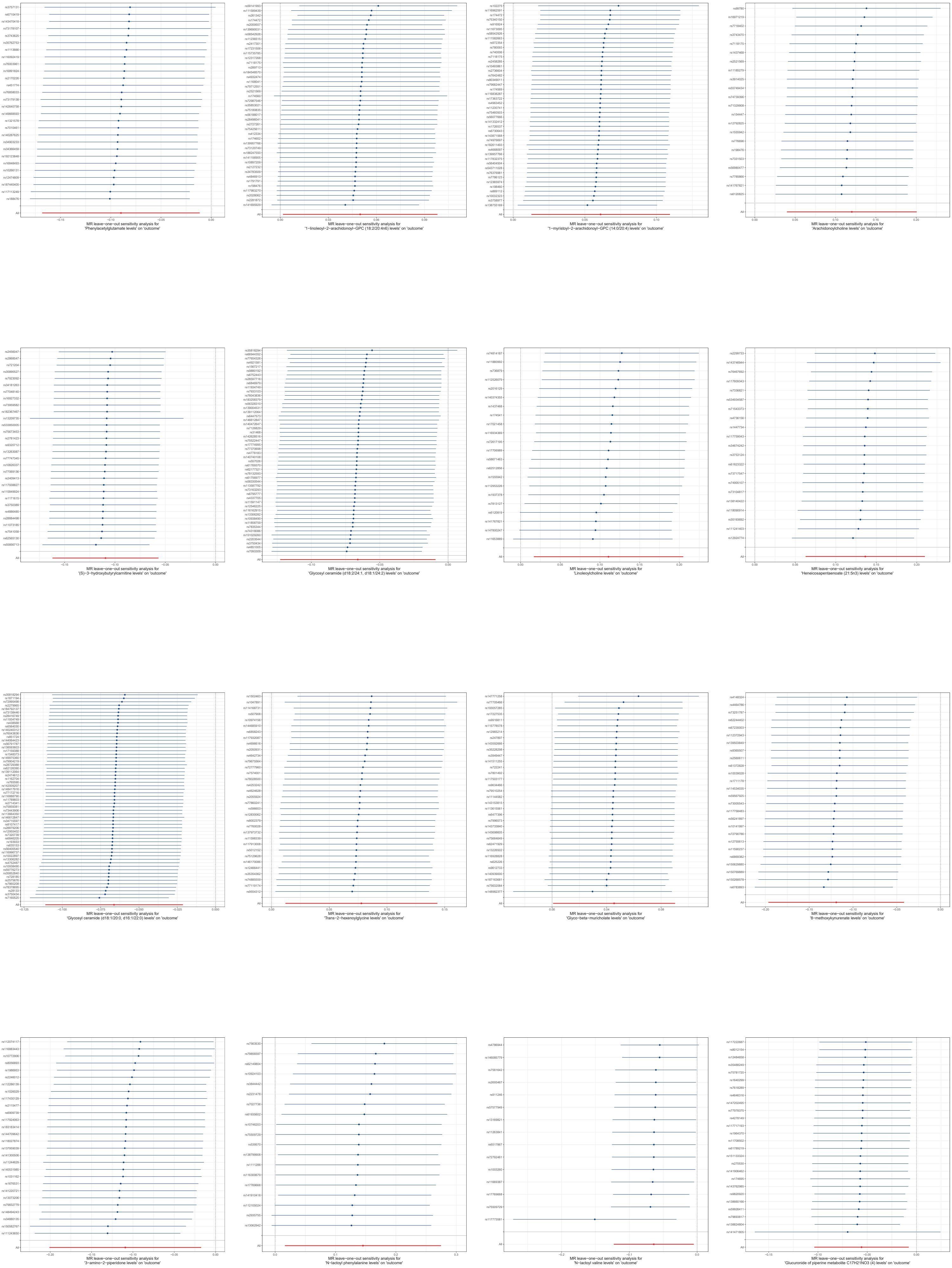
MR leave-one-out sensitivity analysis for
'Phenylacetylglutamate levels' on 'outcome'
MR leave-one-out sensitivity analysis for
'1-linoleoyl-2-arachidonoyl-GPC (18:2/20:4n6) levels' on 'outcome'
MR leave-one-out sensitivity analysis for
'1-myristoyl-2-arachidonoyl-GPC (14:0/20:4) levels' on 'outcome'
MR leave-one-out sensitivity analysis for
'Arachidonoylcholine levels' on 'outcome'
MR leave-one-out sensitivity analysis for
'(S)-3-hydroxybutyrylcarnitine levels' on 'outcome'
MR leave-one-out sensitivity analysis for
'Glycosyl ceramide (d18:2/24:1, d18:1/24:2) levels' on 'outcome'
MR leave-one-out sensitivity analysis for
'Linoleoylcholine levels' on 'outcome'
MR leave-one-out sensitivity analysis for
'Heneicosapentaenoate (21:5n3) levels' on 'outcome'
MR leave-one-out sensitivity analysis for
'Glycosyl ceramide (d18:1/20:0, d16:1/22:0) levels' on 'outcome'
MR leave-one-out sensitivity analysis for
'Trans-2-hexenoylglycine levels' on 'outcome'
MR leave-one-out sensitivity analysis for
'Glyco-beta-muricholate levels' on 'outcome'
MR leave-one-out sensitivity analysis for
'8-methoxykynurenate levels' on 'outcome'
MR leave-one-out sensitivity analysis for
'3-amino-2-piperidone levels' on 'outcome'
MR leave-one-out sensitivity analysis for
'N-lactoyl phenylalanine levels' on 'outcome'
MR leave-one-out sensitivity analysis for
'N-lactoyl valine levels' on 'outcome'
MR leave-one-out sensitivity analysis for
'Glucuronide of piperine metabolite C17H21NO3 (4) levels' on 'outcome'

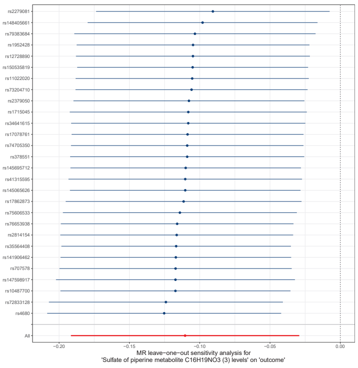
MR leave−one−out sensitivity analysis for
'Sulfate of piperine metabolite C16H19NO3 (3) levels' on 'outcome'

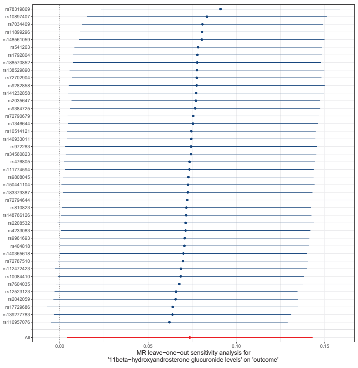
MR leave−one−out sensitivity analysis for
'11beta−hydroxyandrosterone glucuronide levels' on 'outcome'

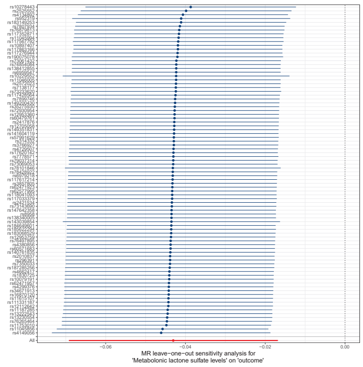
MR leave−one−out sensitivity analysis for
'Metabolonic lactone sulfate levels' on 'outcome'

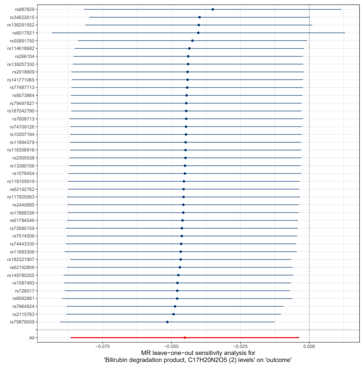
MR leave−one−out sensitivity analysis for
'Bilirubin degradation product, C17H20N2O5 (2) levels' on 'outcome'

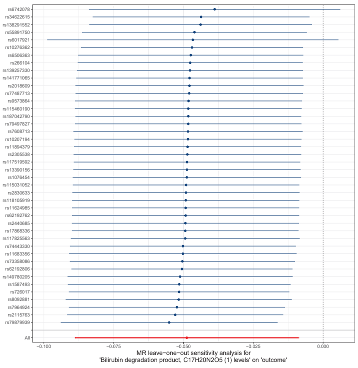
MR leave−one−out sensitivity analysis for
'Bilirubin degradation product, C17H20N2O5 (1) levels' on 'outcome'

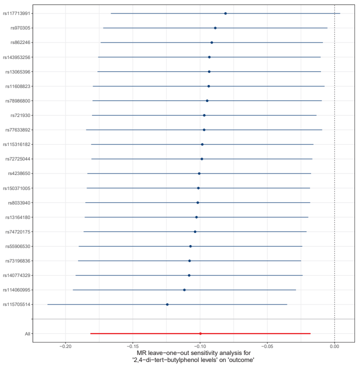
MR leave−one−out sensitivity analysis for
'2,4−di−tert−butylphenol levels' on 'outcome'

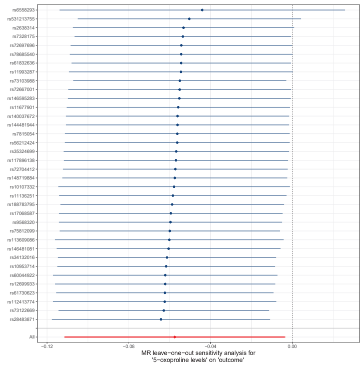
MR leave−one−out sensitivity analysis for
'5−oxoproline levels' on 'outcome'

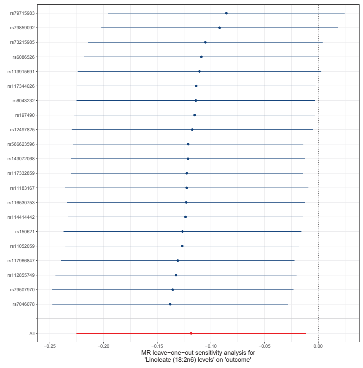
MR leave−one−out sensitivity analysis for
'Linoleate (18:2n6) levels' on 'outcome'

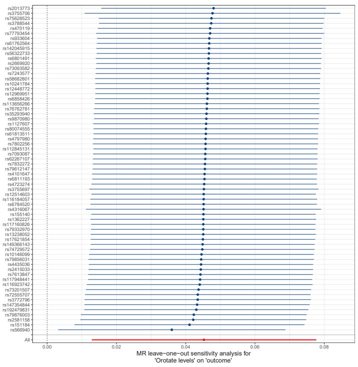
MR leave−one−out sensitivity analysis for
'Orotate levels' on 'outcome'

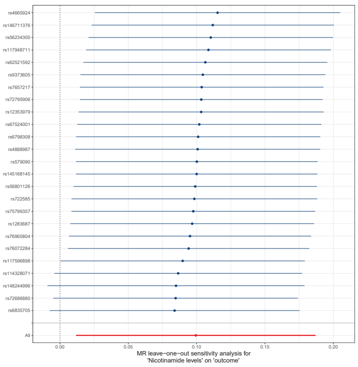
MR leave−one−out sensitivity analysis for
'Nicotinamide levels' on 'outcome'

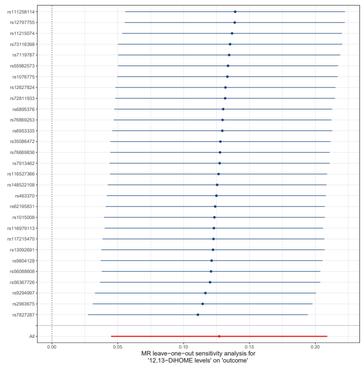
MR leave−one−out sensitivity analysis for
'12,13−DiHOME levels' on 'outcome'

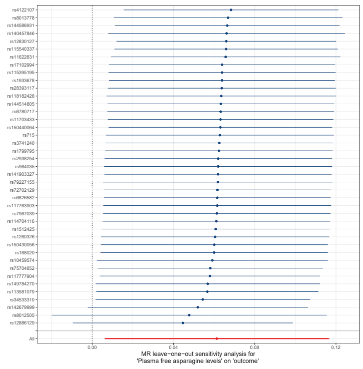
MR leave−one−out sensitivity analysis for
'Plasma free asparagine levels' on 'outcome'

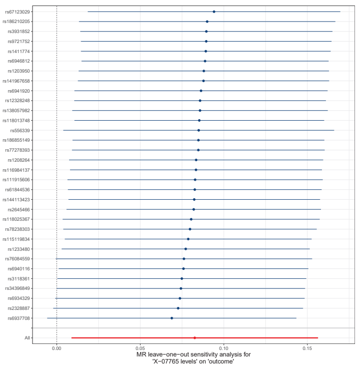
MR leave−one−out sensitivity analysis for
'X−07765 levels' on 'outcome'

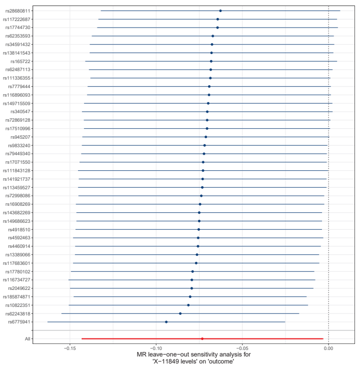
MR leave−one−out sensitivity analysis for
'X−11849 levels' on 'outcome'

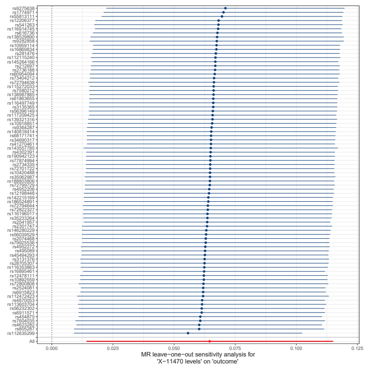
MR leave−one−out sensitivity analysis for
'X−11470 levels' on 'outcome'

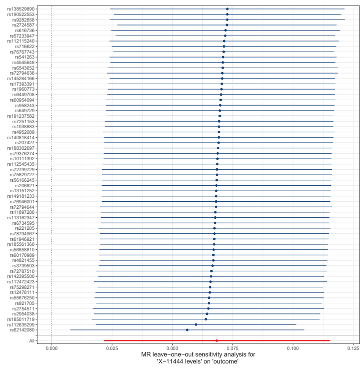
MR leave−one−out sensitivity analysis for
'X−11444 levels' on 'outcome'

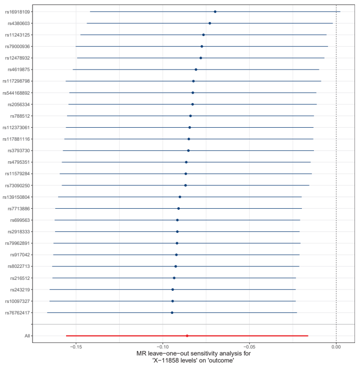
MR leave-one-out sensitivity analysis for
'X-11858 levels' on 'outcome'

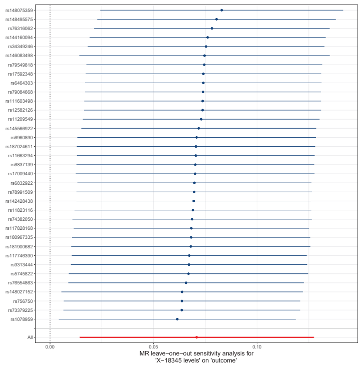
MR leave-one-out sensitivity analysis for
'X-18345 levels' on 'outcome'

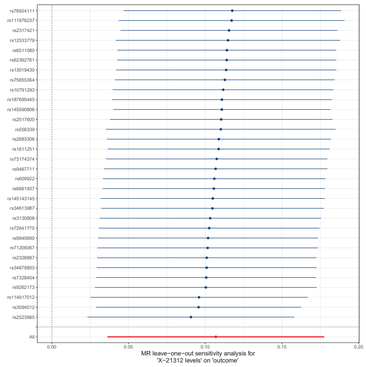
MR leave-one-out sensitivity analysis for
'X-21312 levels' on 'outcome'

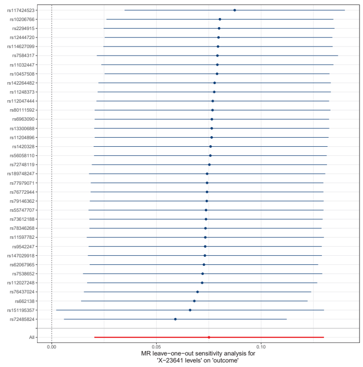
MR leave-one-out sensitivity analysis for
'X-23641 levels' on 'outcome'

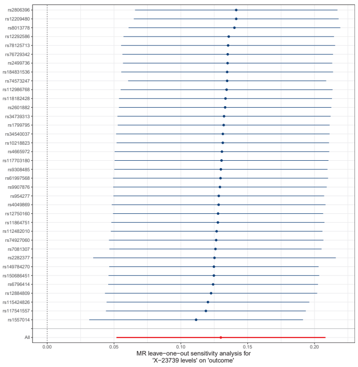
MR leave-one-out sensitivity analysis for
'X-23739 levels' on 'outcome'

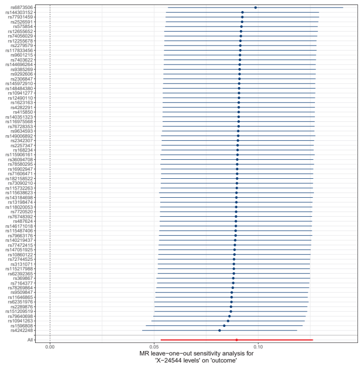
MR leave-one-out sensitivity analysis for
'X-24544 levels' on 'outcome'

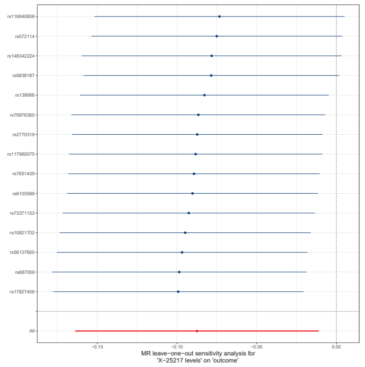
MR leave-one-out sensitivity analysis for
'X-25217 levels' on 'outcome'

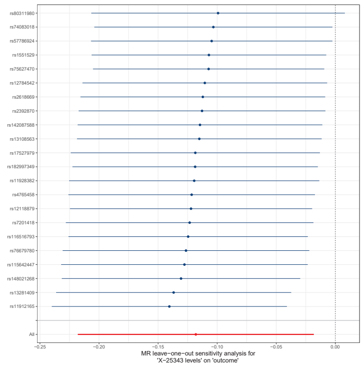
MR leave-one-out sensitivity analysis for
'X-25343 levels' on 'outcome'

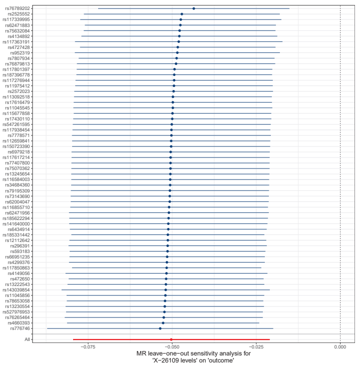
MR leave-one-out sensitivity analysis for
'X-26109 levels' on 'outcome'

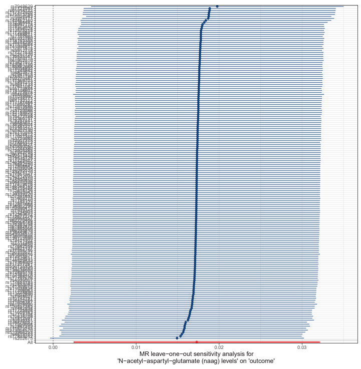
MR leave-one-out sensitivity analysis for
'N-acetyl-aspartyl-glutamate (naag) levels' on 'outcome'

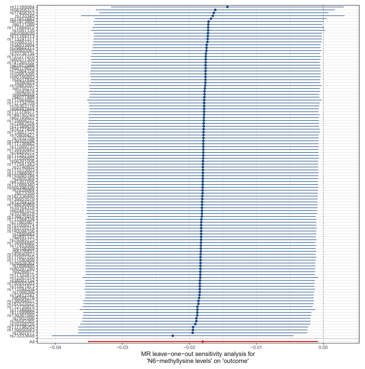
MR leave-one-out sensitivity analysis for
'N6-methyllysine levels' on 'outcome'

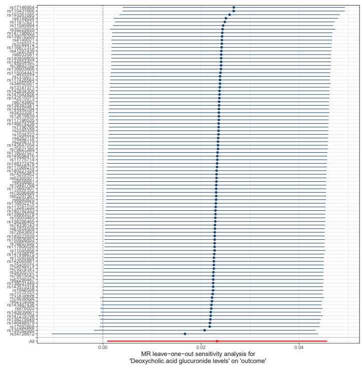
MR leave-one-out sensitivity analysis for
'Deoxycholic acid glucuronide levels' on 'outcome'

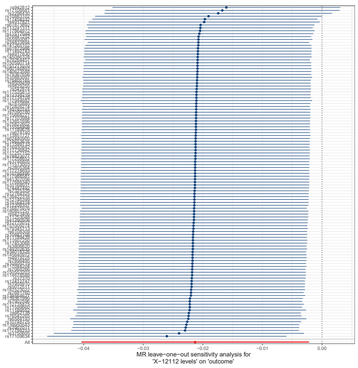
MR leave-one-out sensitivity analysis for
'X-12112 levels' on 'outcome'

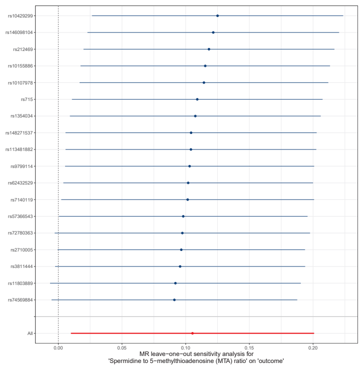
MR leave-one-out sensitivity analysis for
'Spermidine to 5-methylthioadenosine (MTA) ratio' on 'outcome'

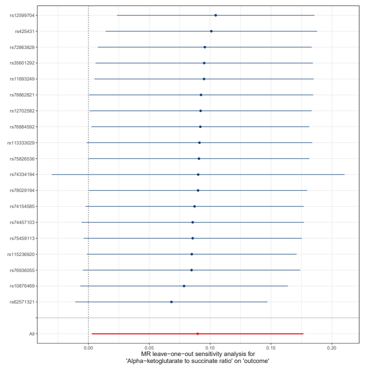
MR leave-one-out sensitivity analysis for
'Alpha-ketoglutarate to succinate ratio' on 'outcome'

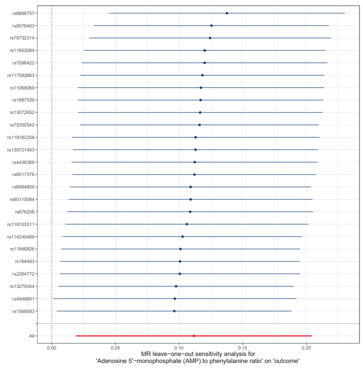
MR leave-one-out sensitivity analysis for
'Adenosine 5'-monophosphate (AMP) to phenylalanine ratio' on 'outcome'

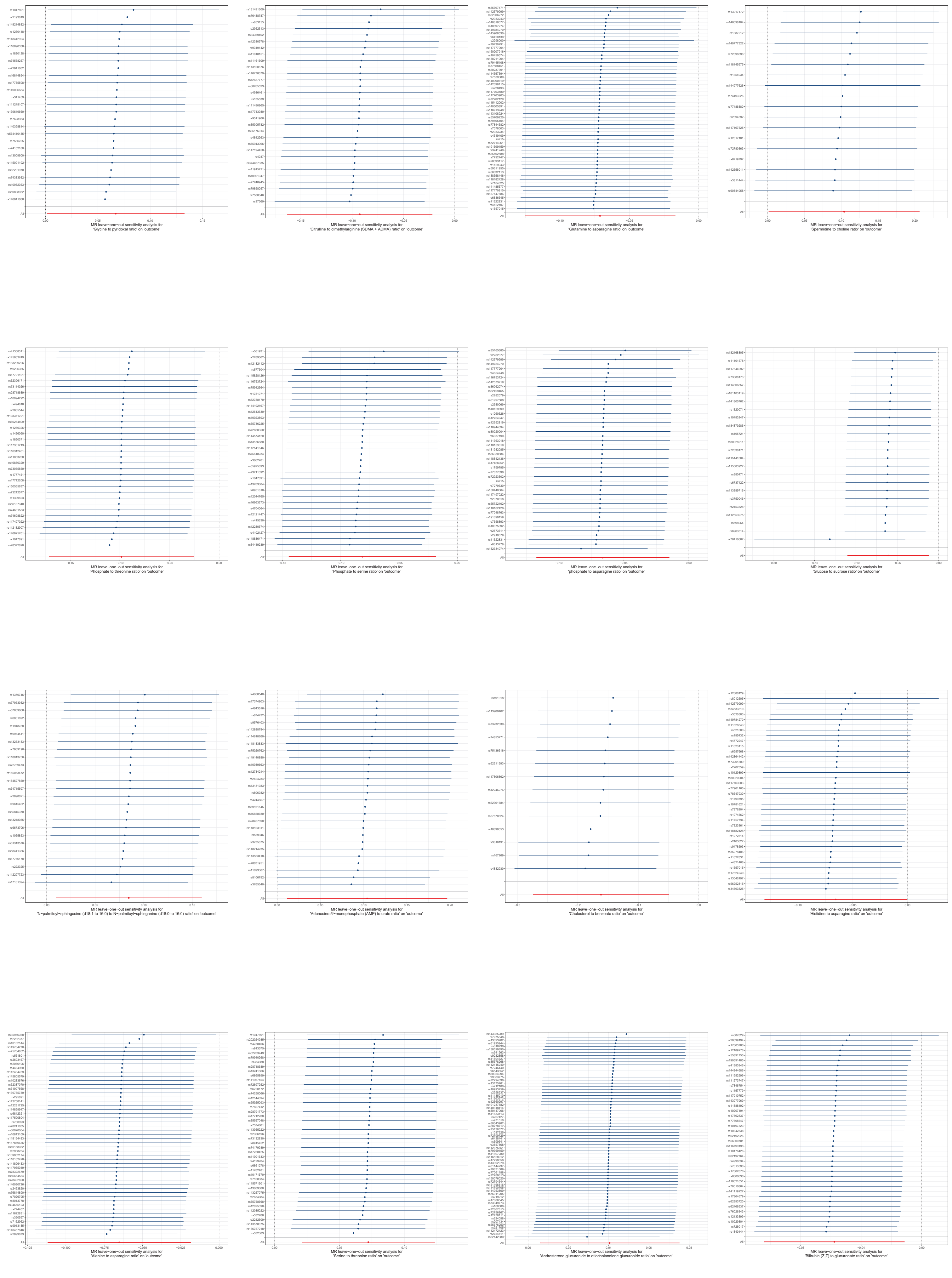
MR leave−one−out sensitivity analysis for 'Glycine to pyridoxal ratio' on 'outcome'
MR leave−one−out sensitivity analysis for 'Citrulline to dimethylarginine (SDMA + ADMA) ratio' on 'outcome'
MR leave−one−out sensitivity analysis for 'Glutamine to asparagine ratio' on 'outcome'
MR leave−one−out sensitivity analysis for 'Spermidine to choline ratio' on 'outcome'
MR leave−one−out sensitivity analysis for 'Phosphate to threonine ratio' on 'outcome'
MR leave−one−out sensitivity analysis for 'Phosphate to serine ratio' on 'outcome'
MR leave−one−out sensitivity analysis for 'phosphate to asparagine ratio' on 'outcome'
MR leave−one−out sensitivity analysis for 'Glucose to sucrose ratio' on 'outcome'
MR leave−one−out sensitivity analysis for 'N−palmitoyl−sphingosine (d18:1 to 16:0) to N−palmitoyl−sphinganine (d18:0 to 16:0) ratio' on 'outcome'
MR leave−one−out sensitivity analysis for 'Adenosine 5'−monophosphate (AMP) to urate ratio' on 'outcome'
MR leave−one−out sensitivity analysis for 'Cholesterol to benzoate ratio' on 'outcome'
MR leave−one−out sensitivity analysis for 'Histidine to asparagine ratio' on 'outcome'
MR leave−one−out sensitivity analysis for 'Alanine to asparagine ratio' on 'outcome'
MR leave−one−out sensitivity analysis for 'Serine to threonine ratio' on 'outcome'
MR leave−one−out sensitivity analysis for 'Androsterone glucuronide to etiocholanolone glucuronide ratio' on 'outcome'
MR leave−one−out sensitivity analysis for 'Bilirubin (Z,Z) to glucuronate ratio' on 'outcome'

Supplementary Figure 8. Leave-one-out plots of two-sample MR analysis of plasma metabolites on osteoporosis.
