# Supplementary material for: Gut microbiota, plasma metabolites, and osteoporosis: unraveling links via Mendelian randomization
Source: Front Microbiol. 2024 Jul 15;15:1433892. doi: 10.3389/fmicb.2024.1433892 (PMC11284117; doi:10.3389/fmicb.2024.1433892)
Supplement: Supplementary file 3 [file Data_Sheet_2.PDF]

Dataset ID and access address

Dataset	GWAS ID	Access address
Gut microbiota(GM)	ebi-a-GCST90016908~ebi-a-GCST90017118	http://www.mibiogen.org/
Plasma metabolites	GCST90199621~GCST90201020	https://www.ebi.ac.uk/gwas/downloads/summary-statistics
Osteoporosis(OP)	finngen_R10_M13_OSTEOPOROSIS	https://storage.googleapis.com/finngen-public-data-r10/summary_stats/finngen_R10_M13_OSTEOPOROSIS.gz

R code

**### Step 1: Install the R packages**
**### Step 2: Gut microbiota on Osteoporosis**
**### Step 3: Cyclic heat map**
**### Step 4: Plasma metabolites on Osteoporosis**
**### Step 5: GM on Plasma metabolites**
**### Step 6: Multivariate MR analysis**

Step 1: Install the R packages

```
if(!require("gwasrapidd")) install.packages("gwasrapidd",update = F,ask = F)
if(!require("stringr")) install.packages("stringr",update = F,ask = F)
if(!require("BiocManager")) install.packages("BiocManager",update = F,ask = F)
if(!require("data.table")) install.packages("data.table",update = F,ask = F)
if (!require("devtools")) install.packages("devtools",update = F,ask = F)
if (!require("MRPRESSO")) devtools::install_github("rondolab/MR-PRESSO")
if(!require("ggplot2")) install.packages("ggplot2",update = F,ask = F)
if(!require("ggsci")) install.packages("ggsci",update = F,ask = F)
if(!require("ggpubr")) install.packages("ggpubr",update = F,ask = F)
if(!require("circlize")) install.packages("circlize",update = F,ask = F)
if(!require("RColorBrewer")) install.packages("RColorBrewer",update = F,ask = F)
if (!require("TwoSampleMR")) devtools::install_github("MRCIEU/TwoSampleMR")
if (!require("TSMRhelper")) devtools::install_github("RightSZ/TSMRhelper")
if (!require("ComplexHeatmap")) BiocManager::install("ComplexHeatmap")
if(!require("dplyr")) install.packages("dplyr",update = F,ask = F)
if(!require("data.table")) install.packages("data.table",update = F,ask = F)
if(!require("forestploter")) install.packages("forestploter",update = F,ask = F)
devtools::install_github("explodecomputer/plinkbinr")
```

```
### Step 2: Gut microbiota on Osteoporosis
# Load R packages
rm(list = ls())
library(stringr)
library(data.table)
library(TwoSampleMR)
library(MRPRESSO)
library(dplyr)
library(ggsci)
library(ggplot2)
library(TSMRhelper)
library(forestploter)
library(grid)
library(stringr)
library(grDevices)

# Read the local outcome (Osteoporosis)
id<-""
outcome_all<-fread("./finngen_R10_M13_OSTEOPOROSIS.gz",data.table = F)

# Get full Gut microbiota IEU exposure id data
if(file.exists("ao.csv")){ao<-read.csv(file = "ao.csv")}else{
  while(TRUE){
    message_to_next <<- TRUE
    error_to_next <<- FALSE
    try({withCallingHandlers(ao <- available_outcomes(),
                             message = function(c) if
(stringr::str_detect(as.character(c),"Failed to")) message_to_next <<- FALSE)
      error_to_next <<- TRUE})
    if(message_to_next == TRUE&error_to_next == TRUE) { break }
  }
  write.csv(ao,file = "ao.csv")
}
ao<-read.csv("ao.csv")
id_exp<-subset(ao,pmid==33462485)
id_exp<-id_exp[!str_detect(id_exp$trait,"unknown"),]
exp<-list()
i <- 1
for (ids in id_exp[1:nrow(id_exp),]$id) {
  print(ids)
  while(TRUE){
    message_to_next <<- TRUE
    error_to_next <<- FALSE
    try({withCallingHandlers(exp_clumped<-extract_instruments(outcomes = ids,
```

```
                                                   p1 = 1e-05,
                                                   clump = TRUE,
                                                   r2 = 0.001,
                                                   kb = 10000,
                                                   access_token =
NULL),
                   message = function(c) if
(stringr::str_detect(as.character(c),"Failed to")) message_to_next <<- FALSE)
    error_to_next <<- TRUE})
   if(message_to_next == TRUE&error_to_next == TRUE) { break }
  }
  if(is.null(exp_clumped))next
  exp_clumped$exposure<-id_exp[id_exp$id==exp_clumped$id.exposure[1],]$trait
  exp[[i]]<-exp_clumped
  i<-i+1
}
exp_clumped<-do.call(rbind,exp)

write.csv(exp_clumped,file = "exp_clumped.csv",row.names = F)

# Calculating the F-statistic
exp_clumped<-read.csv("exp_clumped.csv")
F_cal<-function(beta,se){F.Statistics<-beta^2/se^2}
F.Statistics<-F_cal(beta =exp_clumped$beta.exposure,se =exp_clumped$se.exposure )
exp_clumped$F.Statistics <- F.Statistics

# Get outcome
if(id==""){
  snp<-unique(exp_clumped$SNP)
  outcome<-outcome_all[outcome_all$rsids%in%snp,]
  outcome_dat<-format_data(outcome,
                        type = "outcome",
                        beta_col = "beta",
                        se_col = "sebeta",
                        effect_allele_col = "alt",
                        other_allele_col = "ref",
                        snp_col = "rsids",
                        chr_col = "#chrom",
                        pos_col = "pos",
                        pval_col = "pval",
                        eaf_col = "maf")
}else{
  #
  while(TRUE){
```

```
    message_to_next <<- TRUE
    error_to_next <<- FALSE
    try({withCallingHandlers(outcome_dat <- extract_outcome_data(snps =
exp_clumped$SNP,
                                                                 outcomes = id,
                                                                 proxies = F,
                                                                 access_token =
NULL
    ),
    message = function(c) if (stringr::str_detect(as.character(c),"Failed to"))
message_to_next <<- FALSE)
      error_to_next <<- TRUE})
    if(message_to_next == TRUE&error_to_next == TRUE) { break }
  }
}
write.csv(outcome_dat,file = "outcome.csv",row.names = F)
outcome_dat<-read.csv("outcome.csv")
```

# Harmonise data

```
dat<-harmonise_data(exposure_dat = exp_clumped,outcome_dat = outcome_dat)
dat<-dat[dat$mr_keep,]
write.csv(dat,file = "dat.csv",row.names = F)
```

# MR analysis

```
dat<-split(dat,dat$id.exposure)
library(parallel)
cl <- makeCluster(detectCores())
res<-parLapply(cl,dat,mr)
stopCluster(cl)
res<-do.call(rbind,res)
res<-generate_odds_ratios(res)
p<-p.adjust(res$pval,   #
            method ="BH")
res$padjust<-p
write.csv(res,file = "res.csv",row.names = F)
res<-read.csv("res.csv")
dat<-do.call(rbind,dat)
```

# Heterogeneity analysis

```
heterogeneity<-mr_heterogeneity(dat)
write.csv(heterogeneity,file = "heterogeneity.csv",row.names = F)
```

# Pleiotropy test

```
pleiotropy_test<-mr_pleiotropy_test(dat)
```

```
write.csv(pleiotropy_test,file = "pleiotropy_test.csv",row.names = F)
```

# MR-PRESSO analysis

```
mp<-list()
mps<-unique(dat$id.exposure)
i=1
for (mpi in mps) {
  data<-dat[dat$id.exposure==mpi,]
  if(nrow(data)<4){next}
  # MRPRESSO
  mp[[i]]<-mr_presso(BetaOutcome = "beta.outcome",
                     BetaExposure = "beta.exposure",
                     SdOutcome = "se.outcome",
                     SdExposure = "se.exposure",
                     OUTLIERtest = TRUE,
                     DISTORTIONtest = TRUE,
                     data = data,
                     NbDistribution = 1000,
                     SignifThreshold = 0.05)
  mp[[i]]$phe <- data$exposure[1]
  i=i+1
}
result_mp_all<- data.frame()
for (i in 1:length(mp)) {
  print(mp[[i]]$phe)
  result_mp <- mp[[i]]$`Main MR results`
  result_mp$RSSobs <- mp[[i]]$`MR-PRESSO results`$`Global Test`$RSSobs
  result_mp$Pvalue <- mp[[i]]$`MR-PRESSO results`$`Global Test`$Pvalue
  result_mp$phe <- mp[[i]]$phe
  result_mp_all <- rbind(result_mp_all,result_mp)
}
table(is.na(result_mp_all$`Causal Estimate`))
write.csv(result_mp_all,file = "MRPRESSO.csv",row.names = F)
```

# Reverse MR analysis

```
if(id==""){
  exp<-outcome_all
  exp$pval<-as.numeric(exp$pval)
  exp<-subset(exp,pval<1e-05)
  exp<-format_data(exp,
                   snp_col = "rsids",
                   chr_col = "#chrom",
                   pos_col = "pos",
                   effect_allele_col = "alt",
```

```
                                 other_allele_col = "ref",
                                 eaf_col = "maf",
                                 pval_col = "pval",
                                 beta_col = "beta",
                                 se_col = "sebeta")
  while(TRUE){
    message_to_next <<- TRUE
    error_to_next <<- FALSE
    try({withCallingHandlers(exp_clumped<-clump_data(exp),
                                          message = function(c) if
(stringr::str_detect(as.character(c),"Failed to")) message_to_next <<- FALSE)
      error_to_next <<- TRUE})
    if(message_to_next == TRUE&error_to_next == TRUE) { break }
  }

}else{
  while(TRUE){
    message_to_next <<- TRUE
    error_to_next <<- FALSE
    try({withCallingHandlers(exp_clumped<-extract_instruments(outcomes = id,p1 = 1e-
05,access_token = NULL),
                                          message = function(c) if
(stringr::str_detect(as.character(c),"Failed to")) message_to_next <<- FALSE)
      error_to_next <<- TRUE})
    if(message_to_next == TRUE&error_to_next == TRUE) { break }
  }

}
table(!((((exp_clumped$beta.exposure)^2)/((exp_clumped$se.exposure)^2))<10))
exp_clumped <-
exp_clumped[!((((exp_clumped$beta.exposure)^2)/((exp_clumped$se.exposure)^2))<10)
,]

dat<-list()
for (i in 1:length(id_exp$id)) {
  print(id_exp$id[i])
  while(TRUE){
    message_to_next <<- TRUE
    error_to_next <<- FALSE
    try({withCallingHandlers(outcome<-extract_outcome_data(exp_clumped$SNP,
                                                           outcomes =
id_exp$id[i],
                                                           proxies = F,
                                                           access_token = NULL),
```

```
                                    message              =              function(c)              if
(stringr::str_detect(as.character(c),"Failed to")) message_to_next <<- FALSE)
      error_to_next <<- TRUE})
    if(message_to_next == TRUE&error_to_next == TRUE) { break }
  }
  if(is.null(outcome))next
  dat[[i]]<-harmonise_data(exposure_dat = exp_clumped,outcome_dat = outcome)
}
dat<-do.call(rbind,dat)

dat<-dat[dat$mr_keep,]
write.csv(dat,file = "re_dat.csv",row.names = F)

re_res<-mr(dat)
write.csv(re_res,file = "re_res.csv",row.names = F)
```

# Plotting

```
res_id<-read.csv("./res.csv")%>%get_sbeta_res()%>%dplyr::filter(method=="Inverse
variance weighted",pval<0.05)%>%pull(id.exposure)
dat_total<-read.csv("./dat.csv")
for (idr in res_id) {
  dat<-subset(dat_total,id.exposure==idr)
  scatter_plot<-mr_scatter_plot(mr(dat),dat)[[1]]+
    scale_color_lancet()+
    scale_fill_lancet()+
    theme(axis.title.y = element_text(size = 20))+
    theme_bw(base_size = 16)+ theme(
      plot.margin = margin(0.5,0.5,0.5,0.5, unit = "cm")
    )
  ggsave(plot=scatter_plot,filename    =    paste0(idr,"_scatter_plot_plot.pdf"),device    =
"pdf",width = 10,height = 10)
  funnel_plot<-
mr_funnel_plot(mr_singlesnp(dat,all_method=c("mr_egger_regression","mr_weighted_m
edian","mr_ivw","mr_simple_mode","mr_weighted_mode")))[[1]]+
    scale_color_lancet()+
    scale_fill_lancet()+
    theme(axis.title.y = element_text(size = 20))+
    theme_bw(base_size = 16)+ theme(
      plot.margin = margin(0.5,0.5,0.5,0.5, unit = "cm")
    )
  ggsave(plot=funnel_plot,filename    =    paste0(idr,"_funnel_plot_plot.pdf"),device    =
"pdf",width = 10,height = 10)
  forest_plot<-mr_forest_plot(mr_singlesnp(dat))[[1]]+
    scale_color_lancet()+
```

```
    scale_fill_lancet()+
    theme_bw()+
    theme(legend.position = 'none',plot.margin = margin(0.5,0.5,0.5,0.5, unit = "cm"))
  ggsave(plot=forest_plot,filename = paste0(idr,"_forest_plot_plot.pdf"),device = "pdf",width = 10,height = 10)

  leaveoneout_plot<-mr_leaveoneout_plot(mr_leaveoneout(dat))[[1]]+
    scale_color_lancet()+
    scale_fill_lancet()+
    theme_bw()+
    theme(legend.position = 'none',plot.margin = margin(0.5,0.5,0.5,0.5, unit = "cm"),
          axis.title.x = element_text(size = 12))
  ggsave(plot=leaveoneout_plot,filename = paste0(idr,"_leaveoneout_plot_plot.pdf"),device = "pdf",width = 10,height = 10)
}
```

# Forest plot

```
res_sign<-read.csv("./res.csv")%>%dplyr::filter(id.exposure%in%res_id)
res_sign$exposure<-str_split(str_split(res_sign$exposure,regex("\\(|\\)"),simplify = T)[,2]," id.",simplify = T)[,1]
res_sign$`OR (95% CI)` <- sprintf("%.3f (%.3f - %.3f)",res_sign$or, res_sign$or_lci95, res_sign$or_uci95)

res_sign$outcome[duplicated(res_sign$outcome)]<-""

dt<-res_sign[,c(3:6,9,12:14,15)]

dt$` ` <- paste(rep(" ", 30), collapse = " ")

dt$pval<-round(dt$pval,digits = 3)
dt$pval<-ifelse(dt$pval<0.001,"<0.001",dt$pval)

colnames(dt)[5]<-"italic(P)*-Value"

dt<-dt%>%dplyr::rename(Outcome=outcome,
                       `Trait`=exposure,
                       Method=method,
                       nSNP=nsnp,)

dt$Method<-ifelse(dt$Method=="Inverse variance weighted","IVW",dt$Method)

tm <- forest_theme(base_size = 8,colhead=list(fg_params = list(parse=TRUE)))
p=forest(dt[,c(2:5,10,9)],
         est = dt$or,
```

```
        lower = dt$or_lci95,
        upper = dt$or_uci95,
        sizes = 0.6,
        ci_column = 5,
        ref_line = 1,
        xlim = c(0,3),
        ticks_at = c(0,0.5,1,2,3),
        theme = tm)

ggsave("GM forest plot.pdf",plot=p,device = "pdf",width = 8,height = 8)
```

Step 3: Cyclic heat map

```
if(!require("gwasrapidd")) install.packages("gwasrapidd",update = F,ask = F)
if(!require("stringr")) install.packages("stringr",update = F,ask = F)
if(!require("data.table")) install.packages("data.table",update = F,ask = F)
if (!require("devtools")) { install.packages("devtools") } else {}
# devtools::install_github("rondolab/MR-PRESSO",force = TRUE)
library(TwoSampleMR)
library(MRPRESSO)
library(circlize)
library(stringr)
library(ComplexHeatmap)
library(RColorBrewer)

dat_total<-read.csv("./dat.csv")

i=1
result<-list()
for (file in unique(dat_total$id.exposure)) {
  print(which(file==unique(dat_total$id.exposure)))
  print(file)
  dat <- subset(dat_total,id.exposure==file)
  dat <- dat[dat$mr_keep,]
  if(nrow(dat)<2)next

  res <- generate_odds_ratios(mr_res = mr(dat,method_list =
c("mr_ivw","mr_egger_regression","mr_weighted_median")))

  res_hete <- mr_heterogeneity(dat)
  res_t <- cbind(merge(x = res,y = res_hete,by = "method",all.x =
T),mr_pleiotropy_test(dat)[,c("egger_intercept","pval")])

  if(length(dat$SNP)<4){res_t$mp<-NA}else{
  res_t$mp<-mr_presso(BetaOutcome = "beta.outcome",
                      BetaExposure = "beta.exposure",
                      SdOutcome = "se.outcome",
                      SdExposure = "se.exposure",
                      OUTLIERtest = TRUE,
                      DISTORTIONtest = TRUE,
                      data = dat,
                      NbDistribution = 1000,
                      SignifThreshold = 0.05)$`MR-PRESSO results`$`Global
Test`$Pvalue
  }
  result[[i]]<-res_t
```

```
  i=i+1
}
result_all <- do.call(rbind,result)
write.csv(result_all,file = "result_all.csv")

result_all<-read.csv("result_all.csv")

IVW<-result_all[result_all$method=="Inverse variance weighted",][,c(6,10,13,22,24,25)]
colnames(IVW)<-c("bac","IVW P-Value","Odd Ratio","Q P-Value","Egger intercept P-
Value","MRPRESSO Global Test P-Value")
IVW$`MRPRESSO Global Test P-Value`<-ifelse(IVW$`MRPRESSO Global Test P-
Value`=="<0.001",0.001,IVW$`MRPRESSO Global Test P-Value`)
IVW$`MRPRESSO Global Test P-Value`<-as.numeric(IVW$`MRPRESSO Global Test P-
Value`)
MREGGER<-result_all[result_all$method=="MR Egger",][,c(6,10)]
colnames(MREGGER)<-c("bac","MR Egger P-Value")
WM<-result_all[result_all$method=="Weighted median",][,c(6,10)]
colnames(WM)<-c("bac","WM P-Value")
data<-merge(x=IVW,y=MREGGER,by = "bac")
data<-merge(x=data,y=WM,by = "bac")
rownames(data) <- data[,1]
data<-data[,-1]
data<-as.matrix(data)
rownames(data)<-str_split(str_split(rownames(data),regex("\\(|\\)"),simplify = T)[,2],"
id.",simplify = T)[,1]

pdf(file = "1.pdf",width = 15,height = 15)

pal<-brewer.pal(11,'Spectral')
col_pval = colorRamp2(c(0, 0.05, 1), c("#9E0142","#FFFFBF","#5E4FA2"))

split_bac<-factor(str_split(rownames(data)," ",simplify = T)[,1],levels =
unique(str_split(rownames(data)," ",simplify = T)[,1]))

circos.clear()
circos.par(start.degree = 90, gap.degree = 5,gap.after = c(2,2,2,2,60),track.height =
0.1,clock.wise=F,circle.margin=0.75)
circos.heatmap.initialize(data,split = split_bac, cluster = F)

circos.heatmap(data[,1], col = col_pval,track.height = 0.05)
circos.track(track.index = get.current.track.index(), panel.fun = function(x, y) {
  if(CELL_META$sector.numeric.index == 1) { # the last sector
    cn = colnames(data)[1]
    circos.text(CELL_META$cell.xlim[2] + convert_x(2, "mm"),
```

```
                        CELL_META$ycenter, cn,
                        cex = 0.5, adj = c(0, 0.5), facing = "inside",niceFacing = F)
    }
}, bg.border = NA)

circos.track(track.index = get.current.track.index(), panel.fun = function(x, y) {
    name <- rownames(data)[CELL_META$subset]
    name <- name[CELL_META$row_order]
    circos.text(seq_along(name)-
0.5,CELL_META$cell.ylim[2]+(convert_y(strwidth(name,units = "inches"), "inches"))/4+convert_y(2,"mm"),name,cex = 0.5,facing = "reverse.clockwise")
}, cell.padding = c(0.02, 0, 0.02, 0))

circos.heatmap(data[,6], col = col_pval,bg.border = "black",bg.lwd = 1, bg.lty = 1,track.height = 0.05)
circos.track(track.index = get.current.track.index(), panel.fun = function(x, y) {
    if(CELL_META$sector.numeric.index == 1) { # the last sector
        cn = colnames(data)[6]
        circos.text(CELL_META$cell.xlim[2] + convert_x(2, "mm"),
                        CELL_META$ycenter, cn,
                        cex = 0.5, adj = c(0, 0.5), facing = "inside",niceFacing = F)
    }
}, bg.border = NA)

circos.heatmap(data[,7], col = col_pval,bg.border = "black",bg.lwd = 1, bg.lty = 1,track.height = 0.05)
circos.track(track.index = get.current.track.index(), panel.fun = function(x, y) {
    if(CELL_META$sector.numeric.index == 1) { # the last sector
        cn = colnames(data)[7]
        circos.text(CELL_META$cell.xlim[2] + convert_x(2, "mm"),
                        CELL_META$ycenter, cn,
                        cex = 0.5, adj = c(0, 0.5), facing = "inside",niceFacing = F)
    }
}, bg.border = NA)

circos.heatmap(data[,4], col = col_pval,bg.border = "black",bg.lwd = 1, bg.lty = 1,track.height = 0.05)
circos.track(track.index = get.current.track.index(), panel.fun = function(x, y) {
    if(CELL_META$sector.numeric.index == 1) { # the last sector
        cn = colnames(data)[4]
        circos.text(CELL_META$cell.xlim[2] + convert_x(2, "mm"),
                        CELL_META$ycenter, cn,
                        cex = 0.5, adj = c(0, 0.5), facing = "inside",niceFacing = F)
    }
```

```
}, bg.border = NA)

circos.heatmap(data[,5], col = col_pval,bg.border = "black",bg.lwd = 1, bg.lty =
1,track.height = 0.05)
circos.track(track.index = get.current.track.index(), panel.fun = function(x, y) {
  if(CELL_META$sector.numeric.index == 1) { # the last sector
    cn = colnames(data)[5]
    circos.text(CELL_META$cell.xlim[2] + convert_x(2, "mm"),
                CELL_META$ycenter, cn,
                cex = 0.5, adj = c(0, 0.5), facing = "inside",niceFacing = F)
  }
}, bg.border = NA)

circos.heatmap(data[,3], col = col_pval,bg.border = "black",bg.lwd = 1, bg.lty =
1,track.height = 0.05)
circos.track(track.index = get.current.track.index(), panel.fun = function(x, y) {
  if(CELL_META$sector.numeric.index == 1) { # the last sector
    cn = colnames(data)[3]
    circos.text(CELL_META$cell.xlim[2] + convert_x(2, "mm"),
                CELL_META$ycenter, cn,
                cex = 0.5, adj = c(0, 0.5), facing = "inside",niceFacing = F)
  }
}, bg.border = NA)

row_or = data[,2]
circos.track(ylim = range(row_or), panel.fun = function(x, y) {
  y = row_or[CELL_META$subset]
  y = y[CELL_META$row_order]
  circos.lines(CELL_META$cell.xlim, c(1,1), lty = 2, col = "#7570B3")
  circos.points(seq_along(y)-0.5, y, col = ifelse(y < 1, "#1B9E77", "#D95F02"),cex =
0.5,pch = 16)
}, cell.padding = c(0.02, 0, 0.02, 0))
circos.track(track.index = get.current.track.index(), panel.fun = function(x, y) {
  if(CELL_META$sector.numeric.index == 1) { # the last sector
    cn = colnames(data)[2]
    circos.text(CELL_META$cell.xlim[2] + convert_x(2, "mm"),
                CELL_META$ycenter, cn,
                cex = 0.5, adj = c(0, 0.5), facing = "inside",niceFacing = F)
  }
}, bg.border = NA)
circos.yaxis( side = "left",labels.cex = 0.5,at = c(0.7,1,1.3))

circos.track(track.index = get.current.track.index(), panel.fun = function(x, y) {
  circos.text(CELL_META$xcenter, CELL_META$cell.ylim[1] - convert_y(3, "mm"),
```

```
                CELL_META$sector.index,
                facing = "bending.inside", cex = 0.5,
                adj = c(0.5, 0), niceFacing = TRUE)
}, bg.border = NA)

pval = Legend(title = "P.Value", col_fun = col_pval,at = c(0,0.05,0.5,1))
or = Legend(labels = c("Risk factor","Protective factor"), title = "Odd Ratio", legend_gp =
gpar(fill=c("#D95F02", "#1B9E77")))
all<-packLegend(pval,or)
grid.draw(all)
dev.off()
```

```
### Step 4: Plasma metabolites on Osteoporosis
# Load R packages
rm(list = ls())
library(dplyr)
library(MRPRESSO)
library(TwoSampleMR)
library(dplyr)
library(ggsci)
library(data.table)
library(ggplot2)
library(ggpubr)
library(ggview)
library(plotly)
library(TSMRhelper)
library(forestploter)
library(grid)
library(stringr)
library(grDevices)
library(parallel)

# Read the local outcome (Osteoporosis)
id<-""
outcome_all<-fread("./finngen_R10_M13_OSTEOPOROSIS.gz ",data.table = F)

# Get full Plasma metabolites IEU exposure id data
ao<-read.csv("ao.csv")
id_exp<-subset(ao,pmid== 36635386)
id_exp<-id_exp[!str_detect(id_exp$trait,"unknown"),]
exp<-list()
i <- 1
for (ids in id_exp[1:nrow(id_exp),]$id) {
  print(ids)
  while(TRUE){
    message_to_next <<- TRUE
    error_to_next <<- FALSE
    try({withCallingHandlers(exp_clumped<-extract_instruments(outcomes = ids,
                                                              p1 = 1e-05,
                                                              clump = TRUE,
                                                              r2 = 0.1,
                                                              kb = 500,
                                                              access_token =
NULL),
                             message = function(c) if
(stringr::str_detect(as.character(c),"Failed to")) message_to_next <<- FALSE)
```

```
         error_to_next <<- TRUE})
      if(message_to_next == TRUE&error_to_next == TRUE) { break }
   }
   if(is.null(exp_clumped))next
   exp_clumped$exposure<-id_exp[id_exp$id==exp_clumped$id.exposure[1],]$trait
   exp[[i]]<-exp_clumped
   i<-i+1
}
exp_clumped<-do.call(rbind,exp)

write.csv(exp_clumped,file = 1400_exp_clumped_1e05_0.1_500.csv ",row.names = F)

# Calculating the F-statistic
exp_clumped<-read.csv("1400_exp_clumped_1e05_0.1_500.csv ")
F_cal<-function(beta,se){F.Statistics<-beta^2/se^2}
F.Statistics<-F_cal(beta =exp_clumped$beta.exposure,se =exp_clumped$se.exposure )
exp_clumped$F.Statistics <- F.Statistics

# Get outcome
if(id==""){
   snp<-unique(exp_clumped$SNP)
   outcome<-outcome_all[outcome_all$rsids%in%snp,]
   outcome_dat<-format_data(outcome,
                            type = "outcome",
                            beta_col = "beta",
                            se_col = "sebeta",
                            effect_allele_col = "alt",
                            other_allele_col = "ref",
                            snp_col = "rsids",
                            chr_col = "#chrom",
                            pos_col = "pos",
                            pval_col = "pval",
                            eaf_col = "af_alt")
}else{
   #
   while(TRUE){
      message_to_next <<- TRUE
      error_to_next <<- FALSE
      try({withCallingHandlers(outcome_dat <- extract_outcome_data(snps =
unique(exp_clumped$SNP),
                                                outcomes = id,
                                                proxies = F,
                                                access_token =
NULL,
```

```
                                                    splitsize          =
50000
      ),
      message   =   function(c)   if   (stringr::str_detect(as.character(c),"Failed   to"))
message_to_next <<- FALSE)
         error_to_next <<- TRUE})
      if(message_to_next == TRUE&error_to_next == TRUE) { break }
   }
}

# Harmonise data
dat <- harmonise_data(exposure_dat = exp_clumped,outcome_dat = outcome_dat)
dat<-dat[dat$mr_keep,]
write.csv(dat,file = "1400_dat.csv",row.names = F)

# MR analysis
dat<-split(dat,dat$id.exposure)
library(parallel)
cl <- makeCluster(detectCores())
res<-parLapply(cl,dat,mr)
stopCluster(cl)
res<-do.call(rbind,res)
res<-generate_odds_ratios(res)
write.csv(res,file = "1400_res.csv",row.names = F)
dat<-do.call(rbind,dat)

# Heterogeneity analysis
heterogeneity<-mr_heterogeneity(dat)
write.csv(heterogeneity,file = "1400_heterogeneity.csv",row.names = F)

# Pleiotropy test
pleiotropy_test<-mr_pleiotropy_test(dat)
write.csv(pleiotropy_test,file = "1400_pleiotropy_test.csv",row.names = F)

# MR-PRESSO analysis
mps<-res%>%get_sbeta_res()%>%dplyr::filter(method=="Inverse                variance
weighted",pval<0.05)%>%pull(id.exposure)

mp<-list()
i=1
for (mpi in mps) {
   data<-dat[dat$id.exposure==mpi,]
   if(nrow(data)<4){next}
   # MRPRESSO
```

```
  mp[[i]]<-mr_presso(BetaOutcome = "beta.outcome",
                     BetaExposure = "beta.exposure",
                     SdOutcome = "se.outcome",
                     SdExposure = "se.exposure",
                     OUTLIERtest = TRUE,
                     DISTORTIONtest = TRUE,
                     data = data,
                     NbDistribution = 1000,
                     SignifThreshold = 0.05)
  mp[[i]]$phe <- data$exposure[1]
  i=i+1
}
result_mp_all<- data.frame()
for (i in 1:length(mp)) {
  print(mp[[i]]$phe)
  result_mp <- mp[[i]]$`Main MR results`
  result_mp$RSSobs <- mp[[i]]$`MR-PRESSO results`$`Global Test`$RSSobs
  result_mp$Pvalue <- mp[[i]]$`MR-PRESSO results`$`Global Test`$Pvalue
  result_mp$phe <- mp[[i]]$phe
  result_mp_all <- rbind(result_mp_all,result_mp)
}
table(is.na(result_mp_all$`Causal Estimate`))
write.csv(result_mp_all,file = "1400_MRPRESSO.csv")

# Reverse MR analysis
if(id==""){
  exp<-outcome_all
  exp$pval<-as.numeric(exp$pval)
  exp<-subset(exp,pval<1e-05)
  exp<-format_data(exp,
                   snp_col = "rsids",
                   chr_col = "#chrom",
                   pos_col = "pos",
                   effect_allele_col = "alt",
                   other_allele_col = "ref",
                   eaf_col = "af_alt",
                   pval_col = "pval",
                   beta_col = "beta",
                   se_col = "sebeta")
  while(TRUE){
    message_to_next <<- TRUE
    error_to_next <<- FALSE
    try({withCallingHandlers(exp_clumped<-clump_data(exp),
                             message = function(c) if
```

```
(stringr::str_detect(as.character(c),"Failed to")) message_to_next <<- FALSE)
          error_to_next <<- TRUE})
      if(message_to_next == TRUE&error_to_next == TRUE) { break }
   }

}else{
   while(TRUE){
      message_to_next <<- TRUE
      error_to_next <<- FALSE
      try({withCallingHandlers(exp_clumped<-extract_instruments(outcomes = id,p1 = 1e-
05,access_token = NULL),
                                             message = function(c) if
(stringr::str_detect(as.character(c),"Failed to")) message_to_next <<- FALSE)
          error_to_next <<- TRUE})
      if(message_to_next == TRUE&error_to_next == TRUE) { break }
   }

}
table(!((((exp_clumped$beta.exposure)^2)/((exp_clumped$se.exposure)^2))<10))
exp_clumped <-
exp_clumped[!((((exp_clumped$beta.exposure)^2)/((exp_clumped$se.exposure)^2))<10)
,]

idTotrait<-read.csv("./idTotrait.csv")
total_data<-dir("./data1400/")
dat<-list()
for (i in 1:length(total_data)) {
   print(total_data[i])
   outcome_dat<-fread(paste0("./data1400/",total_data[i]),data.table = F)
   outcome_dat$p_value<-as.numeric(outcome_dat$p_value)

   outcome_dat<-subset(outcome_dat,`variant_id`%in%unique(exp_clumped$SNP))
   outcome_dat$id <- str_split(total_data[i],"_buildGRCh38.tsv.gz",simplify = T)[1]
   outcome_dat$phe<-idTotrait[idTotrait$ID==outcome_dat$id[1],]$Trait
   outcome_dat<-format_data(outcome_dat,
                                          type = "outcome",
                                          snp_col = "variant_id",
                                          beta_col = "beta",
                                          se_col = "standard_error",
                                          eaf_col = "effect_allele_frequency",
                                          effect_allele_col = "effect_allele",
                                          other_allele_col = "other_allele",
                                          pval_col = "p_value",
                                          chr_col = "chromosome",
```

```
                                        pos_col = "base_pair_location",
                                        id_col = "id",
                                        phenotype_col = "phe")
  dat[[i]]<-harmonise_data(exposure_dat = exp_clumped,outcome_dat = outcome_dat)
}
dat<-do.call(rbind,dat)

dat<-dat[dat$mr_keep,]
write.csv(dat,file = "C_1400_re_dat.csv",row.names = F)

re_res<-mr(dat)
write.csv(re_res,file = "C_1400_re_res.csv",row.names = F)
```

# Plotting

```
res_id<-
read.csv("./1400_res.csv")%>%get_sbeta_res()%>%dplyr::filter(method=="Inverse
variance weighted",pval<0.05)%>%pull(id.exposure)
dat_total<-read.csv("./1400_dat.csv")
for (idr in res_id) {
  dat<-subset(dat_total,id.exposure==idr)
  scatter_plot<-mr_scatter_plot(mr(dat),dat)[[1]]+
    scale_color_lancet()+
    scale_fill_lancet()+
    theme(axis.title.y = element_text(size = 20))+
    theme_bw(base_size = 16)+ theme(
      plot.margin = margin(0.5,0.5,0.5,0.5, unit = "cm")
    )
  ggsave(plot=scatter_plot,filename = paste0(idr,"_scatter_plot_plot.pdf"),device =
"pdf",width = 10,height = 10)
  funnel_plot<-
mr_funnel_plot(mr_singlesnp(dat,all_method=c("mr_egger_regression","mr_weighted_m
edian","mr_ivw","mr_simple_mode","mr_weighted_mode")))[[1]]+
    scale_color_lancet()+
    scale_fill_lancet()+
    theme(axis.title.y = element_text(size = 20))+
    theme_bw(base_size = 16)+ theme(
      plot.margin = margin(0.5,0.5,0.5,0.5, unit = "cm")
    )
  ggsave(plot=funnel_plot,filename = paste0(idr,"_funnel_plot_plot.pdf"),device =
"pdf",width = 10,height = 10)
  forest_plot<-mr_forest_plot(mr_singlesnp(dat))[[1]]+
    scale_color_lancet()+
    scale_fill_lancet()+
    theme_bw()+
```

```
    theme(legend.position = 'none',plot.margin = margin(0.5,0.5,0.5,0.5, unit = "cm"))
  ggsave(plot=forest_plot,filename = paste0(idr,"_forest_plot_plot.pdf"),device = "pdf",width = 10,height = 10)

  leaveoneout_plot<-mr_leaveoneout_plot(mr_leaveoneout(dat))[[1]]+
    scale_color_lancet()+
    scale_fill_lancet()+
    theme_bw()+
    theme(legend.position = 'none',plot.margin = margin(0.5,0.5,0.5,0.5, unit = "cm"),
          axis.title.x = element_text(size = 12))
  ggsave(plot=leaveoneout_plot,filename = paste0(idr,"_leaveoneout_plot_plot.pdf"),device = "pdf",width = 10,height = 10)
}
```

# Forest plot

```
res_sign<-read.csv("./1400_res.csv")%>%dplyr::filter(id.exposure%in%res_id)
res_sign$`OR (95% CI)` <- sprintf("%.3f (%.3f - %.3f)",res_sign$or, res_sign$or_lci95, res_sign$or_uci95)


res_sign$outcome[duplicated(res_sign$outcome)]<-""

dt<-res_sign[,c(3:6,9,12:14,15)]

dt$` ` <- paste(rep(" ", 30), collapse = " ")

dt$pval<-round(dt$pval,digits = 3)
dt$pval<-ifelse(dt$pval<0.001,"<0.001",dt$pval)

colnames(dt)[5]<-"italic(P)*-Value"

dt<-dt%>%dplyr::rename(Outcome=outcome,
                       `Trait`=exposure,
                       Method=method,
                       nSNP=nsnp,)

dt$Method<-ifelse(dt$Method=="Inverse variance weighted","IVW",dt$Method)
dt<-dt[dt$Method=="IVW", ]

tm <- forest_theme(base_size = 8,colhead=list(fg_params = list(parse=TRUE)))
p=forest(dt[,c(2:5,10,9)],
         est = dt$or,
         lower = dt$or_lci95,
         upper = dt$or_uci95,
```

```
          sizes = 0.6,
          ci_column = 5,
          ref_line = 1,
          xlim = c(0.5,1.5),
          ticks_at = c(0.5,0.75,1,1.25,1.5),
          theme = tm)

ggsave("1400 forest plot.pdf",plot=p,device = "pdf",width = 20,height = 20)
```

```
### Step 5: GM on Plasma metabolites
# Load R packages
rm(list = ls())
library(TSMRhelper)
library(dplyr)

# Read data
gut_res<-read.csv("./res.csv")%>%get_sbeta_res()%>%dplyr::filter(method=="Inverse
variance weighted",pval<0.05)
res1400<-
read.csv("./1400_res.csv")%>%get_sbeta_res()%>%dplyr::filter(method=="Inverse
variance weighted",pval<0.05)
gut_exp<-
read.csv("exp_clumped.csv")%>%dplyr::filter(id.exposure%in%gut_res$id.exposure)
idTotrait<-read.csv("./idTotrait.csv")
ids<-paste0(res1400$id.exposure,"_buildGRCh38.tsv.gz")
dat<-list()
for (i in 1:length(ids)) {
  print(ids[i])
  outcome_dat<-fread(paste0("./data1400/",ids[i]),data.table = F)
  outcome_dat$p_value<-as.numeric(outcome_dat$p_value)

# Screening for SNPs
outcome_dat<-subset(outcome_dat,`variant_id`%in%unique(gut_exp$SNP))
  outcome_dat$id <- str_split(ids[i],"_buildGRCh38.tsv.gz",simplify = T)[1]
  outcome_dat$phe<-idTotrait[idTotrait$ID==outcome_dat$id[1],]$Trait
  outcome_dat<-format_data(outcome_dat,
                           type = "outcome",
                           snp_col = "variant_id",
                           beta_col = "beta",
                           se_col = "standard_error",
                           eaf_col = "effect_allele_frequency",
                           effect_allele_col = "effect_allele",
                           other_allele_col = "other_allele",
                           pval_col = "p_value",
                           chr_col = "chromosome",
                           pos_col = "base_pair_location",
                           id_col = "id",
                           phenotype_col = "phe")
  dat[[i]]<-harmonise_data(exposure_dat = gut_exp,outcome_dat = outcome_dat)
}
dat<-do.call(rbind,dat)
dat<-dat[dat$mr_keep,]
write.csv(dat,file = "gutto1400_dat.csv",row.names = F)
```

```
dat<-split(dat,dat$id.exposure)
library(parallel)
cl <- makeCluster(detectCores())
res<-parLapply(cl,dat,mr)
stopCluster(cl)
res<-do.call(rbind,res)
write.csv(res,file = "gutto1400_res.csv",row.names = F)
```

# Possible mediation

```
id_final<-res%>%dplyr::filter(method=="Inverse variance weighted",pval<0.05)

for (a in 1:nrow(id_final)) {
  print(paste0("exposure：",id_final[a,]$id.exposure," mediation：",id_final[a,]$id.outcome))
}
```

```
### Step 6: Multivariate MR analysis
# Load R packages
library(TwoSampleMR)
library(data.table)
library(dplyr)
library(RMediation)
library(stringr)

# To run multiple variables separately, please fill in the corresponding id numbers separately


# GM id example
ids<-"ebi-a-GCST90016925"

# Plasma metabolite id example
id1400<-"GCST90200756"

# outcome
out_id<-""
out_dat<-fread("./finngen_R10_M13_OSTEOPOROSIS.gz",data.table = F)
idTotrait<-read.csv("./idTotrait.csv")

res_all<-list()
i=1
for (id in ids) {
exp1<-fread(paste0("./data1400/",id1400,"_buildGRCh38.tsv.gz"),data.table = F)
exp1$`p_value`<-as.numeric(exp1$`p_value`)
exp1$id <- id1400
exp1$phe<-idTotrait[idTotrait$ID==exp1$id[1],]$Trait
exp1<-format_data(exp1,
              type = "outcome",
              snp_col = "variant_id",
              beta_col = "beta",
              se_col = "standard_error",
              eaf_col = "effect_allele_frequency",
              effect_allele_col = "effect_allele",
              other_allele_col = "other_allele",
              pval_col = "p_value",
              chr_col = "chromosome",
              pos_col = "base_pair_location",
              id_col = "id",
              phenotype_col = "phe")
```

```
exp1_1 <- subset(exp1, pval.outcome < 1e-05)
out <- ieugwasr::ld_clump(dplyr::tibble(rsid=exp1_1$SNP, pval=exp1_1$pval.outcome,id = exp1_1$id.outcome),
                          clump_kb = 10000,
                          clump_r2 = 0.001,
                          clump_p = 1e-05,
                          plink_bin = plinkbinr::get_plink_exe(),
                          bfile = "F:/1kg.v3/EUR",
                          pop = "EUR")
keep <- paste(exp1_1$SNP,exp1_1$id.outcome) %in% paste(out$rsid,out$id)
exp1_1 <- exp1_1[keep,]
exp1_1<-format_data(exp1_1,
                    type = "exposure",
                    snp_col = "SNP",
                    chr_col = "chr.outcome",
                    pos_col = "pos.outcome",
                    beta_col = "beta.outcome",
                    se_col = "se.outcome",
                    pval_col = "pval.outcome",
                    samplesize_col = "samplesize.outcome",
                    id_col = "id.outcome",
                    effect_allele_col = "effect_allele.outcome",
                    other_allele_col = "other_allele.outcome",
                    eaf_col = "eaf.outcome",
                    phenotype_col = "outcome")
while(TRUE){
  message_to_next <<- TRUE
  error_to_next <<- FALSE
  try({withCallingHandlers(exp2<-extract_instruments(outcomes = id,p1 = 1e-05,access_token = NULL),
                           message = function(c) if (stringr::str_detect(as.character(c),"Failed to")) message_to_next <<- FALSE)
    error_to_next <<- TRUE})
  if(message_to_next == TRUE&error_to_next == TRUE) { break }
}
exp2$chr.exposure<-as.numeric(exp2$chr.exposure)
exposure_dat <- dplyr::bind_rows(list(exp1_1,exp2))
id_exposure <- unique(exposure_dat$id.exposure)
temp <- exposure_dat
temp$id.exposure <- 1
temp <- temp[order(temp$pval.exposure, decreasing = FALSE), ]
temp <- subset(temp, !duplicated(SNP))
out <- ieugwasr::ld_clump(dplyr::tibble(rsid=temp$SNP, pval=temp$pval.exposure,id = temp$id.exposure),
```

```
                             clump_kb = 10000,
                             clump_r2 = 0.001,
                             clump_p = 1e-05,
                             plink_bin = plinkbinr::get_plink_exe(),
                             bfile = "F:/1kg.v3/EUR",
                             pop = "EUR")
keep <- paste(temp$SNP,temp$id.exposure) %in% paste(out$rsid,out$id)
temp <- temp[keep,]
exposure_dat <- subset(exposure_dat, SNP %in% temp$SNP)
```

# Retrieve snp

```
exp1_2<-subset(exp1, SNP %in% exposure_dat$SNP)
while(TRUE){
  message_to_next <<- TRUE
  error_to_next <<- FALSE
  try({withCallingHandlers(exp2_2<-extract_outcome_data(snps =
exposure_dat$SNP,outcomes = id,proxies = F,access_token = NULL),
                             message = function(c) if
(stringr::str_detect(as.character(c),"Failed to")) message_to_next <<- FALSE)
    error_to_next <<- TRUE})
  if(message_to_next == TRUE&error_to_next == TRUE) { break }
}
exp1_2<-exp1_2%>%dplyr::rename(
  chr=`chr.outcome`,
  pos=`pos.outcome`
)
exp2_2$chr<-as.numeric(exp2_2$chr)
exp2_2$pos<-as.numeric(exp2_2$pos)
d1<-dplyr::bind_rows(list(exp1_2,exp2_2))
d1 <- subset(d1, mr_keep.outcome)
d2 <- subset(d1, id.outcome != id_exposure[1])
d1 <- convert_outcome_to_exposure(subset(d1, id.outcome ==
                                                  id_exposure[1]))
d <- harmonise_data(d1, d2)
tab <- table(d$SNP)
keepsnps <- names(tab)[tab == length(id_exposure) - 1]
d <- subset(d, SNP %in% keepsnps)
dh1 <- subset(d, id.outcome == id.outcome[1], select = c(SNP,
                                                  exposure, id.exposure,
effect_allele.exposure, other_allele.exposure,
                                                  eaf.exposure,
beta.exposure, se.exposure, pval.exposure))
dh2 <- subset(d, select = c(SNP, outcome, id.outcome, effect_allele.outcome,
                             other_allele.outcome, eaf.outcome, beta.outcome,
```

```
se.outcome,
                                        pval.outcome))

names(dh2) <- gsub("outcome", "exposure", names(dh2))
exp_dat <- rbind(dh1, dh2)

if(out_id==""){
   out_dat<-subset(out_dat,rsids%in%exp_dat$SNP)
   out_dat<-format_data(out_dat,
                            type = "outcome",
                            beta_col = "beta",
                            se_col = "sebeta",
                            effect_allele_col = "alt",
                            other_allele_col = "ref",
                            snp_col = "rsids",
                            chr_col = "#chrom",
                            pos_col = "pos",
                            pval_col = "pval",
                            eaf_col = "af_alt")



}else{

   while(TRUE){
      message_to_next <<- TRUE
      error_to_next <<- FALSE
      try({withCallingHandlers(out_dat <- extract_outcome_data(snps = exp_dat$SNP,
                                                                              outcomes       =
out_id,
                                                                              proxies = F,
                                                                              access_token =
NULL
      ),
      message     =     function(c)     if     (stringr::str_detect(as.character(c),"Failed     to"))
message_to_next <<- FALSE)
         error_to_next <<- TRUE})
      if(message_to_next == TRUE&error_to_next == TRUE) { break }
   }
}

mvdat <- mv_harmonise_data(exposure_dat = exp_dat, outcome_dat = out_dat)
res <- mv_multiple(mvdat,pval_threshold = 1e-05)
res_all[[i]]<-res[["result"]]
i=i+1
```

```
}
res_all<-do.call(rbind,res_all)
if(res_all[!str_detect(res_all$exposure,"Gut microbiota abundance"),]$pval<0.05)print("mediated p-value adjusted to be less than 0.05")
write.csv(res_all,file = "res_all.csv")
```

# Getting outcome data for the same exposure

```
gut<-read.csv("./res.csv")%>%dplyr::filter(id.exposure==id,method=="Inverse variance weighted")
```

# Getting ending data from the same mediator

```
res1400<-
read.csv("./1400_res.csv")%>%dplyr::filter(id.exposure==id1400,method=="Inverse variance weighted")
```

# Getting the outcome data exposed to the mediator

```
gutto1400<-
read.csv("./gutto1400_res.csv")%>%dplyr::filter(id.exposure==id,id.outcome==id1400,method=="Inverse variance weighted")
```

# Calculating the mediating effect

```
EM_beta<- gutto1400$b

EM_se<- gutto1400$se

MO_beta<-res_all[!str_detect(res_all$exposure,"Gut microbiota abundance"),]$b

MO_se<-res_all[!str_detect(res_all$exposure,"Gut microbiota abundance"),]$se

# Delta
product_method_Delta <- function(EM_beta, EM_se, MO_beta, MO_se, verbose=F){


  # method 1
  # INDIRECT = TOTAL (exposure -> mediator) x TOTAL (mediator -> outcome)
  # method 2
  # INDIRECT = TOTAL (exposure -> mediator) x DIRECT (of mediator , mvmr)



  EO <- EM_beta * MO_beta

  if (verbose) {print(paste("Indirect effect = ", round(EM_beta, 2)," x ", round(MO_beta,2),
```

```r
" = ", round(EO, 3)))}


   CIs = medci(EM_beta, MO_beta, EM_se, MO_se, type="dop")

   df <-data.frame(b = EO,
                             se = CIs$SE,
                             lo_ci = CIs[["95% CI"]][1],
                             up_ci= CIs[["95% CI"]][2])
   # OR
   df$or          <-   exp(df$b)
   df$or_lci95 <- exp(df$lo_ci)
   df$or_uci95 <- exp(df$up_ci)

   df<-round(df,3)
   return(df)
}


#INDIRECT = TOTAL (exposure -> mediator) x DIRECT (of mediator , mvmr)
product_method_Delta(EM_beta, EM_se, MO_beta, MO_se)
```
